# Antimony efflux underpins phosphorus cycling and resistance of phosphate-solubilizing bacteria in mining soils

**DOI:** 10.1038/s41396-023-01445-6

**Published:** 2023-06-03

**Authors:** Shengwei Liu, Jiaxiong Zeng, Huang Yu, Cheng Wang, Yunfeng Yang, Jianjun Wang, Zhili He, Qingyun Yan

**Affiliations:** 1 Environmental Microbiomics Research Center, School of Environmental Science and Engineering, Southern Marine Science and Engineering Guangdong Laboratory (Zhuhai), State Key Laboratory for Biocontrol, Sun Yat-sen University, Guangzhou, 510006 China; 2 State Key Joint Laboratory of Environment Simulation and Pollution Control, School of Environment, Tsinghua University, 100084 Beijing, China; 3 State Key Laboratory of Lake Science and Environment, Nanjing Institute of Geography and Limnology, Chinese Academy of Sciences, Nanjing, 210008 China

**Keywords:** Microbial ecology, Soil microbiology

## Abstract

Microorganisms play crucial roles in phosphorus (P) turnover and P bioavailability increases in heavy metal-contaminated soils. However, microbially driven P-cycling processes and mechanisms of their resistance to heavy metal contaminants remain poorly understood. Here, we examined the possible survival strategies of P-cycling microorganisms in horizontal and vertical soil samples from the world’s largest antimony (Sb) mining site, which is located in Xikuangshan, China. We found that total soil Sb and pH were the primary factors affecting bacterial community diversity, structure and P-cycling traits. Bacteria with the *gcd* gene, encoding an enzyme responsible for gluconic acid production, largely correlated with inorganic phosphate (Pi) solubilization and significantly enhanced soil P bioavailability. Among the 106 nearly complete bacterial metagenome-assembled genomes (MAGs) recovered, 60.4% carried the *gcd* gene. Pi transportation systems encoded by *pit* or *pstSCAB* were widely present in *gcd*-harboring bacteria, and 43.8% of the *gcd*-harboring bacteria also carried the *acr3* gene encoding an Sb efflux pump. Phylogenetic and potential horizontal gene transfer (HGT) analyses of *acr3* indicated that Sb efflux could be a dominant resistance mechanism, and two *gcd*-harboring MAGs appeared to acquire *acr3* through HGT. The results indicated that Sb efflux could enhance P cycling and heavy metal resistance in Pi-solubilizing bacteria in mining soils. This study provides novel strategies for managing and remediating heavy metal-contaminated ecosystems.

## Introduction

Phosphorus (P) is an indispensable element for all life because it is present in various biological molecules [1]. However, deficiencies in bioavailable P are widespread in most terrestrial ecosystems [2, 3], especially in heavily degraded mining environments [4]. Scarcity of soil available phosphorus (AP) is prevalent, as soil P mainly exists as recalcitrant P forms that are sequestered in minerals or complex organic compounds, which cannot be directly assimilated by most organisms [5, 6]. To address AP scarcity, some P-cycling microorganisms in soils have developed different strategies to maintain and enhance P bioavailability, which could be leveraged to improve biological P acquisition and enhance primary soil productivity [7, 8]. Mining areas are one of the most nutrient-limited environments, which are contaminated with persistent heavy metals [9]. The absorption of soil P by plants that can enrich heavy metals will promote plant growth and microbial P-releasing strategies can increase their potential to be used for phytoremediation in soils polluted by heavy metals [10]. Therefore, understanding microbially driven soil P-cycling mechanisms is an important issue for managing and restoring heavily degraded environments [11].

The metalloid antimony (Sb) has been considered a priority pollutant regulated by the Environmental Protection Agency of the United States, the European Union and agencies in China [12]. Currently, China leads the world’s Sb production, at ~60,000 metric tons per year [13], resulting in severe Sb pollution in mining areas [12]. For example, the Sb concentration in the world’s largest Sb mining site, located in Xikuangshan (XKS, Hunan, China), can reach 302 mg kg^−1^ in soils [14], posing a severe threat to neighboring ecosystems [15]. The native microorganisms in Sb-contaminated soils can develop specific resistance strategies (e.g., oxidation, reduction and efflux) to survive and adapt to such contaminated environments [16]. The microbial oxidative transformation of antimonite to less toxic valence states is a prevalent strategy to improve microbial adaptability in oxic environments [17]. In anoxic environments, antimonate can be reduced to less-mobile antimonite by microorganisms and immobilized as stable forms (e.g., Sb_2_O_3_ and Sb_2_S_3_), decreasing its toxicity [18]. Furthermore, efflux pumps are known as the most direct and effective microbial resistance strategies [19], among which a membrane permease encoded by the *arsB* or *acr3* gene constitutes a major detoxification pathway for intracellular antimonite efflux [20, 21]. Microorganisms can also develop metabolic strategies to promote soil nitrogen (N) and P cycling under highly contaminated conditions. For example, *Serratia* spp. has been reported to fix N by coupling it with arsenite oxidation in arsenic (As)-rich mine tailings [22]. Moreover, inorganic phosphate (Pi)-solubilizing microorganisms contributed considerably to enhancing soil AP during the remediation of extremely acidic copper mine tailings [11]. Thus, microorganisms with resistance abilities in mining areas have great potential for enhancing soil nutrient cycling.

In heavy metal-contaminated and oligotrophic environments, bacteria are the major drivers of soil P cycling, which includes Pi solubilization, organic P mineralization, P transportation and regulation [23–25]. Proton secretion and production of organic acids are the main methods of bacterial Pi solubilization [26]; these processes release Pi from minerals (e.g., Al-Pi, Fe-Pi and Ca-Pi compounds) by reducing soil pH or chelating cations via their carboxyl and hydroxyl ions [27]. Oxidation from glucose to gluconic acid is one of the most frequent routes for mineral-sorbed Pi solubilization performed by quinoprotein glucose dehydrogenase encoded by the *gcd* gene [28, 29]. The *gcd*-harboring bacteria showed high functional diversity and metabolic potential, which might give them competitive advantages for surviving in harsh environments [30]. Soil bacteria can also cleave Pi from complex organophosphorus compounds by producing phosphatases [31], including alkaline phosphatase (AKP), acid phosphatase (ACP) and phytase [32]. Accordingly, to scavenge necessary P for growth, the microbial *pstS* gene encoding a substrate-binding protein for Pi transport systems was enriched in Sb-contaminated soils [33]. P and Sb belong to the same group in the periodic table and have similar chemical properties, and a recent study revealed that the microbial Pi-specific transporter PstS would be able to take up both Pi and antimonate into cells through the same binding site [34], leading to intracellular Sb accumulation and toxicity. To relieve toxic effects, intracellular antimonate would be reduced to antimonite by a reductase (ArsC) and subsequently exported by an efflux permease (ArsB or Acr3) [35]. However, the Sb-resistance mechanisms of P-cycling bacteria in Sb-contaminated soils remain unclear, largely limiting our understanding of how P-cycling bacteria adapt to Sb stress.

Previous studies on soil P bioavailability and microbial P cycling have mainly focused on soil fertility and crop production in agroecosystems [8, 36, 37]. However, microbial P cycling in mining areas is poorly understood, and few studies have explored functional genes involved in P cycling and heavy metal resistance [33, 38]. Our previous study showed that soil AP was a key driver of microbial communities in XKS [14]. Therefore, a further understanding of the possible mechanisms driving microbial P cycling and metal resistance would provide new insights for remediating heavy metal-contaminated soils. We hypothesized that (i) microbial Pi solubilization processes correlated with *gcd*-harboring bacteria would predominantly contribute to soil P bioavailability and (ii) P-cycling bacteria would adapt to high concentrations of environmental Sb by their efflux systems. To address these hypotheses, we designed a comprehensive experiment to characterize P-cycling bacterial communities and identified their underlying mechanisms of P cycling and Sb resistance using integrative approaches such as metagenome and amplicon sequencing, enzymatic assays and soil physicochemical measurements.

## Materials and methods

### Experimental design and sampling

**Fig. 1:**
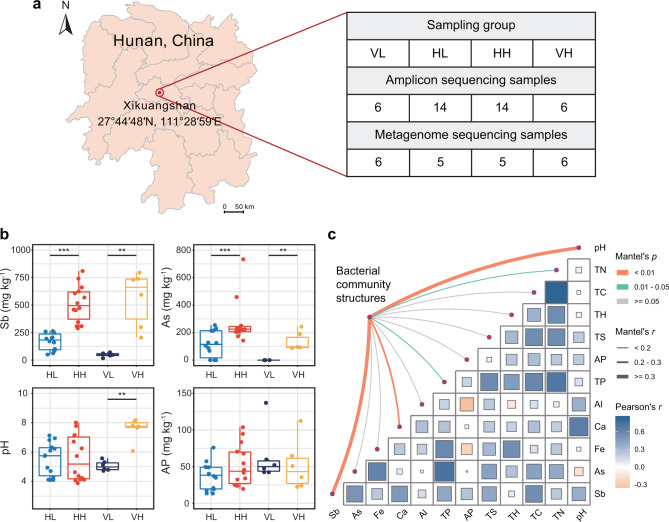
Sampling geographic location, soil properties and potential drivers of bacterial communities in Sb mining sites. **a** Collection of 40 soil samples from the Sb mining sites in Xikuangshan, Hunan, China (27°44’48’N, 111°28’59’E) with horizonal and vertical Sb contamination gradient. **b** Soil properties characterized in four types of contamination sites. **c** Correlations between the bacterial community structures (Bray-Curtis dissimilarity) and soil variables. Edge width and color corresponds to the Mantel’s *R* and *p* values, respectively. Pairwise Pearson’s correlations of soil variables are shown with a color gradient. HL horizontal low-contamination sites, HH horizontal high-contamination sites, VL vertical low-contamination sites, VH vertical high-contamination sites, Sb total antimony, As total arsenic, Fe total iron, Ca total calcium, Al total aluminum, TP total phosphorus, AP available phosphorus, TS total sulfur, TH total hydrogen, TC total carbon, TN total nitrogen. Significance levels were calculated by paired Student’s *t* test and denoted as **p < 0.01 and ***p < 0.001.

We selected the world’s largest Sb mining area (XKS, Hunan, China, 27°44′48′′N, 111°28′59′′E) as our study site (Fig. 1a). Mining activities have been carried out in XKS for over 100 years; thus, the soils of this and adjacent areas are considerably polluted [14]. We collected soil samples from 30 sites to cover different levels of Sb contamination from the central mining site and from areas at different distances from the site. From each site, soils from the 0–5 cm layer were collected as horizontal samples, which were divided into two groups (14 samples per group, Fig. 1a) according to Sb concentration (i.e., HH: horizontal high-contamination sites; HL: horizontal low-contamination sites). We also collected 12 vertical samples from high-contamination sites (VH) and low-contamination sites (VL) located at the closest and farthest places of this mining area. These samples were analyzed as complement of Sb effects with six depths (i.e., 0–5, 5–10, 10–20, 20–30, 30–40, 40–50 cm), as the underground roots of native plants in these sites were generally ~50 cm [39]. The six samples from each core were regarded as a group to address the comparison about high and low Sb-contaminated levels. Altogether, we collected 30 samples from the 0-5 cm layer, with an additional 10 samples from other layers (Fig. 1a). The soil samples were stored in a portable cooler (4 °C) and immediately transported to the laboratory. Then, each sample was divided into two subsamples: one was kept at 4 °C for physicochemical analysis, and the other was frozen at −80 °C for DNA extraction.

### Soil physicochemical and phosphatase activity analysis

The soil water content was determined after samples were air-dried at room temperature. The air-dried samples were ground and sifted through a 200-mesh sieve for subsequent analysis. Specifically, soil pH was measured using a pH meter (SevenCompact210, Mettler-Toledo, USA) with 2.0 g filtered soil mixed with 5.0 mL water (*g*/*v* = 1:2.5). Approximately 10 mg of filtered soil was used to determine the total carbon (TC), total nitrogen (TN), total sulfur (TS) and total hydrogen (TH) by an element analyzer (Vario EL cube, Germany). The total contents of Sb, As, aluminum (Al), calcium (Ca), iron (Fe) and P (TP) were extracted by full digestion of 0.5 g filtered soil using HF-HNO_3_-HClO_4_ and then determined by an inductively coupled plasma-optical emission spectrometer (ICP‒OES, Avio 500, Perkin Elmer, Singapore). Soil AP was determined according to previously described methods [40, 41]. Briefly, soil AP contents were first extracted using 0.5 *M* sodium bicarbonate and then analyzed using the ammonium molybdate-ascorbic acid blue method by measuring the absorbance at 700 nm with a microplate reader (Varioskan LUX, Thermo Scientific, USA). Soil ACP and AKP activities were determined as the generation rate of *p*-nitrophenol (PNP) using *p*-nitrophenyl phosphate as the substrate at pH values of 5.4 and 9.4, respectively. The ACP and AKP activities were expressed as μg PNP h^−1^ g^−1^ dry soil.

### Soil microbial community DNA extraction and high-throughput sequencing

For each sample, microbial community DNA was extracted from 10 g soil by a modified sodium dodecyl sulfate extraction method after freezing-grinding, followed by purification using a DNeasy PowerSoil Pro Kit (QIAGEN, Hilden, Germany) [42]. DNA quality was evaluated by a NanoDrop ND-2000 Spectrophotometer (Thermo Fisher Scientific, MA, USA) by the absorbance ratios of A260/280 (~1.8) and A260/230 (> 1.7). DNA was further quantified by a fluorescent method using the Qubit 4 Fluorometer (Thermo Scientific, USA).

For all 40 samples collected from both horizontal and vertical scales (Fig. 1a), the V4-V5 regions of the 16S rRNA gene were amplified using the universal PCR primers 515F (5′-GTGCCAGCMGCCGCGGTAA-3′) and 907R (5′-CCGTCAATTCCTTTGAGTTT-3′). After PCR amplification, purification and quantification, the 16S rRNA gene amplicons were sequenced on an NovaSeq platform (Illumina, 2 × 250 bp) at Novogene (Beijing, China). A total of 22 samples (12 from vertical soil cores, 10 from 0-5 cm, Fig. 1a) were selected for shotgun metagenome sequencing. Metagenome sequencing library construction was performed using 1 μg of high-quality DNA by the VAHTS Universal DNA Library Prep Kit for Illumina V3 ND670 (Vazyme Biotechnology, China), and the quality of the constructed libraries was determined by LabChip GX Touch HT (PerkinElmer, USA). The prepared metagenome libraries were sequenced on our NextSeq 550 platform (Illumina, 2 × 150 bp).

### 16S rRNA gene amplicon data analysis

The 16S rRNA gene amplicon data were analyzed with the QIIME 2 pipeline (v2021.8) [43]. Briefly, after removing barcodes and primers, raw data were quality-filtered and denoised with DADA2 [44]. The amplicon sequence variants (ASVs) of each sample were obtained and rarefied with the minimum frequency (i.e., 50,566) for the following analysis. The representative sequences of ASVs were assigned to phylogenetic taxonomy based on the SILVA database release 138 [45] by the feature-classifier module after training the Naive Bayes classifier based on the primer sequences.

### Metagenome assembly, binning and annotation

In total, 586.98 Gb raw metagenome sequences (≥ 22.96 Gb per sample) were generated from the 22 sequenced samples. Each sample was trimmed, assembled and binned individually according to the metaWRAP pipeline (v1.2.1) [46]. The raw reads were first trimmed with the Read_qc module to remove low-quality reads and then assembled into contigs using MEGAHIT (v1.1.3) [47] with various *k*-mer sizes (ranging from 21 to 141). Only contigs ≥ 500 bp were kept for subsequent analysis.

The open reading frames (ORFs) of all the assembled contigs were predicted using Prodigal (v2.6.3) [48]. Then, the putative protein-coding sequences were searched against the KEGG database (release 94.2) and PCycDB using DIAMOND BLASTx (v0.9.24) [49] with an *e*-value ≤ 10^−5^. Detailed information on Sb-resistance and P-cycling genes is summarized (Table S1). PCycDB is a comprehensive and accurate database for analyzing P-cycling genes and microorganisms [50]. To reduce false-positives, the results searched against PCycDB were filtered with an identity ≥ 30.0% and hit length ≥ 25 amino acids [50]. Then, gene abundances were normalized into reads per kilobase per million mapped reads (RPKM) using CoverM (v0.6.1) (https://github.com/wwood/CoverM). To perform taxonomic annotation, the ORFs of each gene were extracted and searched against the NCBI-nr database (release June 12th, 2020) using DIAMOND BLASTx with an *e*-value ≤ 10^−5^ and finally parsed using MEGAN6 (v6.21.16) [51].

The assembled contigs from each sample were individually binned using Metabat2 (v2.12.1) [52] and MaxBin2 (v2.2.7) [53]. The original bins were consolidated and improved with Bin_refinement and Reassemble_bins modules in metaWRAP. The quality of the bins was evaluated with CheckM (v1.0.12) [54]. Bins with estimated genome completeness >90% and contamination < 5% were kept as high-quality metagenome-assembled genomes (MAGs), and we finally obtained 106 high-quality bacterial MAGs for subsequent analysis. The abundance of each MAG across samples was calculated as genome copies per million reads (GPM) using Salmon (v0.13.1) [55]. Functional genes were predicted for individual MAGs using Prodigal and assigned to the KEGG database and PCycDB with DIAMOND BLASTx (*e*-value ≤ 10^−5^). To ensure more precise and reliable results, we filtered the annotations of Sb-resistance genes using a criterion of identity ≥40.0%, while P-cycling genes were filtered with a criterion of identity ≥ 30.0% and hit length ≥ 25 amino acids. In addition, potential metabolic functional genes from three representative MAGs were predicted and annotated for the primary biogeochemical cycles using METABOLIC (v4.0) [56]. All MAGs were classified taxonomically with GTDB-Tk (v2.1.0) [57] (GTDB release 207) [58] using the classify_wf module.

### Phylogenetic analysis and potential horizontal gene transfer identification

A phylogenetic tree of all 106 high-quality bacterial MAGs was constructed using the GTDB-Tk infer module based on a set of 120 bacteria-specific marker genes from GTDB (release 207). To construct a protein phylogenetic tree, the Acr3 and ArsB protein sequences identified from MAGs and reference protein sequences retrieved from Chen et al. [59] and NCBI were first aligned using MAFFT (v7.490) [60] and excised using TrimAL (v1.4.15) [61] with the -automated1 option. A maximum likelihood phylogenetic tree was then generated by IQ-TREE (v2.2.0-beta) using 1000 bootstraps [62], with the parameters set to -m MFP -B 1000 --bnni -T AUTO. The MAG-, Acr3 protein- and ArsB protein- based phylogenetic trees were visualized in iTOL (v6) (https://itol.embl.de) [63].

ORFs from MAGs were assigned against the custom mobile genetic element (MGE) database [64] using BLASTn (v2.12.0+) [65] at a criterion of query coverage ≥ 40.0%, identity ≥ 25.0%, and *e*-value ≤ 10^−5^ [66] to exhibit probable transmission of genes between microbial taxa. In addition, to further confirm the potential spread of the *acr3* gene, horizontal gene transfer (HGT) among MAGs at the phylum level was explored by MetaCHIP (v1.10.10) [67] with default parameters using best-match (BLASTn) and phylogenetic approaches.

### Statistical analysis

All statistical analyses were performed in R (v4.2.1). Pearson’s correlation analysis was performed to assess the correlations between two parameters. A heatmap was constructed to illustrate the abundance of P-cycling and Sb-resistance genes in metagenome sequencing data using the “pheatmap” package. To compare the microbial diversity, the Shannon and Simpson indices of bacterial communities, as well as those for *acr3*- and *gcd*-harboring communities, were calculated using the “vegan” package. Redundancy analysis (RDA) was used to determine the influence of environmental factors on the microbial community using the “vegan” package [14]. Random forest (RF) analysis was performed using the “randomForest” package to identify which microbial P-cycling genes mainly influenced soil AP, and the significances of each predictor and model were assessed with 1000 permutations using the “rfPermute” and “A3” packages, respectively [68]. A partial least squares-path model (PLS-PM) was constructed to explore the effects of both biotic and abiotic factors (e.g., P-cycling genes, heavy metals, pH and TP) on the soil AP using the “plsmp” package [69].

## Results

### Total Sb and pH impacted soil phosphatase activities and bacterial communities in the Sb mining area

Based on soil physicochemical parameters in the XKS, the investigated samples could be separated into low- and high-contamination groups in both horizontal and vertical profiles. The total Sb and As were significantly higher (*p* < 0.01) in high-contamination sites (HH and VH) than in low-contamination sites (HL and VL; Fig. 1b). All the other investigated metals (i.e., Ca, Al and Fe) and major biogenic elements (i.e., TP, TC, TN, TS and TH) were also higher in high-contamination sites than in low-contamination sites (Fig. S1). The soil was acidic in VL, HL and HH (pH of 5.47 ± 1.20), whereas that of VH was alkaline (pH of 7.59 ± 0.70). The soil average AP was ~50 mg kg^−1^, with a range of 10 ~ 75 mg kg^−1^ (Fig. 1b), which was much lower than that of normal forest and cropland ecosystems [70, 71]. Soil AKP activities were higher in high-contamination sites than in low-contamination sites, whereas ACP activities showed the opposite trend (Supplementary Fig. S2a). These variances were consistent with soil pH. Moreover, the two vertical cores showed steep decreases from surface to deep soils for both geochemical parameters and phosphatase activities (Supplementary Figs. S2b and S3).

Based on the 16S rRNA gene sequencing data, most detected bacterial taxa were classified as Actinobacteriota, Proteobacteria, Chloroflexota and Acidobacteriota (Supplementary Fig. S4), collectively accounting for over 75% of the detected taxa. At the genus level, *Acidothermus* and *Bacillus* were the most abundant genera, accounting for 4.7% and 2.8% across all sites, respectively (Supplementary Fig. S5). The bacterial community alpha diversity represented by the Shannon and Simpson indices was higher in HL and VH than in HH and VL, respectively (Supplementary Fig. S6a). With increasing soil depth, bacterial community diversity showed a decreasing trend in VL and a slightly increasing trend in VH (Supplementary Fig. S6b). The structure of the bacterial communities exhibited strong correlations with pH and total Sb (*p* < 0.01; Fig. 1c). RDA also revealed that total Sb and pH were the key and covarying factors in explaining the variation in bacterial communities in both horizontal and vertical profiles (Supplementary Fig. S7). These results indicated that total Sb and pH were the major factors that impacted soil phosphatase activities and bacterial community diversity and structure.

### Pi-solubilizing microbiomes enhanced soil P bioavailability

**Fig. 2:**
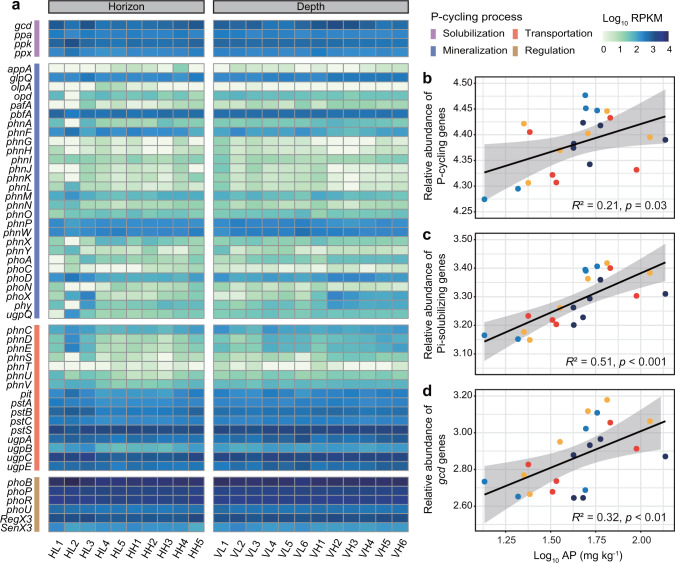
Microbial P-cycling potential as depicted by metagenome sequencing of representative samples from Sb mining sites. **a** Relative abundance of microbial P-cycling genes characterized at each site. Pearson’s correlation between the relative abundance of (**b**) P-cycling genes, (**c**) Pi-solubilizing genes, (**d**) *gcd* genes and soil available phosphorus (AP), respectively. The relative abundance of related genes was calculated as Log_10_ RPKM (reads per kilobase per million mapped reads).

To explore the contribution of microbial P-cycling genes to soil AP, we obtained 586.98 Gb metagenome sequencing data and identified a total of ~17 million ORFs, and genes involved in P turnover and Sb resistance were found (Fig. 2a and Supplementary Fig. S8). There was a strong positive relationship between the relative abundance of P-cycling genes and soil AP (*R*^2^ = 0.21, *p* < 0.05; Fig. 2b). Specifically, the relative abundance of Pi-solubilizing genes was significantly positively correlated with soil AP (*R*^2^ = 0.51, *p* < 0.001; Fig. 2c). The relative abundance of *gcd* genes was predominantly high (Fig. 2a) and showed a significant positive correlation with soil AP (*R*^2^ = 0.32, *p* < 0.01; Fig. 2d). We also observed a strong positive correlation between P-regulating genes and soil AP (*R*^2^ = 0.23, *p* < 0.05; Supplementary Fig. S9a). Although organic P-mineralizing genes such as those encoding phosphodiesterase (*glpQ*), transaminase (*pbfA* and *phnW*), C-P lyase subunit (*phnF* and *phnP*) and alkaline phosphatase (*phoD*), as well as P-transporting genes such as those encoding Pi transporters (*pit* and *pstSCAB*), showed relatively high abundances (Fig. 2a), the relative abundances of these genes were not significantly correlated with soil AP (Supplementary Fig. S9b, c).

**Fig. 3:**
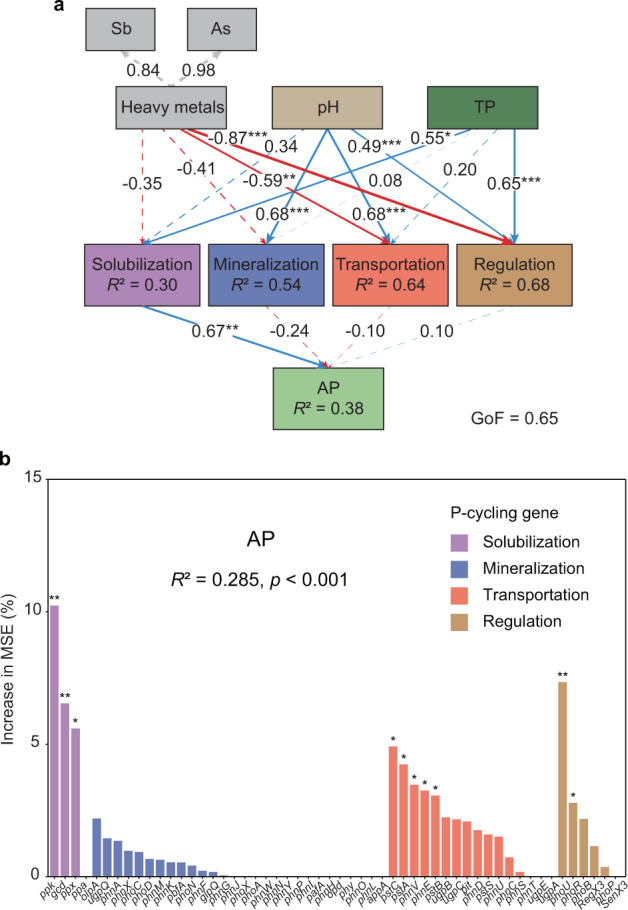
Linkages between genes responsible for soil microbial P cycling and P bioavailability in Sb mining sites. **a** Partial least squares-path model analysis showing the environmental and microbial effects on soil P bioavailability. Path coefficients, significance levels and coefficients of determination (*R*^2^) were calculated after 1000 bootstraps. Blue and red arrows represent positive and negative effects, respectively. **b** Random forest analysis showing the contribution of microbial P-cycling genes to soil P bioavailability. The variance interpretation rate of the whole model (*R*^2^), the significance levels of the whole model and each variable were calculated after 1000 bootstraps. GoF goodness of fit, TP total phosphorus, AP available phosphorus, MSE mean squared error. Significance levels were denoted as *p < 0.05, **p < 0.01 and ***p < 0.001.

Further statistical analysis showed that PLS-PM could explain 37.9% of the total variation in soil P bioavailability (Fig. 3a). Genes involved in Pi solubilization had a strong positive effect on soil AP (path coefficient = 0.671, *p* < 0.01; Fig. 3a), which was also verified by correlation analysis (Fig. 2c). Furthermore, heavy metals, pH and soil TP also impacted soil AP by influencing microbial P-cycling genes (Fig. 3a). Collectively, the Pi-solubilizing genes were the factors that were most positively correlated with soil P bioavailability, followed by soil TP, while heavy metals negatively affected soil AP (Supplementary Fig. S10). RF analysis was used to further evaluate the key P-cycling genes to predict soil P bioavailability (*R*^2^ = 0.671, *p* < 0.001; Fig. 3b). The *gcd* gene was one of the most important factors determining soil AP and the only significant gene encoding enzymes responsible for releasing free extracellular P (*p* < 0.01). These results suggested that microbially driven Pi solubilization, governed by *gcd*-harboring microorganisms, was the dominant P-cycling process in Sb-contaminated soils.

### Different patterns in bacteria containing the Pi-solubilizing gene *gcd* and Sb-efflux gene *acr3*

**Fig. 4:**
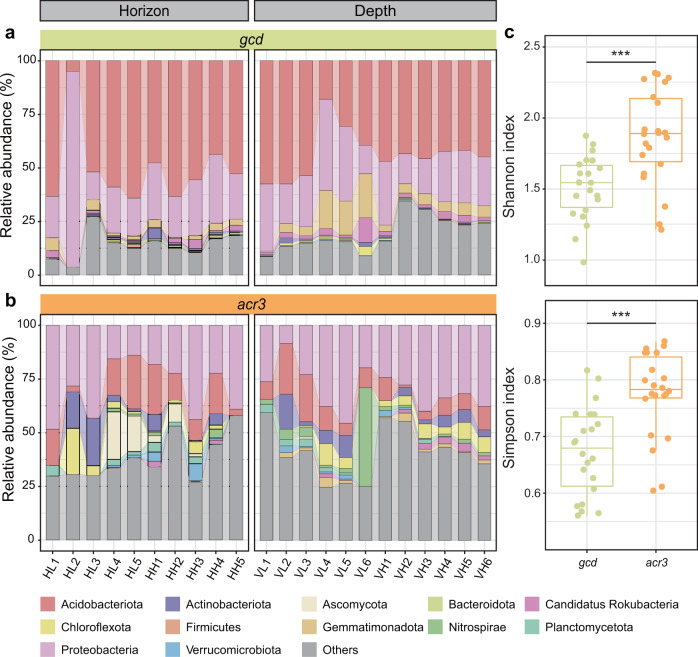
Microbial composition and diversity analysis reveals Pi-solubilizing and Sb-efflux functional groups in Sb mining sites. Microbial composition of functional groups as depicted by the Pi-solubilizing gene (*gcd*, **a**) and Sb-efflux gene (*acr3*, **b**), respectively. **c** Comparison of the alpha diversity of microbial communities. The taxonomic assignments were based on the open reading frames of all the assembled contigs against the NCBI-nr database. The relative abundance of related contigs was calculated as reads per kilobase per million mapped reads (RPKM). Significance levels were calculated by paired Student’s *t* test and denoted as *p < 0.05, **p < 0.01 and ***p < 0.001.

Since the *gcd* gene is critical in P cycling, taxonomic classification of *gcd*-harboring bacteria was further performed to understand bacterial distribution across different Sb gradients (Fig. 4a). More than 50% of the identified *gcd*-harboring bacteria were assigned to Acidobacteriota, followed by Proteobacteria and Gemmatimonadota. The relative abundance of *gcd*-harboring Gemmatimonadota increased across the soil depth. Additionally, we characterized the composition of bacteria harboring the *acr3* gene (Fig. 4b) to evaluate bacterial Sb-resistance potential because the gene is ubiquitous among living microorganisms [59]. Compared to *gcd*-harboring bacteria, *acr3*-harboring bacteria showed significantly higher diversity (*p* < 0.001; Fig. 4c). In fact, nearly half of *acr3*-harboring bacteria were affiliated with the top 10 phyla, such as Proteobacteria, Acidobacteriota and Actinobacteriota, which was consistent with amplicon sequencing results (Supplementary Fig. S4). These results indicated that the Pi-solubilizing microorganisms mainly belonged to Acidobacteriota, and Sb expulsion potential was widespread among microbiomes in the Sb mining area.

### Functional annotation of P-cycling and Sb-resistance genes of MAGs in the Sb mining area

**Fig. 5:**
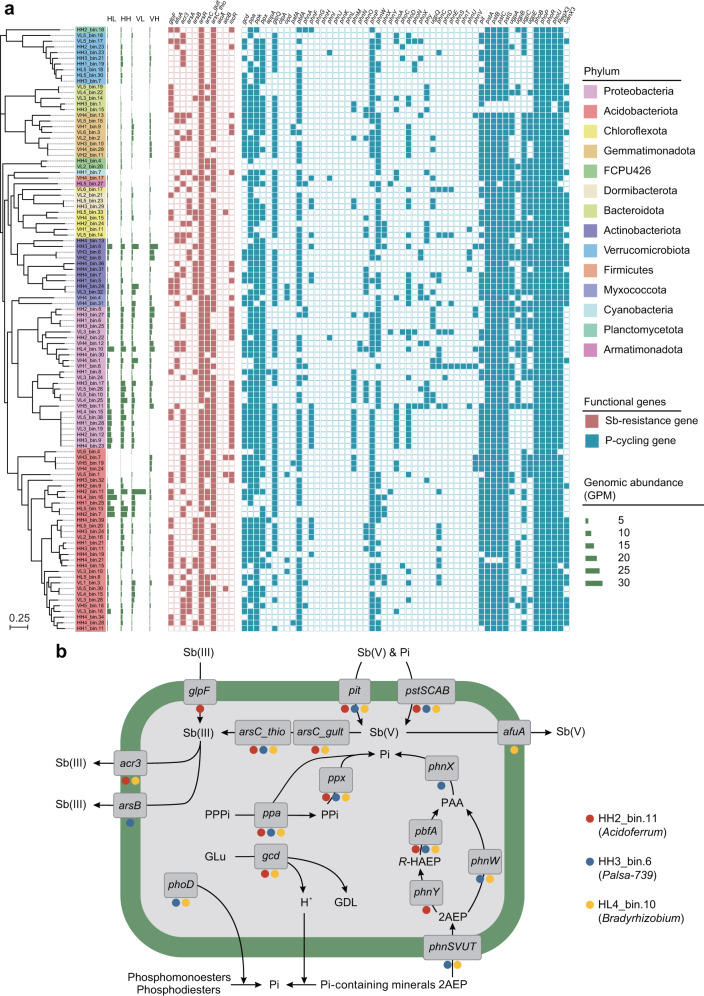
Phylogenetic distribution of high-quality bacterial MAGs and related genes responsible for P turnover and Sb resistance. **a** Phylogenetic and functional characterization of 106 high-quality bacterial MAGs recovered from mining soils. The construction of the maximum likelihood tree was based on a concatenated alignment of 120 marker genes from GTDB-Tk. The bar plot showed the relative abundance of each MAG at the four types of contaminated sites. The presence (colored) and absence (blank) of protein-encoding genes are represented by the heatmap. The 0.25 scale bar indicates the tree scale. **b** Mechanisms of P turnover and Sb resistance as indicated by three representative MAGs. Circles with different colors represent different MAGs. Sb(III) antimonite; Sb(V), antimonate, Pi inorganic phosphate, PPi inorganic diphosphate, PPPi inorganic triphosphate, GLu glucose, GDL glucono-1,5-lactone, 2AEP, 2-aminoethylphosphonate, *R*-HAEP *R*‑1-Hydroxy-2-aminoethylphosphonate, PAA phosphonoacetaldehyde.

We assembled and binned 106 high-quality bacterial MAGs (completeness > 90% and contamination < 5%) spanning 14 phyla (Supplementary Table S2). Consistent with the community composition depicted by 16S rRNA gene sequencing (Supplementary Fig. S4), over half of these MAGs were affiliated with the dominant phyla Proteobacteria, Acidobacteriota and Actinobacteriota (Fig. 5a). Functional annotation confirmed their versatile P-cycling potential and Sb-resistance strategies (Fig. 5a). Specifically, most MAGs harbored functional genes involved in Pi solubilization (60.4%), P uptake (99.1%), Sb reduction (97.2%) and Sb efflux (78.3%). 98.4% of *gcd*-harboring MAGs carried genes encoding Pi transport systems (i.e., *pit* and *pstSCAB*), and nearly half of the *gcd*-harboring MAGs also carried the *acr3* gene. Out of the 106 retrieved MAGs, 22 *arsB* genes were identified in 20 MAGs, while 58 *acr3* genes were detected in 45 MAGs, indicating that *acr3* could play a more prominent and widespread role in bacterial Sb efflux.

Three representative MAGs (i.e., HH2_bin.11, HH3_bin.6 and HL4_bin.10) that had the highest average relative abundances in all samples were used to further examine microbial P cycling and Sb resistance (Fig. 5b and Supplementary Table S3). Both HH2_bin.11 (*Acidoferrum*, Acidobacteriota) and HL4_bin.10 (*Bradyrhizobium*, Proteobacteria) harbored the *gcd* gene encoding glucose dehydrogenase, which was found to play an important role in mineral-sorbed Pi solubilization [72]. Additionally, HH3_bin.6 (*Palsa-739*, Actinobacteriota) and HL4_bin.10 harbored the *phoD* gene encoding extracellular alkaline phosphatases for organic P hydrolysis, while HH3_bin.6 had all the genes encoding the complete 2-aminoethylphosphonate (2AEP) utilization pathway. Additionally, these three MAGs all contained genes (*ppa* and *ppx*) involved in oxidative phosphorylation, as well as genes (*pit* and *pstSCAB*) associated with Pi and antimonate uptake. The three MAGs also carried the *arsC* gene encoding arsenate reductase for reducing antimonate to antimonite and the *acr3* or *arsB* gene encoding antimonite extrusion channels. These results exhibited the versatile P-cycling and Sb-resistance potential of bacteria in the Sb mining area.

### Phylogeny of the Sb efflux pump Acr3 and its potential horizontal gene transfer

**Fig. 6:**
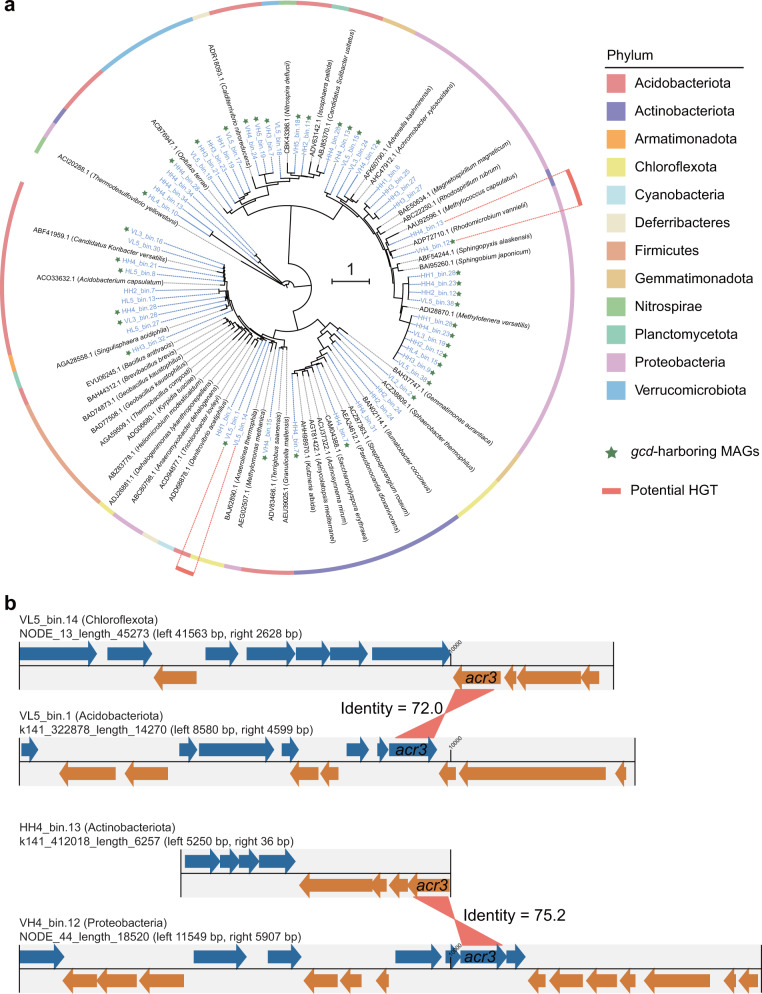
Phylogenetic distribution and potential horizontal gene transfer (HGT) events of Sb efflux pump Acr3. **a** The maximum-likelihood phylogenetic tree after 1000 bootstraps showing Acr3 sequences from the recovered MAGs (blue) alongside representative sequences from reference genomes (gray). The 1 scale bar indicates the tree scale. **b** Identification of potential HGT for the *acr3* gene among the recovered MAGs at the phylum level.

To investigate the distribution and dissemination of the Sb efflux pump in P-cycling bacteria, we constructed a maximum-likelihood tree of Sb efflux pumps Acr3 and ArsB using sequences from MAGs and reference sequences [59]. A total of 20 MAGs harbored 23 genes encoding ArsB proteins (Supplementary Fig. S11), and a total of 45 MAGs harbored 58 genes encoding Acr3 proteins (Fig. 6a). Among them, 28 MAGs harbored the *gcd* gene (Fig. 6a). Therefore, we selected *acr3* as our representative Sb efflux gene to investigate bacterial efflux potential due to its dominant role among the MAGs retrieved in this study. Consistent with the taxonomic composition of *acr3*-harboring bacteria, *acr3*-harboring MAGs were mainly affiliated with Acidobacteriota, Proteobacteria and Actinobacteriota (Fig. 6a). The Acr3 protein sequences from Acidobacteriota, the major members of *gcd*-harboring bacteria, were mainly clustered into three subgroups and merged with other phyla, but Acidobacteriota genomes clustered into one group in the MAG-based phylogenetic tree (Supplementary Fig. S12), suggesting that some Acidobacteriota members might acquire *acr3* via horizontal gene transfer (HGT). Similar comparisons suggested that potential HGT for *acr3* could occur among some members of Proteobacteria, Actinobacteriota and Chloroflexota (Supplementary Fig. S12).

To further explore such potential, we characterized the genetic organization of *acr3*, other Sb-resistance genes, and P-cycling genes on the same contig within a MAG. The syntenies of *acr3*-*arsC* and *acr3*-*pstSCAB*/*pit* were frequently detected among different MAGs (Supplementary Fig. S13), indicating the co-occurrence of HGTs. Furthermore, we identified mobile genetic elements (MGEs) and potential HGTs, and transposases constituted the most common MGE class, followed by insertion elements. A total of 22 genes encoding MGEs belonged to four classes and were distributed among 11 MAGs, of which 72.7% carried *acr3* (Supplementary Table S4). At the phylum level, potential HGTs involving *acr3* might occur between VL5_bin.14 (Chloroflexota) and VL5_bin.1 (Acidobacteriota) and between HH4_bin.13 (Actinobacteriota) and VH4_bin.12 (Proteobacteria) (Fig. 6b). Notably, VH4_bin.12, as the recipient of *acr3*, contained the gene encoding insertion_element_IS91 (Supplementary Table S4). Two MAGs possibly acquiring *acr3* also harbored the *gcd* gene, suggesting that Pi-solubilizing bacteria might gain Sb-resistance genes through HGT in the Sb mining area.

## Discussion

Mining areas with high levels of heavy metal contamination are usually characterized by low P availability [73] and poor biogeochemical P cycling [38]. Given the crucial roles of microbially mediated P cycling in natural environments, understanding how microorganisms adapt to heavy metal contamination and drive P cycling is especially important for the future management and remediation of mining lands [11, 74]. We found that *gcd*-harboring bacteria were the key drivers of Pi solubilization, the most important determinant of soil P bioavailability. Prevalent *pit* or *pstSCAB* genes enabled *gcd*-harboring bacteria to scavenge AP and absorb antimonate into cells. In addition, the *acr3* gene, which is responsible for intracellular Sb efflux was widespread, and *gcd*-harboring bacteria could potentially acquire *acr3* via HGT. This study provides potential mechanisms of microbial P cycling and developed microbial Sb-resistance strategies in Sb-contaminated environments.

P is indispensable in maintaining both the compositional and functional stability of soil microbial communities, but AP is permanently limited in mining ecosystems [4]. Microorganisms play vital roles in regulating AP in soils by carrying out versatile processes for P bioconversion [75]. Soil bacteria harboring genes for Pi solubilization and organic P mineralization can effectively release Pi from inorganic and organic complexes [23]. Previous studies indicated that Pi-solubilizing bacteria with the *gcd* gene predominantly enhanced P cycling and P bioavailability in postmining [11] and agricultural soils [30]. Here, we detected a strong positive correlation between the relative abundance of P-cycling genes and soil AP, suggesting that microbial P cycling played essential roles in the accumulation of soil AP. Specifically, microbial Pi solubilization, largely correlated with *gcd*-harboring bacteria, was a dominant process enhancing P bioavailability in mining soils. We found that nearly two-thirds of the reconstructed high-quality MAGs in this study carried *gcd*, and the relative abundance of *gcd* genes showed a strong positive correlation with soil AP, suggesting that gluconic acid production to solubilize recalcitrant Pi was a dominant Pi-releasing strategy [29]. Furthermore, most *gcd*-harboring bacteria were affiliated with Acidobacteriota, which was found to be a new and primary Pi-solubilizing taxon in recent studies [72, 75]. For example, nearly one-third of *gcd*-harboring MAGs in postmining lands were found to be Acidobacteriota [11]. Acidobacteriota were found to be the principal members of both bacterial and *gcd*-harboring communities from horizontal and vertical profiles, indicating their influential roles in Pi solubilization in Sb mining soils. In contrast, there was no significant relationship between organic P-mineralizing genes and soil AP. Such inconsistencies with results from agricultural ecosystems [76, 77] might occur because croplands are frequently fertilized [78], which results in large amounts of organic P accumulation [71]. In contrast, soil P in mining areas are mainly immobilized in Pi-containing rocks [74] and can be released through microbial Pi solubilization [79]. Additionally, genes encoding extracellular phosphatase and phytase (e.g., *phoD*, *phoN* and *phy*) were detected in contigs and MAGs, indicating bacterial organic P-mineralizing potential in the Sb mining area. Specifically, the representative HH3_bin.6 showed a complete pathway for the uptake and degradation of 2AEP, one of the most common biogenic phosphonates [80, 81]. In addition, the regulatory genes that responded to P starvation showed relatively high abundances, suggesting that the related microorganisms intended to obtain additional P under P-deficient conditions [82].

Given the effects of severe Sb contamination, P-cycling bacteria in such mining soils would require Sb resistance to survive and perform their functions [33]. In this study, Sb-resistance genes (e.g., *aioA*, *arsC*, *arsAB*, *acr3*) were identified in most MAGs that had P-cycling potential, suggesting that P-cycling bacteria can develop diverse Sb-resistance strategies, including oxidation, reduction and efflux. The widespread presence of Pi transport systems in the MAGs indicated the importance of nutrient uptake capabilities in supporting bacterial growth in mining ecosystems [18]. However, due to the similar chemical properties of P and Sb, bacteria residing in Sb-contaminated environments would absorb Pi and antimonate from their surroundings into cells via the Pi transport systems encoded by *pit* and *pstSCAB* [34, 35], which have also been reported as arsenate uptake systems [83]. Therefore, bacteria might accumulate antimonate in cells during Pi assimilation from the environment. The arsenite efflux pump encoded by *acr3* often exists in microorganisms [59], which also functions as an antimonite efflux transporter to perform microbial Sb detoxification [84, 85]. In this study, *acr3* genes were observed in 42.5% of retrieved MAGs, indicating their dominant role in bacterial Sb efflux in contaminated sites. We also found that *acr3*-harboring bacteria showed much higher biodiversity than *gcd*-harboring bacteria, suggesting that Sb efflux was a universal mechanism for microbial Sb resistance [86]. We also found that 98.4% of *gcd*-harboring MAGs carried *pit* or *pstSCAB* encoding Pi transport systems, and nearly half of them carried *acr3*, suggesting that Pi-solubilizing bacteria in the mining area might extrude cellular Sb through the Acr3 efflux pump to adapt to Sb contaminated conditions.

HGT is a common way for resistance genes to spread among microorganisms, and it further promotes microbial adaptation to extreme environments, such as acid mine drainage waters [87] and hypersaline habitats [88]. It has been suggested that HGT mediated by MGEs contributes to the dissemination of resistance genes [66]. A previous study found that Sb-resistant strains isolated from a hydrothermal vent carried *acr3* and plasmids, and some strains showed putative HGT of *acr3* [89]. Thus, P-cycling bacteria in Sb-contaminated environments might gain *acr3* via HGT. In this study, the different patterns of MAGs and Acr3 protein phylogenetic trees provided evidence that HGT was essential for the acquisition of *acr3* in Acidobacteriota, the most frequently detected *gcd*-harboring taxa. The identification of MGEs from the *acr3*-harboring MAGs suggested that transposase was a central factor in *acr3* dissemination, consistent with a previous study showing that transposase could promote the spread of metal resistance genes in a heavy metal-contaminated area [90]. Furthermore, two potential HGT processes with *acr3* were identified among MAGs at the phylum level. More importantly, VH4_bin.12, also a possible *acr3* recipient, carried the *IS91* gene encoding an insertion element (MGE). Two MAGs that potentially acquired *acr3* through HGT were found to harbor the *gcd* gene and other P-cycling genes, suggesting that selective pressure from heavy metal contamination enhanced the efflux traits of P-cycling bacteria through HGT [91]. Meanwhile, the detection of syntenies of *acr3*-*arsC* and *acr3*-*pstSCAB*/*pit* among different MAGs indicated the co-occurrence of HGTs with Sb-efflux, reducing and P-transporting genes. These findings were consistent with a previous study, showing that Sb-resistance genes and P-transporting genes were often co-existed in bacterial genomic islands [92]. We acknowledge that such identified adaptive HGTs need to be validated through metatranscriptome and laboratory experiments [93], which will help us to further elucidate the microbial P-cycling and Sb-resistance mechanisms in mining soils.

Multiple studies have indicated that toxicity associated with elevated Sb concentrations could greatly affect the composition, distribution and functional traits of microbial communities in mining areas [94, 95]. In turn, P-cycling bacteria could develop related resistance strategies to survive in mining environments that have severe contamination [96]. For instance, *Bacillus*, a major genus in highly contaminated sites, has been extensively characterized as a phosphate-solubilizing bacterium that has the ability to solubilize a broad spectrum of inorganic and organic phosphates [75], indicating its crucial role in P cycling in Sb mining areas. Moreover, we detected Sb-resistance genes involved in oxidation, reduction and efflux in all reconstructed MAGs. Soil pH could also impact both the toxicity and mobility of Sb by changing its speciation, further affecting the microbiome [97]. We found that pH and Sb were key and covarying factors affecting the diversity, structure and P-cycling traits of bacterial communities in the contaminated soils. Additionally, soil P bioavailability was improved in alkaline conditions because higher abundance of genes encoding alkaline phosphatases and alkaline phosphatase activities were present [98]. We also found that severe heavy metal contamination inhibited microbial P turnover, further decreasing soil AP.

**Fig. 7:**
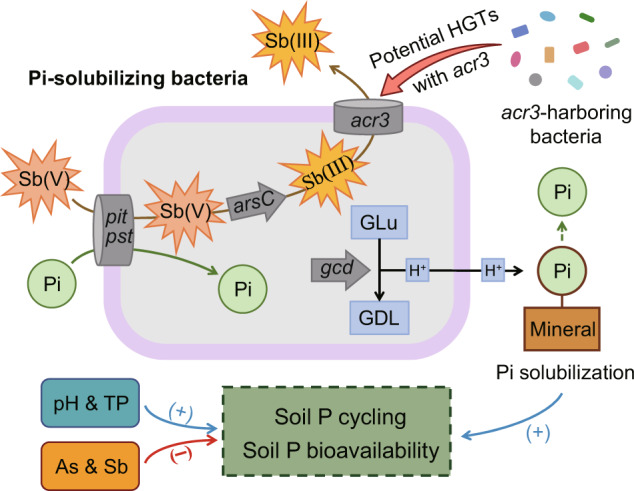
A conception model summarizing Sb efflux and the underlying mechanism by which phosphate-solubilizing bacteria affect P cycling in Sb mining soils. Sb total antimony, As total arsenic, Sb(III) antimonite, Sb(V) antimonate, TP total phosphorus, Pi inorganic phosphate, GLu glucose, GDL glucono-1,5-lactone, HGT horizontal gene transfer.

In summary, this study revealed the possible molecular mechanisms of microbial P cycling and Sb resistance in Sb-contaminated sites (Fig. 7). We found that the AP in contaminated soils was mainly determined by microbial Pi solubilization, largely correlated with *gcd*-harboring bacteria. Severe Sb contamination inhibited microbial P-cycling traits, and P-cycling microorganisms exhibited diverse Sb-resistance strategies. Moreover, the *acr3* gene responsible for Sb efflux tended to transfer horizontally to Pi-solubilizing bacteria. These findings provide evidence for the potential role of P-solubilizing bacteria in Sb detoxification, and this study also expands our understanding of the diversity and possible genetic mechanisms of microbial P cycling and Sb resistance in mining soils. Since heavy metal pollution is a significant environmental issue that adversely impacts soil health and plant growth, this study has broader ecological implications for utilizing P-cycling microorganisms to facilitate effective bioremediation of such contaminated soil environments.

## Supplementary information

Supplementary figures

Supplementary table S1

Supplementary table S2

Supplementary table S3

Supplementary table S4

## Data Availability

The 16S rRNA gene amplicon and metagenome sequencing raw data were deposited in the NCBI BioProject database under the accession numbers PRJNA883072 and PRJNA886109, respectively.
