# Supplementary material for: Antimony efflux underpins phosphorus cycling and resistance of phosphate-solubilizing bacteria in mining soils
Source: ISME J. 2023 Jun 3;17(8):1278–89. doi: 10.1038/s41396-023-01445-6 (PMC10356851; doi:10.1038/s41396-023-01445-6)
Supplement: Supplementary file 1 — Supplementary figures [file 41396_2023_1445_MOESM1_ESM.docx]

**Supplementary Figures**

**Antimony efflux underpins the phosphorus cycling and resistance of phosphate solubilizing bacteria in mining soils**

Shengwei Liu^1^, Jiaxiong Zeng^1^, Huang Yu^1^, Cheng Wang^1^, Yunfeng Yang^2^, Jianjun Wang^3^, Zhili He^1^, Qingyun Yan^1,*^

^1^ Environmental Microbiomics Research Center, School of Environmental Science and Engineering, Southern Marine Science and Engineering Guangdong Laboratory (Zhuhai), State Key Laboratory for Biocontrol, Sun Yat-sen University, Guangzhou 510006, China

^2^ State Key Joint Laboratory of Environment Simulation and Pollution Control, School of Environment, Tsinghua University, Beijing 100084, China

^3^ State Key Laboratory of Lake Science and Environment, Nanjing Institute of Geography and Limnology, Chinese Academy of Sciences, Nanjing 210008, China

^*^ Corresponding author.

E-mail addresses: [yanqingyun@sml-zhuhai.cn](mailto:yanqingyun@sml-zhuhai.cn) (Qingyun Yan)


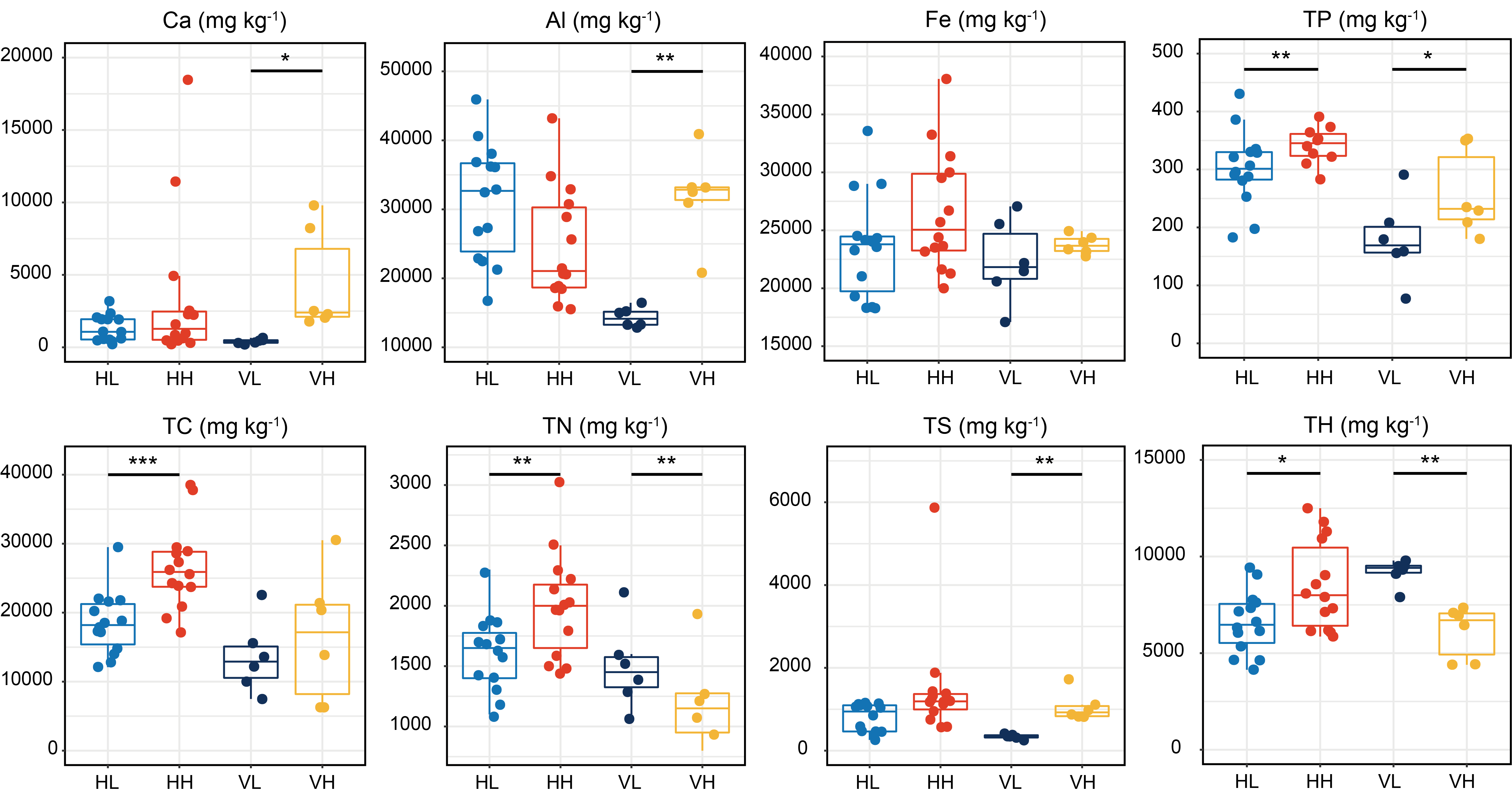


**Fig. S1 Comparison of the measured environmental factors for soil samples collected from four types of contaminated sites.** HL, horizontal low-contamination sites; HH, horizontal high-contamination sites; VL, vertical low-contamination sites; VH, vertical high-contamination sites; Ca, total calcium; Al, total aluminum; Fe, total iron; TP, total phosphorus; TC, total carbon; TN, total nitrogen; TS, total sulfur; TH, total hydrogen. Significance levels were calculated by paired Student's *t*-test and denoted as **p* < 0.05 and ***p* < 0.01.


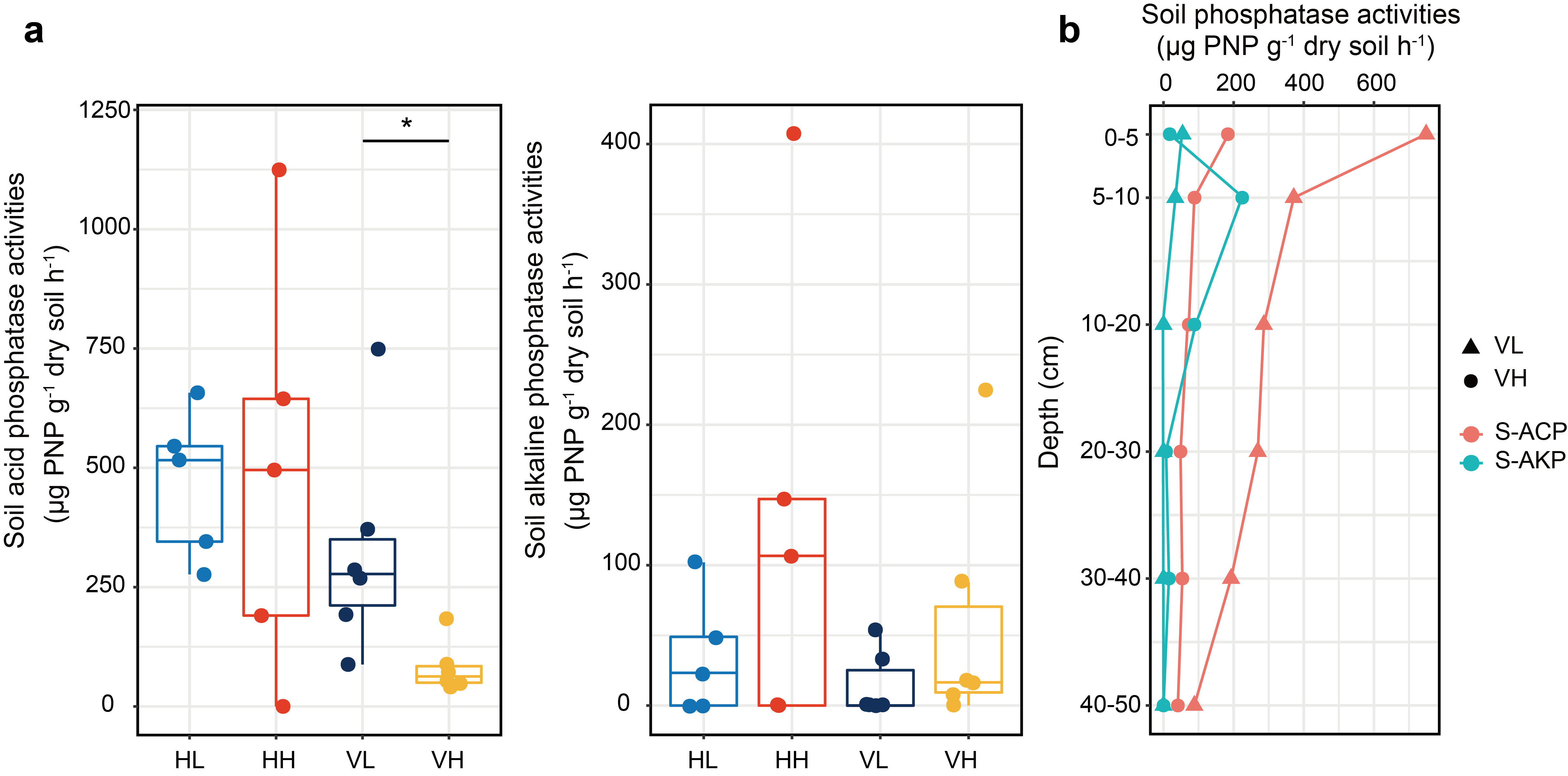


**Fig. S2 Comparison of the soil acid and alkaline phosphatase activities.** (a) Comparison among four groups, (b) Comparison among two vertical soil cores. HL, horizontal low-contamination sites; HH, horizontal high-contamination sites; VL, vertical low-contamination sites; VH, vertical high-contamination sites. Significance levels were calculated by paired Student's *t*-test and denoted as **p* < 0.05.


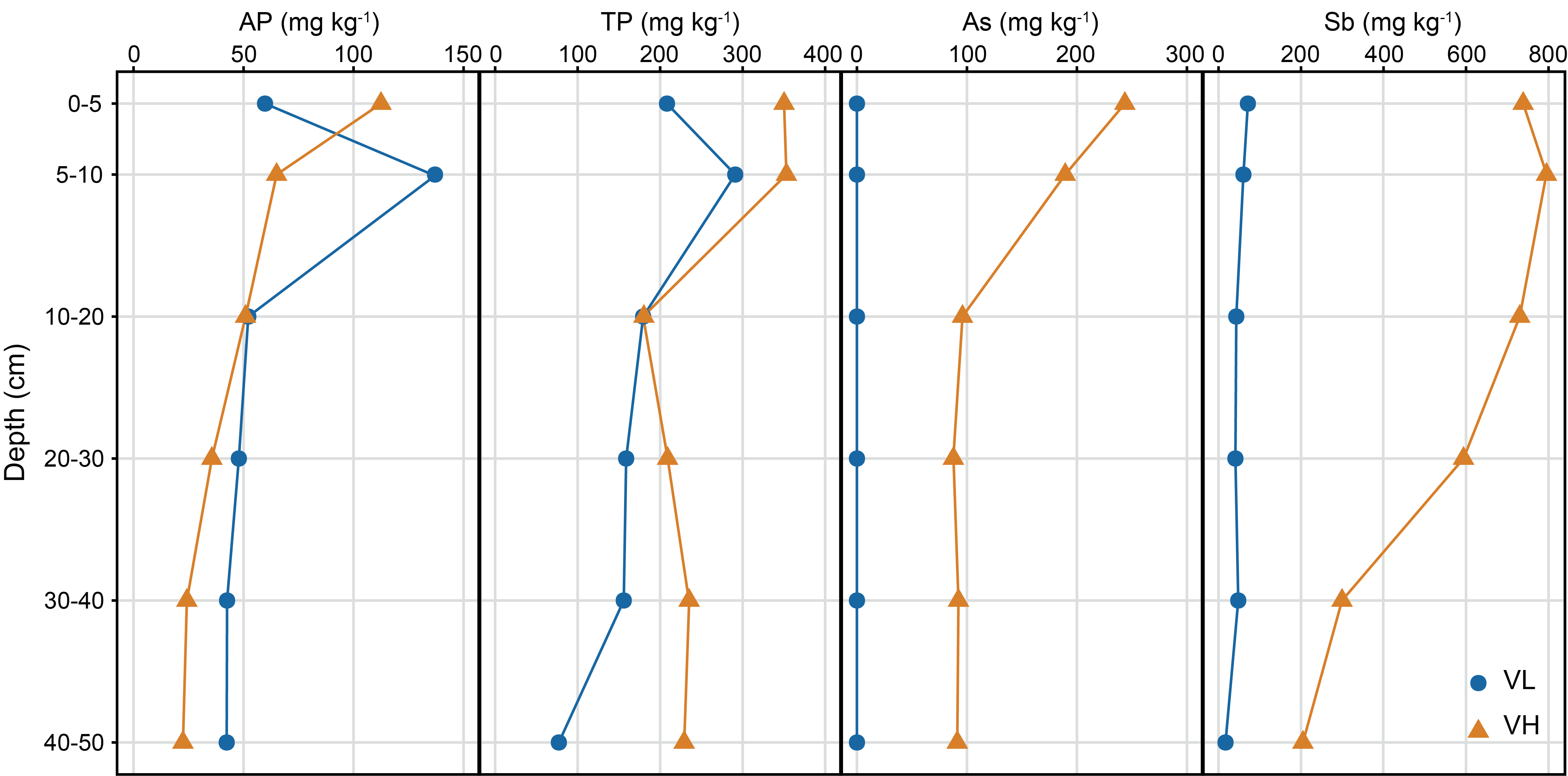


**Fig. S3 Comparison of the soil properties varied across the depth.** HL, horizontal low-contamination sites; HH, horizontal high-contamination sites; VL, vertical low-contamination sites; VH, vertical high-contamination sites; AP, available phosphorus; TP, total phosphorus; As, total arsenic; Sb, total antimony.


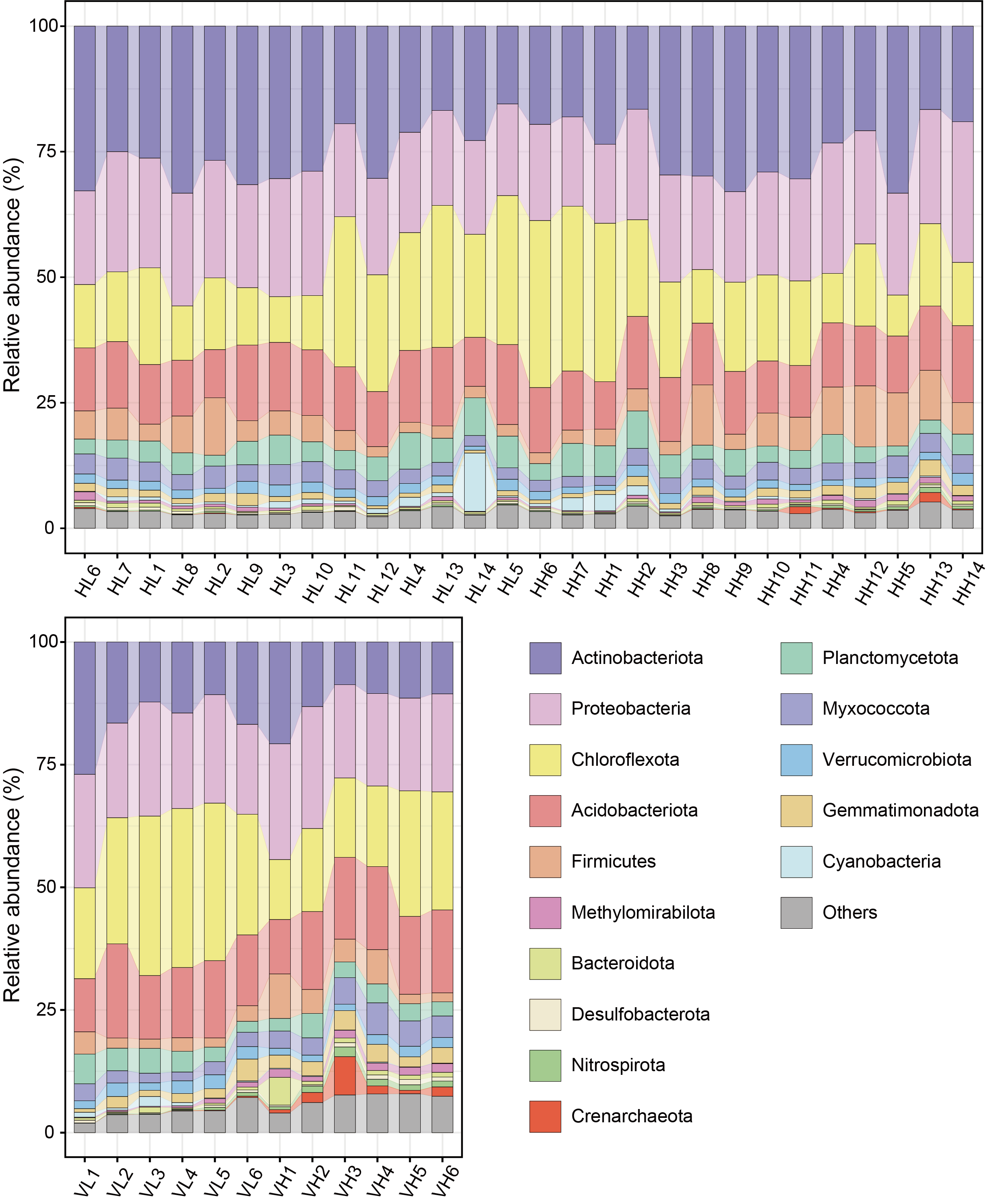


**Fig. S4 Relative abundance of the most abundant soil bacterial phyla in each sample.** HL, horizontal low-contamination sites; HH, horizontal high-contamination sites; VL, vertical low-contamination sites; VH, vertical high-contamination sites.

**
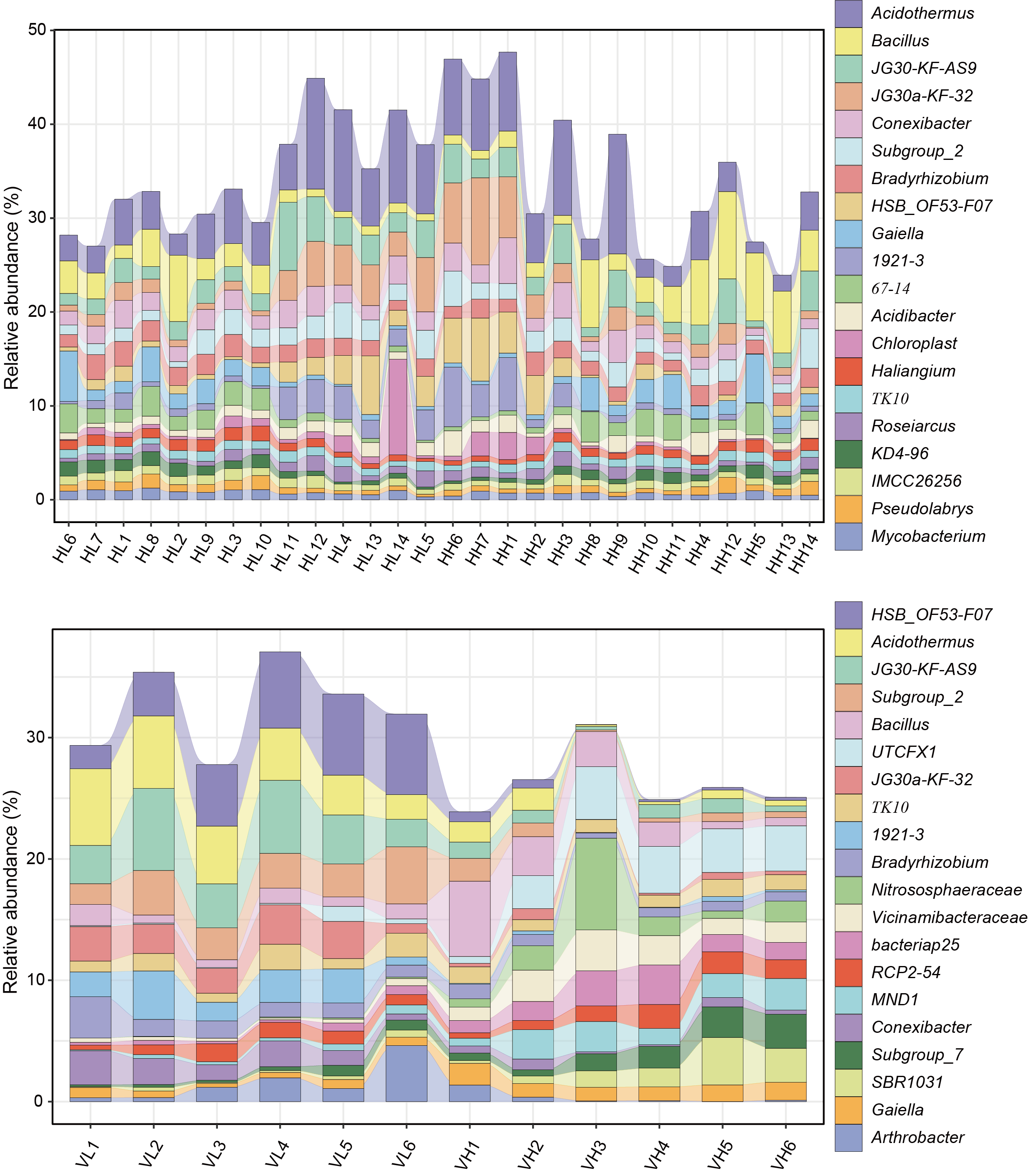
**

**Fig. S5 Relative abundance of the most abundant soil bacterial genera (top 20) in each sample.** HL, horizontal low-contamination sites; HH, horizontal high-contamination sites; VL, vertical low-contamination sites; VH, vertical high-contamination sites.


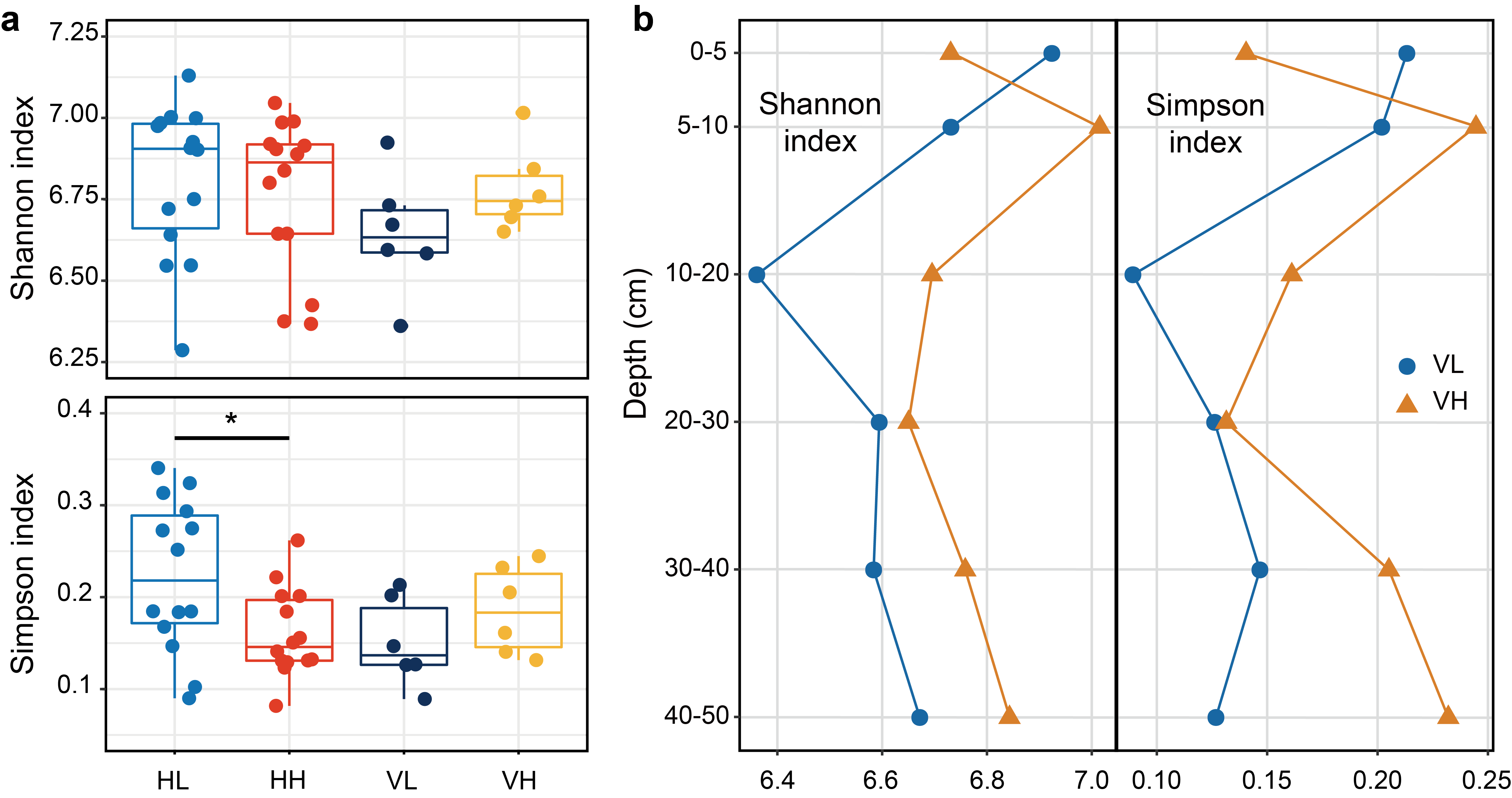


**Fig. S6 Comparison of the alpha diversity of bacterial communities (a) at four types of contaminated sites and (b) their vertical changes at two soil cores.** HL, horizontal low-contamination sites; HH, horizontal high-contamination sites; VL, vertical low-contamination sites; VH, vertical high-contamination sites. Significance levels were calculated by paired Student's *t*-test and denoted as **p* < 0.05.


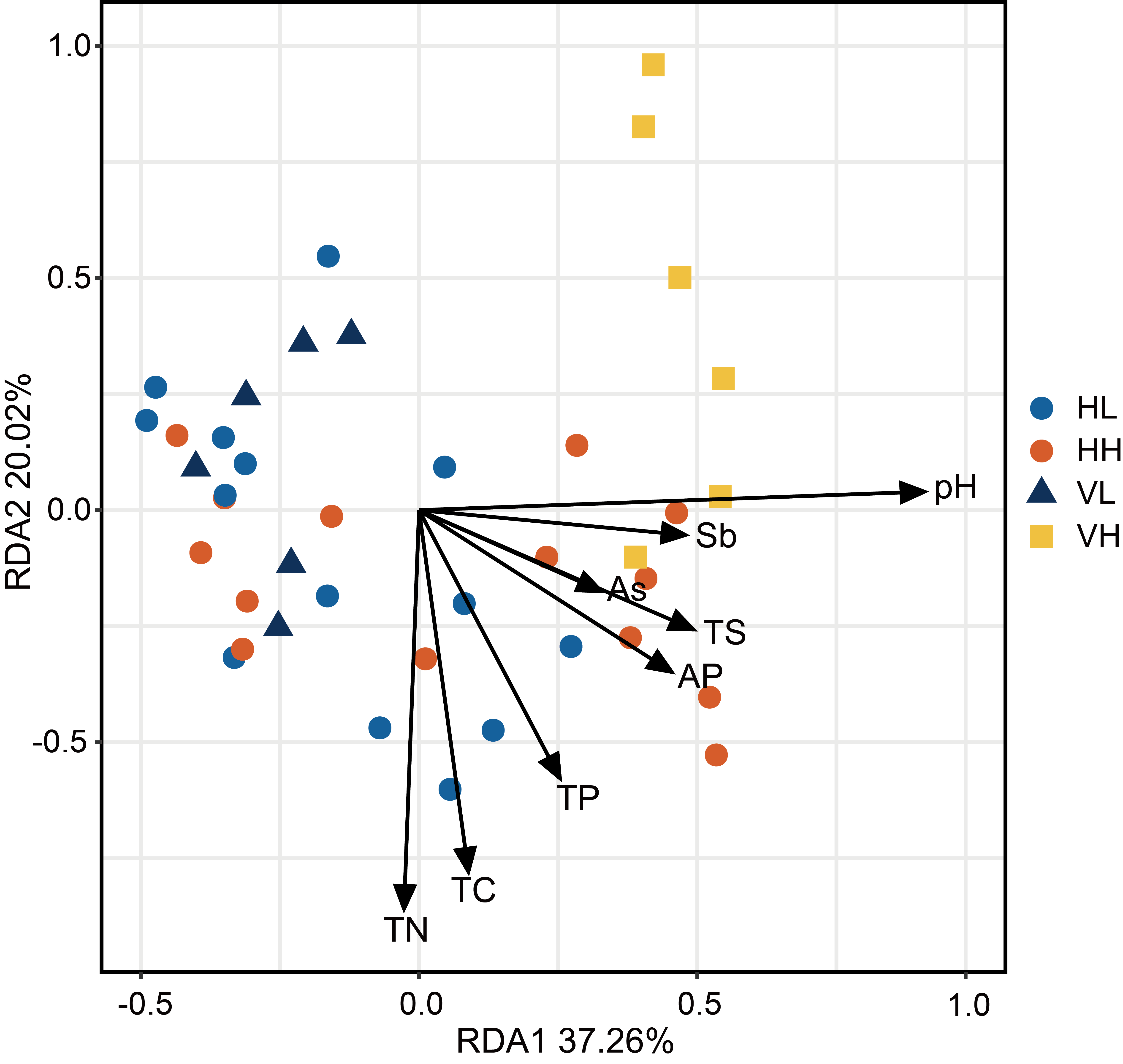


**Fig. S7 Redundancy analysis (RDA) of soil contaminants and nutrients for bacteria communities at four types of contaminated sites.** HL, horizontal low-contamination sites; HH, horizontal high-contamination sites; VL, vertical low-contamination sites; VH, vertical high-contamination sites; Sb, total antimony; As, total arsenic; TP, total phosphorus; AP, available phosphorus; TC, total carbon; TN, total nitrogen; TS, total sulfur.


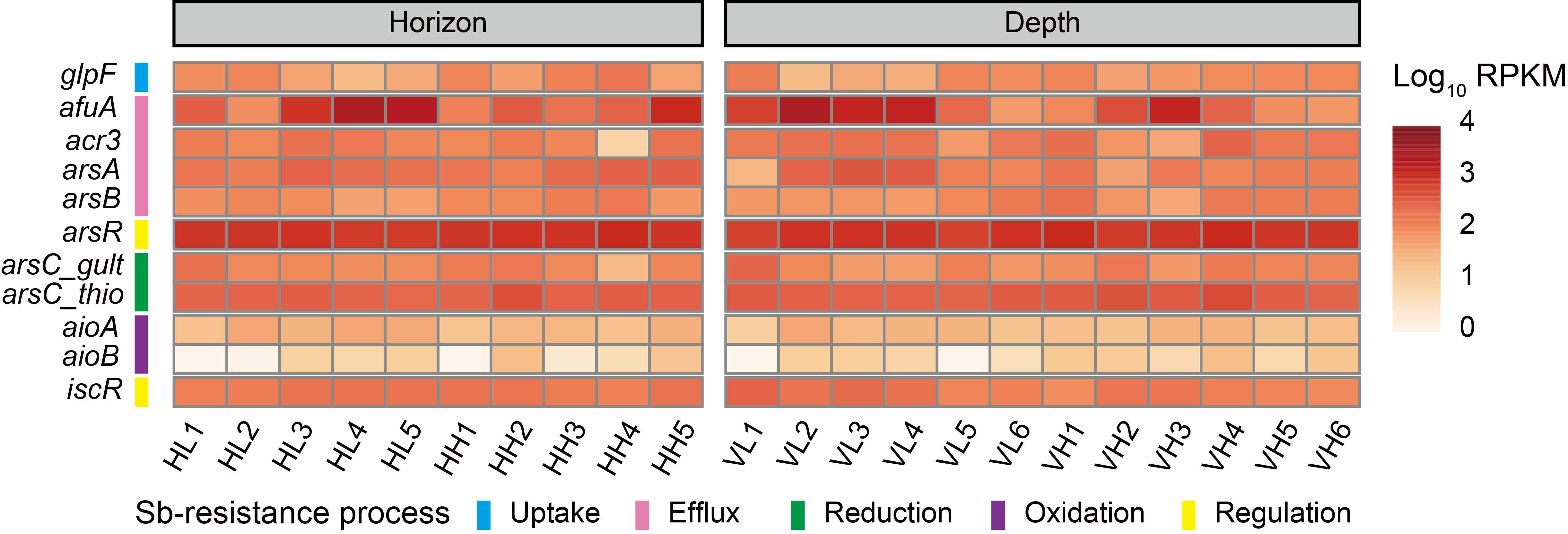


**Fig. S8 Relative abundance of soil microbial Sb-resistance genes at each sampling site.** HL, horizontal low-contamination sites; HH, horizontal high-contamination sites; VL, vertical low-contamination sites; VH, vertical high-contamination sites. The relative abundance of related genes was calculated as Log_10_ RPKM (reads per kilobase per million mapped reads).


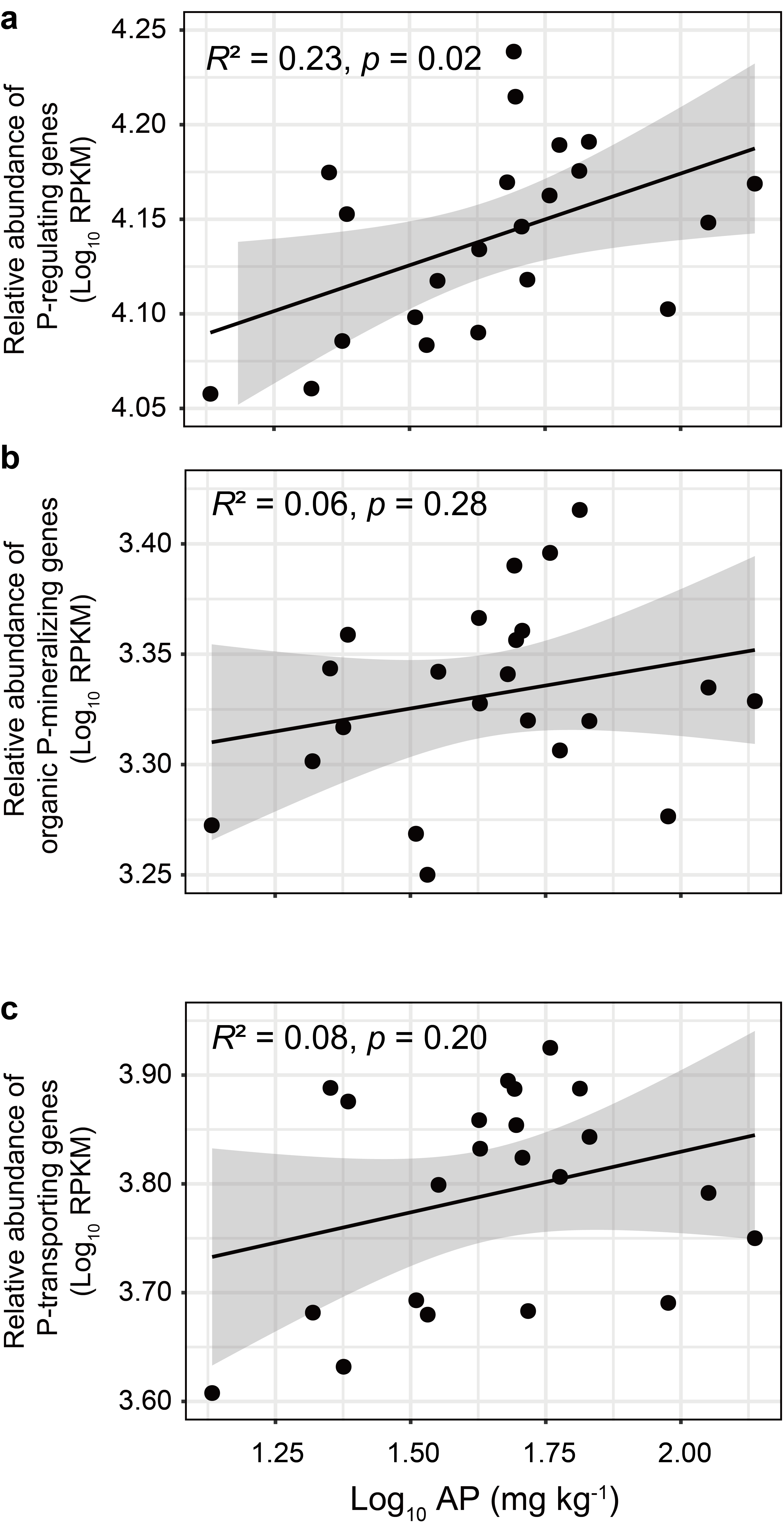


**Fig. S9 The linkages between P-regulating genes (a), P-mineralizing genes (b), P-transporting genes (c) and soil P availability, respectively.** Pearson’s correlation analysis was performed to assess the correlations between two parameters. The relative abundance of related genes was calculated as Log_10_ RPKM (reads per kilobase per million mapped reads).


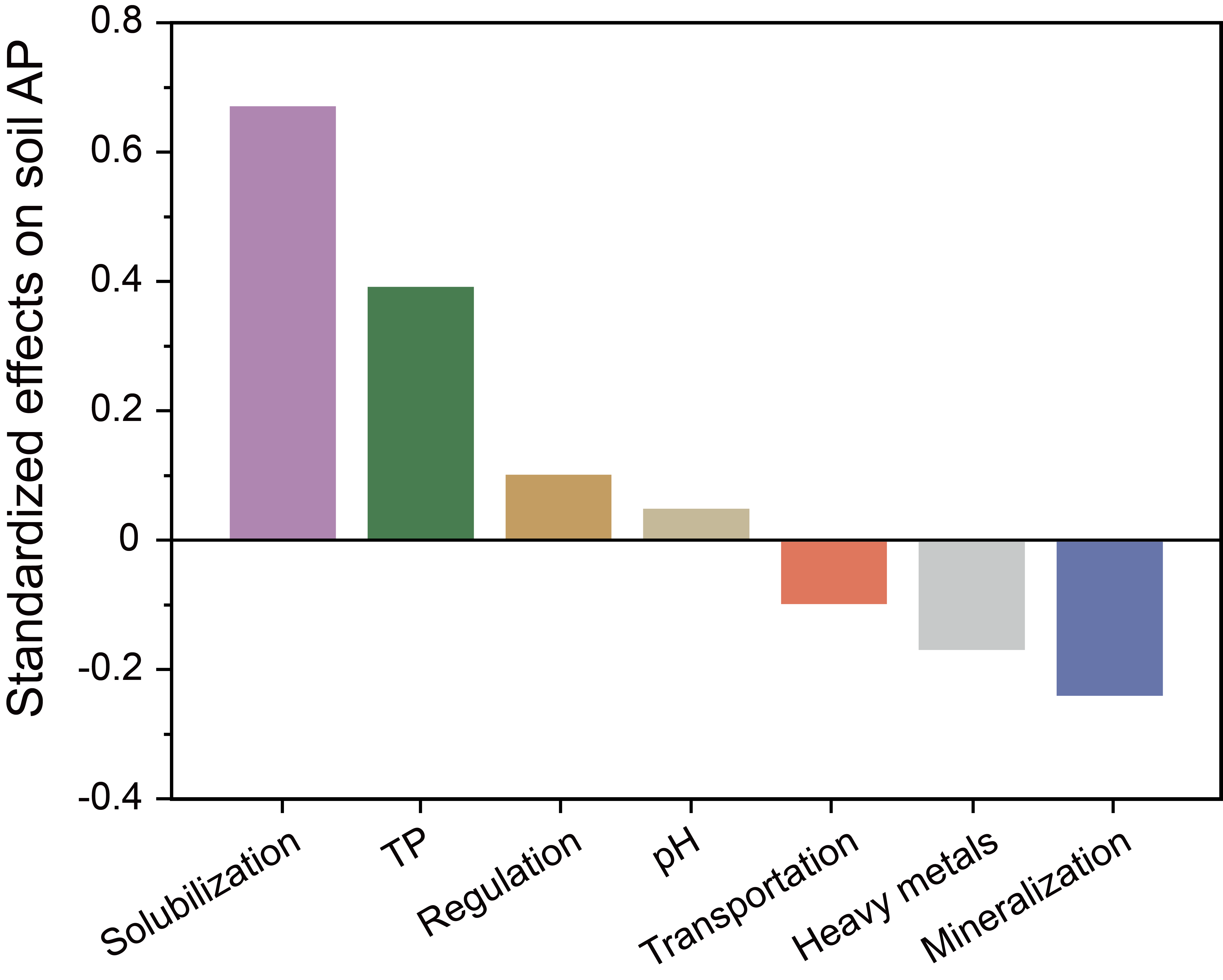


**Fig. S10 The standard effects of the environmental factors and microbial P-cycling potentials on soil P availability.** TP, total phosphorus; AP, available phosphorus.


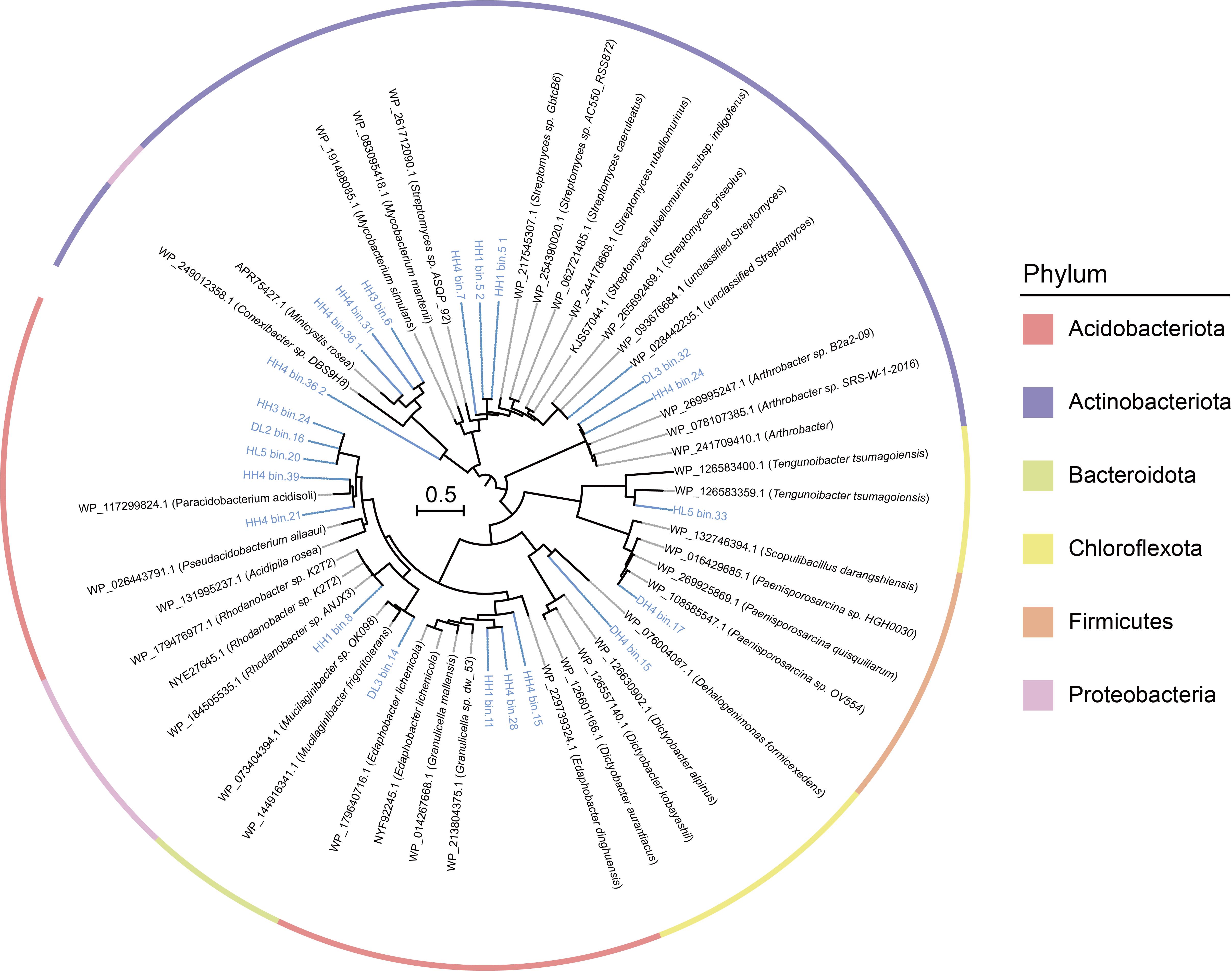


**Fig. 11** **Phylogenetic distribution of Sb efflux pump ArsB.** The maximum-likelihood phylogenetic tree after 1000 bootstraps showing ArsB sequences from the recovered MAGs (blue) alongside representative sequences from reference genomes (grey). The 0.5 scale bar indicates the tree scale.


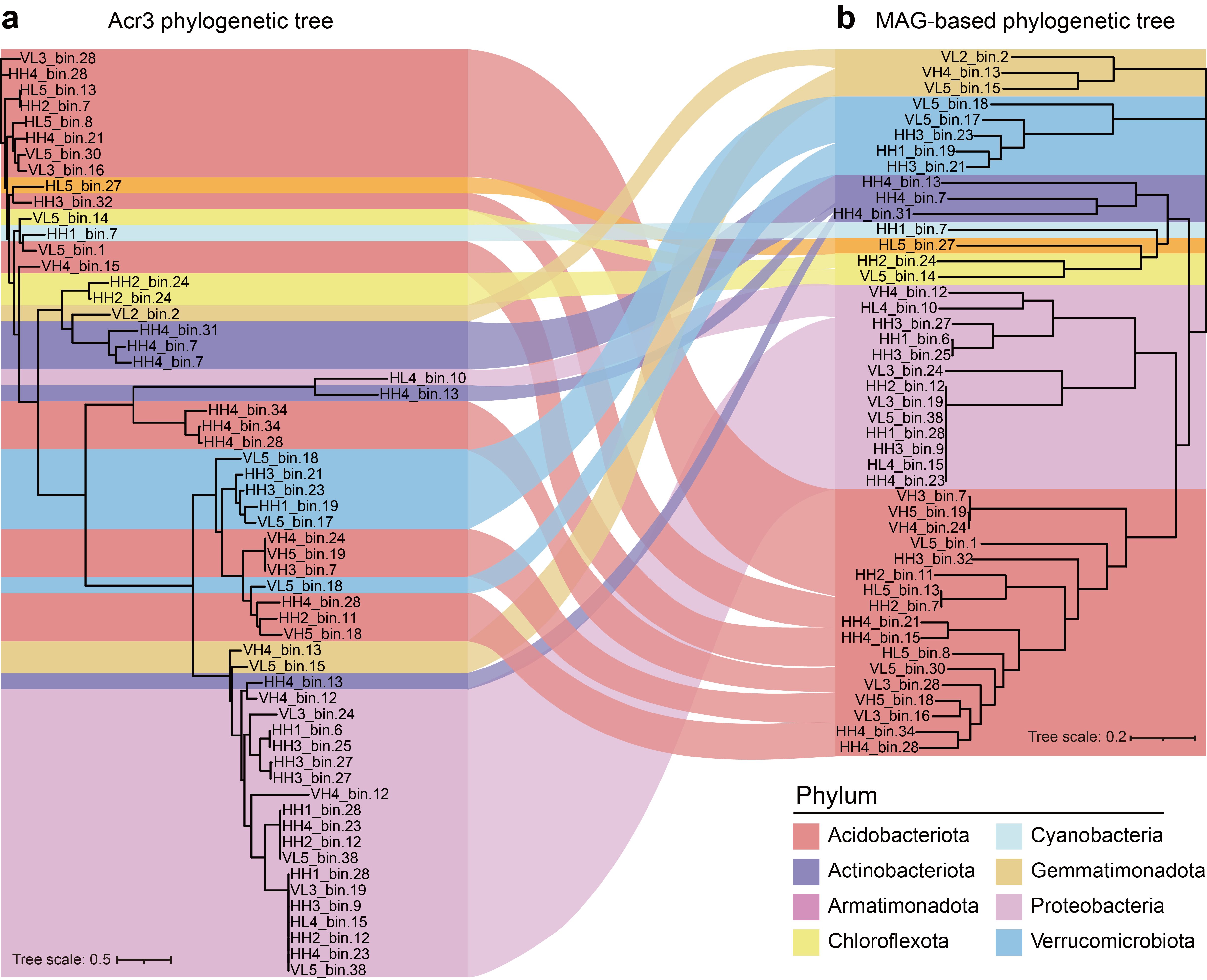


**Fig. S12 Comparison of MAG-based phylogenetic tree and Acr3 phylogenetic tree.** (a) Phylogenetic analysis of identified 58 Acr3 protein sequences. (b) Phylogenomic analysis of 45 MAGs containing the *acr3* gene. Each phylum is colored differently to identify horizontal gene transfer (HGT) based on inconsistent branching patterns.


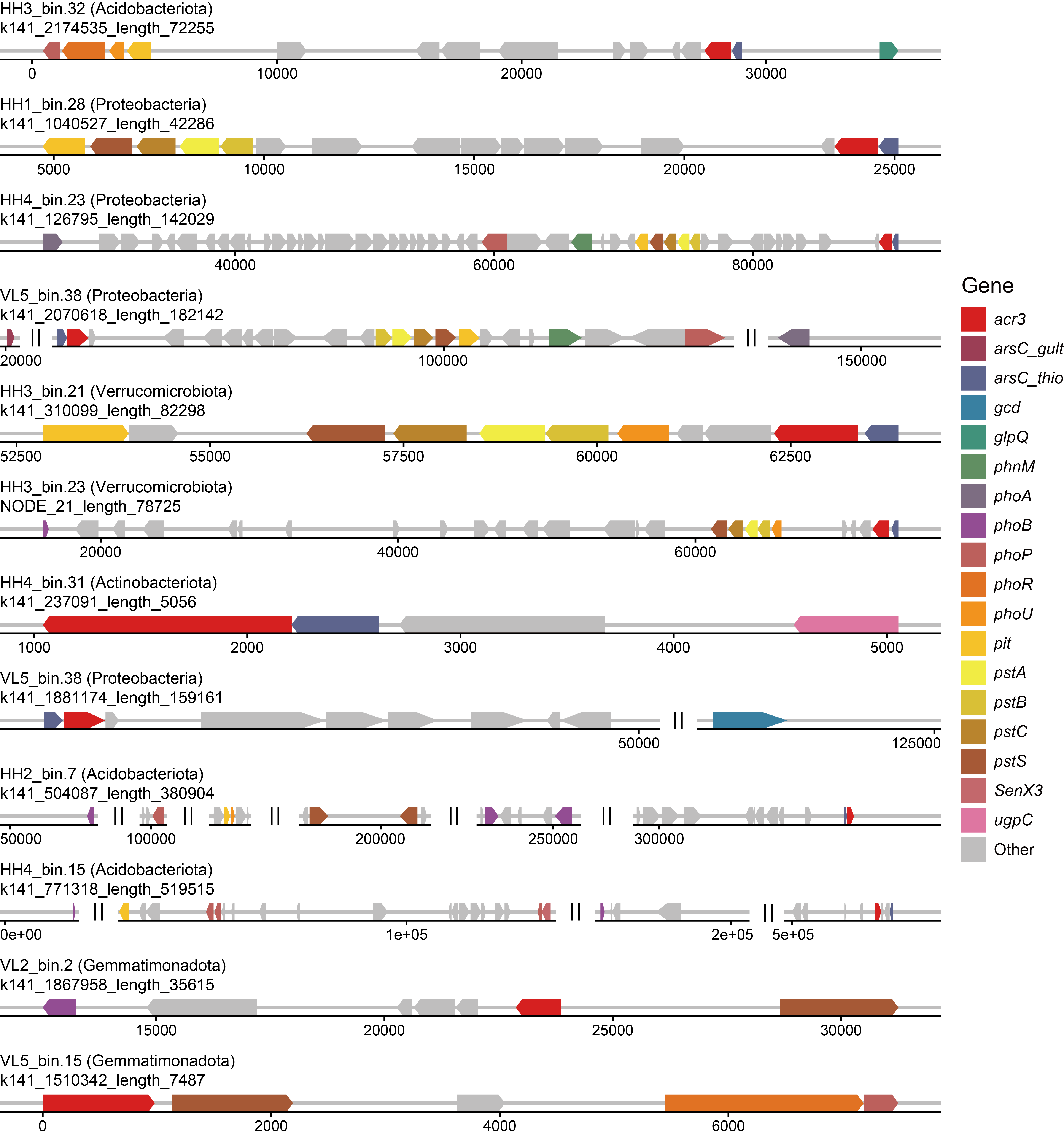


**Fig. S13 The genetic organization of the *acr3* gene, other Sb-resistance genes and P-cycling genes on the same contig within MAGs.**
