# Supplementary material for: Antimony efflux underpins phosphorus cycling and resistance of phosphate-solubilizing bacteria in mining soils
Source: ISME J. 2023 Jun 3;17(8):1278–89. doi: 10.1038/s41396-023-01445-6 (PMC10356851; doi:10.1038/s41396-023-01445-6)
Supplement: Supplementary file 2 — Supplementary table S1 [file 41396_2023_1445_MOESM2_ESM.docx]

**Table S1** The classification, gene name, function descriptions, KO number and relevant validation experiment strain referring to KEGG database and PCycDB.

| Classification | Gene | Details for gene function | KO number | Strain | Reference |
| --- | --- | --- | --- | --- | --- |
| Antimony resistance genes | | | | | |
| Antimony uptake | *glpF* | glycerol uptake facilitator protein | K02440 | *Escherichia coli* | Sanders *et al.*, 1997 |
|  |  |  |  | *Saccharomyces cerevisiae* | Wysocki *et al.*, 2001 |
|  |  |  |  | *Escherichia coli* | Meng *et al.*, 2004 |
| Antimony efflux | *afuA* | iron(III) transport system substrate-binding protein | K02012 | *Agrobacterium tumefaciens* GW4 | Li *et al.*, 2016 |
|  | *acr3* | arsenite transporter | K03325 | *Saccharomyces cerevisiae* | Wysocki *et al.*, 2001 |
|  |  |  |  | *Agrobacterium tumefaciens* 5A | Kang *et al.*, 2015 |
|  | *arsA* | arsenite/tail-anchored protein-transporting ATPase | K01551 | *Escherichia coli* | Tisa and Rosen, 1990 |
|  | *arsB* | arsenical pump membrane protein | K03893 | *Escherichia coli* | Tisa and Rosen, 1990 |
|  |  |  |  | *Synechocystis* sp. PCC 6803 | Lopez-Maury *et al.*, 2003 |
|  |  |  |  | *Escherichia coli* | Meng *et al.*, 2004 |
| Antimony reducction | *arsC_gult* | arsenate reductase (glutaredoxin) | K00537 | *Escherichia coli* | Tisa and Rosen, 1990 |
|  | *arsC_thio* | arsenate reductase (thioredoxin) | K03741 | *Escherichia coli* | Tisa and Rosen, 1990 |
| Antimony oxidation | *aioA* | arsenite oxidase large subunit (AioA) | K08356 | *Agrobacterium tumefaciens* 5A | Wang *et al.*, 2015 |
|  | *aioB* | arsenite oxidase small subunit (AioB) | K08355 | *Agrobacterium tumefaciens* 5A | Wang *et al.*, 2015 |
| Antimony regulation | *arsR* | ArsR family transcriptional regulator, arsenate/arsenite/antimonite-responsive transcriptional repressor | K03892 | *Escherichia coli* | Chen *et al.*, 1997 |
|  | *iscR* | Rrf2 family transcriptional regulator, iron-sulfur cluster assembly transcription factor | K13643 | *Comamonas testosteroni* S44 | Liu *et al.*, 2015 |
| Phosphorus cycling genes | | | | | |
| Inorganic phosphorus solubilization | *gcd* | quinoprotein glucose dehydrogenase | K00117 | PCycDB | Zeng *et al.*, 2022 |
|  | *ppa* | inorganic pyrophosphatase | K01507 |  |  |
|  | *ppk* | polyphosphate kinase | K00937 |  |  |
|  | *ppx* | exopolyphosphatase / guanosine-5'-triphosphate,3'-diphosphate pyrophosphatase | K01524 |  |  |
| Organic phosphorus mineralization | *appA* | 4-phytase / acid phosphatase | K01093 | PCycDB | Zeng *et al.*, 2022 |
|  | *glpQ* | glycerophosphoryl diester phosphodiesterase (periplasmic) about 333 aa | K01126 |  |  |
|  | *olpA* | acid phosphatase | K01078 |  |  |
|  | *opd* | phosphotriesterase (organophosphorus-degrading genes) | K07048 |  |  |
|  | *pafA* | phosphoate-insensitive phosphomonoesterase | / |  |  |
|  | *pbfA* | phosphonate breakdown factor A, catalyzes (R)-1-hydroxy-2-aminoethylphosphonate (R-HAEP) to generate phosphonoacetaldehyde | / |  |  |
|  | *phnA* | phosphonoacetate hydrolase | K19670 |  |  |
|  | *phnF* | GntR family transcriptional regulator, phosphonate transport system regulatory protein | K02043 |  |  |
|  | *phnG* | alpha-D-ribose 1-methylphosphonate 5-triphosphate synthase subunit PhnG | K06166 |  |  |
|  | *phnH* | alpha-D-ribose 1-methylphosphonate 5-triphosphate synthase subunit PhnH | K06165 |  |  |
|  | *phnI* | alpha-D-ribose 1-methylphosphonate 5-triphosphate synthase subunit PhnI | K06164 |  |  |
|  | *phnJ* | alpha-D-ribose 1-methylphosphonate 5-phosphate C-P lyase | K06163 |  |  |
|  | *phnK* | alpha-D-ribose 1-methylphosphonate 5-triphosphate synthase C-P lyase | K05781 |  |  |
|  | *phnL* | alpha-D-ribose 1-methylphosphonate 5-triphosphate synthase subunit PhnL | K05780 |  |  |
|  | *phnM* | alpha-D-ribose 1-methylphosphonate 5-triphosphate diphosphatase | K06162 |  |  |
|  | *phnN* | ribose 1,5-bisphosphokinase | K05774 |  |  |
|  | *phnO* | (aminoalkyl) phosphonate N-acetyltransferase | K09994 | PCycDB | Zeng *et al.*, 2022 |
|  | *phnP* | phosphoribosyl 1,2-cyclic phosphate phosphodiesterase | K06167 |  |  |
|  | *phnW* | 2-aminoethylphosphonate-pyruvate transaminase | K03430 |  |  |
|  | *phnX* | phosphonoacetaldehyde hydrolase | K05306 |  |  |
|  | *phnY* | 2-aminoethylphosphonate-pyruvate transaminase | K21195 |  |  |
|  | *phoA* | alkaline phosphatase | K01077 |  |  |
|  | *phoC* | acid phosphatase | / |  |  |
|  | *phoD* | alkaline phosphatase D | K01113 |  |  |
|  | *phoN* | acid phosphatase (class A) | K09474 |  |  |
|  | *phoX* | alkaline phosphatase | K07093 |  |  |
|  | *phy* | 3-phytase | K01083 |  |  |
|  | *ugpQ* | glycerophosphoryl diester phosphodiesterase (cytoplasmic) about 247 aa | K01126 |  |  |
| Phosphorus transportation | *phnC* | phosphonate transport system ATP-binding protein | K02041 |  |  |
|  | *phnD* | phosphonate transport system substrate-binding protein | K02044 |  |  |
|  | *phnE* | phosphonate transport system permease protein | K02042 |  |  |
|  | *phnS* | 2-aminoethylphosphonate transport system substrate-binding protein | K11081 |  |  |
|  | *phnT* | 2-aminoethylphosphonate transport system ATP-binding protein | K11084 |  |  |
|  | *phnU* | 2-aminoethylphosphonate transport system permease protein | K11083 |  |  |
|  | *phnV* | 2-aminoethylphosphonate transport system permease protein | K11082 |  |  |
|  | *pit* | inorganic phosphate transporter | K03306 | PCycDB | Zeng *et al.*, 2022 |
|  | *pstA* | phosphate transport system permease protein | K02038 |  |  |
|  | *pstB* | phosphate transport system ATP-binding protein | K02036 |  |  |
|  | *pstC* | phosphate transport system permease protein | K02037 |  |  |
|  | *pstS* | phosphate transport system substrate-binding protein | K02040 |  |  |
|  | *ugpA* | sn-glycerol 3-phosphate transport system permease protein | K05814 |  |  |
|  | *ugpB* | sn-glycerol 3-phosphate transport system substrate-binding protein | K05813 |  |  |
|  | *ugpC* | sn-glycerol 3-phosphate transport system ATP-binding protein | K05816 |  |  |
|  | *ugpE* | sn-glycerol 3-phosphate transport system permease protein | K05815 |  |  |
| Phosphorus regulation | *phoB* | two-component system, OmpR family, phosphate regulon response regulator PhoB | K07657 |  |  |
|  | *phoP* | PhoP; two-component system, OmpR family, alkaline phosphatase synthesis response regulator PhoP | K07658, K07660 |  |  |
|  | *phoR* | two-component system, OmpR family, phosphate regulon sensor histidine kinase PhoR | K07636 |  |  |
|  | *phoU* | PhoR/PhoB inhibitor protein PhoU | K02039 |  |  |
|  | *RegX3* | two-component system, OmpR family, response regulator RegX3 | K07776 |  |  |
|  | *SenX3* | two-component system, OmpR family, sensor histidine kinase SenX3 | K07768 |  |  |

**Reference**

Chen Y, Rosen BP. Metalloregulatory properties of the ArsD repressor. *J Biol Chem* 1997; **272**: 14257–14262.

Kang Y, Shi Z, Bothner B, Wang G, McDermott TR. Involvement of the Acr3 and DctA anti-porters in arsenite oxidation in *A* *grobacterium tumefaciens* 5A: antiporter involvement in arsenite oxidation. *Environ Microbiol* 2015; **17**: 1950–1962.

Li J, Wang Q, Oremland RS, Kulp TR, Rensing C, Wang G. Microbial antimony biogeochemistry: enzymes, regulation, and related metabolic pathways. *Appl Environ Microbiol* 2016; **82**: 5482–5495.

Liu H, Zhuang W, Zhang S, Rensing C, Huang J, Li J, et al. Global regulator IscR positively contributes to antimonite resistance and oxidation in *Comamonas testosteroni* S44. *Front Mol Biosci* 2015; **2**: 70.

López-Maury L, Florencio FJ, Reyes JC. Arsenic sensing and resistance system in the cyanobacterium *Synechocystis* sp. strain PCC 6803. *J Bacteriol* 2003; **185**: 5363–5371.

Meng Y, Liu Z, Rosen BP. As(III) and Sb(III) uptake by GlpF and efflux by ArsB in *Escherichia coli*. *J Biol Chem* 2004; **279**: 18334–18341.

Sanders OI, Rensing C, Kuroda M, Mitra B, Rosen BP. Antimonite is accumulated by the glycerol facilitator GlpF in *Escherichia coli*. *J Bacteriol* 1997; **179**: 3365–3367.

Tisa LS, Rosen BP. Molecular characterization of an anion pump. The ArsB protein is the membrane anchor for the ArsA protein. *J Biol Chem* 1990; **265**: 190–194.

Wang Q, Warelow TP, Kang Y-S, Romano C, Osborne TH, Lehr CR, et al. Arsenite oxidase also functions as an antimonite oxidase. *Appl Environ Microbiol* 2015; **81**: 1959–1965.

Wysocki R, Chéry CC, Wawrzycka D, Van Hulle M, Cornelis R, Thevelein JM, et al. The glycerol channel Fps1p mediates the uptake of arsenite and antimonite in *Saccharomyces cerevisiae*. *Mol Microbiol* 2001; **40**: 1391–1401.

Zeng J, Tu Q, Yu X, Qian L, Wang C, Shu L, et al. PCycDB: a comprehensive and accurate database for fast analysis of phosphorus cycling genes. *Microbiome* 2022; **10**: 101.
