# Supplementary material for: Antimony efflux underpins phosphorus cycling and resistance of phosphate-solubilizing bacteria in mining soils
Source: ISME J. 2023 Jun 3;17(8):1278–89. doi: 10.1038/s41396-023-01445-6 (PMC10356851; doi:10.1038/s41396-023-01445-6)
Supplement: Supplementary file 3 — Supplementary table S2 [file 41396_2023_1445_MOESM3_ESM.docx]

**Table S2** The qualities and taxonomic classification of 106 high-quality recovered MAGs in this study.

| Genome | Completeness (%) | Contamination (%) | GC (%) | Size (bp) | N50 | Classification |
| --- | --- | --- | --- | --- | --- | --- |
| HL4_bin.10 | 96.15 | 1.931 | 62.0 | 5982882 | 25281 | d__Bacteria;p__Proteobacteria;c__Alphaproteobacteria;o__Rhizobiales;f__Xanthobacteraceae;g__Bradyrhizobium |
| HL4_bin.15 | 95.45 | 1.388 | 50.5 | 2730736 | 15028 | d__Bacteria;p__Proteobacteria;c__Gammaproteobacteria;o__Burkholderiales;f__Methylophilaceae;g__Methylophilus |
| HL4_bin.16 | 93.16 | 1.709 | 57.2 | 5443726 | 207783 | d__Bacteria;p__Acidobacteriota;c__Acidobacteriae;o__Acidoferrales;f__UBA7541;g__Acidoferrum |
| HL5_bin.8 | 97.38 | 1.329 | 57.4 | 5765476 | 42765 | d__Bacteria;p__Acidobacteriota;c__Acidobacteriae;o__Acidobacteriales;f__CAINCZ01 |
| HL5_bin.13 | 95.51 | 1.709 | 57.8 | 4201396 | 188757 | d__Bacteria;p__Acidobacteriota;c__Acidobacteriae;o__Acidoferrales;f__UBA7541 |
| HL5_bin.18 | 99.32 | 2.162 | 54.7 | 4475044 | 266730 | d__Bacteria;p__Verrucomicrobiota;c__Verrucomicrobiae;o__Pedosphaerales;f__UBA11358 |
| HL5_bin.20 | 91.04 | 4.273 | 62.6 | 4636333 | 18976 | d__Bacteria;p__Acidobacteriota;c__Acidobacteriae;o__Acidobacteriales;f__Acidobacteriaceae;g__Palsa-343 |
| HL5_bin.23 | 93.61 | 1.574 | 67.1 | 4089154 | 9872 | d__Bacteria;p__Dormibacterota;c__Dormibacteria;o__Dormibacterales |
| HL5_bin.27 | 95.37 | 2.006 | 65.0 | 5314505 | 27274 | d__Bacteria;p__Armatimonadota;c__Chthonomonadetes;o__Chthonomonadales;f__Chthonomonadaceae |
| HL5_bin.30 | 98.64 | 4.093 | 51.4 | 3216962 | 39095 | d__Bacteria;p__Verrucomicrobiota;c__Verrucomicrobiae;o__Pedosphaerales;f__UBA11358 |
| HL5_bin.33 | 95.21 | 3.96 | 65.8 | 4334621 | 13279 | d__Bacteria;p__Chloroflexota;c__Ktedonobacteria;o__Ktedonobacterales |
| HH1_bin.5 | 90.74 | 4.965 | 69.7 | 7318554 | 17788 | d__Bacteria;p__Actinobacteriota;c__Actinomycetia;o__Streptosporangiales;f__Streptosporangiaceae;g__JAFARZ01 |
| HH1_bin.6 | 90.38 | 2.608 | 67.0 | 3727488 | 8742 | d__Bacteria;p__Proteobacteria;c__Alphaproteobacteria;o__ATCC43930;f__Stellaceae;g__AP-15 |
| HH1_bin.7 | 94.51 | 0.489 | 37.6 | 5368471 | 11936 | d__Bacteria;p__Cyanobacteria;c__Cyanobacteriia;o__Cyanobacteriales;f__Nostocaceae |
| HH1_bin.8 | 90.97 | 1.995 | 63.0 | 3870553 | 14878 | d__Bacteria;p__Proteobacteria;c__Gammaproteobacteria;o__Xanthomonadales;f__Rhodanobacteraceae;g__Rhodanobacter;s__Rhodanobacter sp014200755 |
| HH1_bin.11 | 91.31 | 3.276 | 56.7 | 4526946 | 60133 | d__Bacteria;p__Acidobacteriota;c__Acidobacteriae;o__Acidobacteriales;f__SbA1;g__Sulfotelmatobacter |
| HH1_bin.19 | 92.17 | 2.027 | 55.9 | 2869888 | 8531 | d__Bacteria;p__Verrucomicrobiota;c__Verrucomicrobiae;o__Pedosphaerales;f__UBA11358;g__UBA11358 |
| HH1_bin.21 | 90.22 | 4.594 | 60.7 | 4491549 | 23175 | d__Bacteria;p__Acidobacteriota;c__Acidobacteriae;o__Acidobacteriales;f__Acidobacteriaceae;g__Terracidiphilus |
| HH1_bin.25 | 90.81 | 4.914 | 57.7 | 3827814 | 30912 | d__Bacteria;p__Acidobacteriota;c__Acidobacteriae;o__Acidoferrales;f__UBA7541 |
| HH1_bin.28 | 99.85 | 0.596 | 50.0 | 3204878 | 51945 | d__Bacteria;p__Proteobacteria;c__Gammaproteobacteria;o__Burkholderiales;f__Methylophilaceae;g__Methylophilus |
| HH2_bin.7 | 95.51 | 1.709 | 58.1 | 3821334 | 257519 | d__Bacteria;p__Acidobacteriota;c__Acidobacteriae;o__Acidoferrales;f__UBA7541 |
| HH2_bin.8 | 92.42 | 2.517 | 68.3 | 3839702 | 10835 | d__Bacteria;p__Proteobacteria;c__Alphaproteobacteria;o__ATCC43930;f__Stellaceae;g__AP-15 |
| HH2_bin.9 | 95.29 | 2.564 | 58.9 | 4704081 | 177801 | d__Bacteria;p__Acidobacteriota;c__Acidobacteriae;o__Acidoferrales;f__UBA7541 |
| HH2_bin.11 | 94.65 | 1.709 | 58.1 | 4571963 | 91448 | d__Bacteria;p__Acidobacteriota;c__Acidobacteriae;o__Acidoferrales;f__UBA7541;g__Acidoferrum |
| HH2_bin.12 | 93.68 | 1.093 | 50.1 | 2900196 | 19422 | d__Bacteria;p__Proteobacteria;c__Gammaproteobacteria;o__Burkholderiales;f__Methylophilaceae;g__Methylophilus |
| HH2_bin.18 | 97.28 | 0 | 63.5 | 6873976 | 40169 | d__Bacteria;p__Planctomycetota;c__Planctomycetia;o__Isosphaerales;f__Isosphaeraceae |
| HH2_bin.22 | 99.56 | 1.739 | 61.6 | 4758574 | 111129 | d__Bacteria;p__Proteobacteria;c__Alphaproteobacteria;o__Micropepsales;f__Micropepsaceae;g__Rhizomicrobium |
| HH2_bin.23 | 97.97 | 4.589 | 56.6 | 5134355 | 119541 | d__Bacteria;p__Verrucomicrobiota;c__Verrucomicrobiae;o__Pedosphaerales;f__UBA3939;g__UBA3939 |
| HH2_bin.24 | 98.18 | 3.181 | 55.2 | 6929065 | 31053 | d__Bacteria;p__Chloroflexota;c__Anaerolineae;o__Aggregatilineales;f__UBA2796 |
| HH3_bin.1 | 98.06 | 2.291 | 40.5 | 4062658 | 75745 | d__Bacteria;p__Bacteroidota;c__Bacteroidia;o__Cytophagales;f__Cyclobacteriaceae;g__ELB16-189 |
| HH3_bin.6 | 91.14 | 2.729 | 69.0 | 2875107 | 30112 | d__Bacteria;p__Actinobacteriota;c__Thermoleophilia;o__Gaiellales;f__Gaiellaceae;g__Palsa-739 |
| HH3_bin.7 | 95.94 | 4.431 | 51.4 | 3230847 | 27494 | d__Bacteria;p__Verrucomicrobiota;c__Verrucomicrobiae;o__Pedosphaerales;f__UBA11358 |
| HH3_bin.9 | 90.04 | 0.427 | 50.8 | 2471306 | 7963 | d__Bacteria;p__Proteobacteria;c__Gammaproteobacteria;o__Burkholderiales;f__Methylophilaceae;g__Methylophilus |
| HH3_bin.11 | 98.27 | 0.862 | 59.2 | 7177081 | 157948 | d__Bacteria;p__Acidobacteriota;c__Acidobacteriae;o__Acidobacteriales;f__Acidobacteriaceae;g__Terracidiphilus |
| HH3_bin.15 | 92.31 | 1.948 | 44.4 | 4376237 | 21624 | d__Bacteria;p__Bacteroidota;c__Bacteroidia;o__Cytophagales;f__Cyclobacteriaceae;g__ELB16-189 |
| HH3_bin.17 | 90.28 | 4.287 | 65.5 | 2649197 | 36455 | d__Bacteria;p__Proteobacteria;c__Gammaproteobacteria;o__REEB76;f__REEB76 |
| HH3_bin.21 | 97.63 | 4.054 | 59.5 | 3971510 | 98905 | d__Bacteria;p__Verrucomicrobiota;c__Verrucomicrobiae;o__Pedosphaerales;f__UBA11358 |
| HH3_bin.23 | 93.58 | 4.759 | 58.2 | 4792950 | 69532 | d__Bacteria;p__Verrucomicrobiota;c__Verrucomicrobiae;o__Pedosphaerales;f__UBA11358;g__UBA7542 |
| HH3_bin.24 | 95.25 | 3.076 | 62.5 | 4858542 | 16060 | d__Bacteria;p__Acidobacteriota;c__Acidobacteriae;o__Acidobacteriales;f__Acidobacteriaceae;g__Palsa-343 |
| HH3_bin.25 | 96.73 | 2.144 | 66.8 | 4053788 | 13907 | d__Bacteria;p__Proteobacteria;c__Alphaproteobacteria;o__ATCC43930;f__Stellaceae;g__AP-15 |
| HH3_bin.27 | 90.95 | 3.405 | 69.5 | 4105594 | 8588 | d__Bacteria;p__Proteobacteria;c__Alphaproteobacteria;o__ATCC43930;f__Stellaceae;g__AP-15 |
| HH3_bin.29 | 94.9 | 2.551 | 67.4 | 3971087 | 12494 | d__Bacteria;p__Dormibacterota;c__Dormibacteria;o__Dormibacterales |
| HH3_bin.32 | 96.31 | 0.854 | 57.9 | 4662289 | 98648 | d__Bacteria;p__Acidobacteriota;c__Acidobacteriae;o__UBA7540;f__UBA7540 |
| HH4_bin.4 | 98.31 | 1.123 | 55.9 | 3852853 | 145940 | d__Bacteria;p__FCPU426;c__Palsa-1180;o__Palsa-1180;f__Palsa-1180;g__PALSA-1180 |
| HH4_bin.7 | 95.36 | 2.6 | 67.1 | 4477544 | 29425 | d__Bacteria;p__Actinobacteriota;c__Actinomycetia;o__Mycobacteriales;f__Pseudonocardiaceae;g__GCA-003244245 |
| HH4_bin.13 | 93.63 | 1.566 | 68.2 | 3850338 | 50648 | d__Bacteria;p__Actinobacteriota;c__Thermoleophilia;o__Solirubrobacterales;f__Solirubrobacteraceae;g__Palsa-744 |
| HH4_bin.15 | 94.77 | 1.724 | 59.8 | 5388741 | 364582 | d__Bacteria;p__Acidobacteriota;c__Acidobacteriae;o__Acidobacteriales;f__Acidobacteriaceae;g__Terracidiphilus |
| HH4_bin.19 | 96.33 | 0.095 | 62.2 | 5289716 | 67517 | d__Bacteria;p__Acidobacteriota;c__Acidobacteriae;o__Acidobacteriales;f__Acidobacteriaceae;g__Terracidiphilus |
| HH4_bin.21 | 97.19 | 1.724 | 59.6 | 5357563 | 257049 | d__Bacteria;p__Acidobacteriota;c__Acidobacteriae;o__Acidobacteriales;f__Acidobacteriaceae;g__Terracidiphilus |
| HH4_bin.23 | 95.72 | 0.427 | 50.2 | 2968113 | 57740 | d__Bacteria;p__Proteobacteria;c__Gammaproteobacteria;o__Burkholderiales;f__Methylophilaceae;g__Methylophilus |
| HH4_bin.24 | 93.67 | 4.005 | 64.1 | 4173328 | 10401 | d__Bacteria;p__Actinobacteriota;c__Actinomycetia;o__Actinomycetales;f__Micrococcaceae;g__Arthrobacter_K;s__Arthrobacter_K sp001549895 |
| HH4_bin.28 | 92.04 | 3.656 | 57.9 | 5566770 | 10975 | d__Bacteria;p__Acidobacteriota;c__Acidobacteriae;o__Acidobacteriales;f__SbA1;g__Sulfotelmatobacter |
| HH4_bin.30 | 92.57 | 1.923 | 62.2 | 3670610 | 30247 | d__Bacteria;p__Proteobacteria;c__Alphaproteobacteria;o__Rhizobiales;f__Xanthobacteraceae;g__BOG-931 |
| HH4_bin.31 | 90.59 | 2.136 | 72.2 | 1948255 | 16418 | d__Bacteria;p__Actinobacteriota;c__Acidimicrobiia;o__Acidimicrobiales;f__RAAP-2;g__RAAP-2 |
| HH4_bin.34 | 99.66 | 0.854 | 55.3 | 4174240 | 294169 | d__Bacteria;p__Acidobacteriota;c__Acidobacteriae;o__Acidobacteriales;f__SbA1;g__PALSA-188 |
| HH4_bin.36 | 95.44 | 2.594 | 62.5 | 1988795 | 14614 | d__Bacteria;p__Actinobacteriota;c__Acidimicrobiia;o__Acidimicrobiales;f__RAAP-2;g__RAAP-2 |
| HH4_bin.39 | 99.14 | 2.564 | 62.9 | 5558141 | 274505 | d__Bacteria;p__Acidobacteriota;c__Acidobacteriae;o__Acidobacteriales;f__Acidobacteriaceae;g__Palsa-343 |
| VL1_bin.3 | 93.88 | 2.806 | 64.7 | 4744455 | 10692 | d__Bacteria;p__Acidobacteriota;c__Acidobacteriae;o__Acidobacteriales;f__CAINCZ01 |
| VL2_bin.2 | 93.21 | 3.862 | 64.7 | 5433558 | 10871 | d__Bacteria;p__Gemmatimonadota;c__Gemmatimonadetes;o__Gemmatimonadales;f__Gemmatimonadaceae;g__AG2 |
| VL2_bin.16 | 92.57 | 4.914 | 62.4 | 5088594 | 18949 | d__Bacteria;p__Acidobacteriota;c__Acidobacteriae;o__Acidobacteriales;f__Acidobacteriaceae;g__Palsa-343 |
| VL2_bin.20 | 94.38 | 2.247 | 54.2 | 3238833 | 7038 | d__Bacteria;p__FCPU426;c__Palsa-1180;o__Palsa-1180;f__Palsa-1180;g__PALSA-1180 |
| VL2_bin.21 | 95.83 | 2.314 | 64.9 | 3107047 | 15399 | d__Bacteria;p__Dormibacterota;c__Dormibacteria;o__CF-121;f__CF-121;g__CF-13 |
| VL3_bin.3 | 94.86 | 3.149 | 70.2 | 4338196 | 20396 | d__Bacteria;p__Proteobacteria;c__Alphaproteobacteria;o__Caulobacterales;f__Caulobacteraceae;g__Phenylobacterium |
| VL3_bin.10 | 90.86 | 3.418 | 54.8 | 6222605 | 10977 | d__Bacteria;p__Acidobacteriota;c__Acidobacteriae;o__Acidobacteriales;f__Gp1-AA117;g__Gp1-AA17 |
| VL3_bin.14 | 97.46 | 1.428 | 41.5 | 6839676 | 172368 | d__Bacteria;p__Bacteroidota;c__Bacteroidia;o__Sphingobacteriales;f__Sphingobacteriaceae;g__Mucilaginibacter |
| VL3_bin.16 | 91.82 | 3.863 | 56.4 | 6213044 | 42514 | d__Bacteria;p__Acidobacteriota;c__Acidobacteriae;o__Acidobacteriales;f__SbA1;g__Gp1-AA122 |
| VL3_bin.19 | 98.08 | 0.666 | 50.5 | 2833272 | 46374 | d__Bacteria;p__Proteobacteria;c__Gammaproteobacteria;o__Burkholderiales;f__Methylophilaceae;g__Methylophilus |
| VL3_bin.24 | 98.85 | 1.603 | 63.7 | 4456091 | 69423 | d__Bacteria;p__Proteobacteria;c__Gammaproteobacteria;o__Xanthomonadales;f__Rhodanobacteraceae;g__Rhodanobacter |
| VL3_bin.28 | 92.57 | 2.237 | 59.4 | 4214877 | 12436 | d__Bacteria;p__Acidobacteriota;c__Acidobacteriae;o__Acidobacteriales;f__SbA1 |
| VL3_bin.32 | 94.32 | 3.908 | 64.1 | 4117315 | 12219 | d__Bacteria;p__Actinobacteriota;c__Actinomycetia;o__Actinomycetales;f__Micrococcaceae;g__Arthrobacter_K;s__Arthrobacter_K sp001549895 |
| VL4_bin.15 | 92.3 | 2.849 | 60.9 | 3695304 | 17399 | d__Bacteria;p__Acidobacteriota;c__Acidobacteriae;o__Acidobacteriales;f__CAINCZ01 |
| VL4_bin.22 | 93.81 | 4.433 | 44.1 | 4801987 | 15314 | d__Bacteria;p__Bacteroidota;c__Bacteroidia;o__Chitinophagales;f__Chitinophagaceae;g__UTBCD1 |
| VL4_bin.25 | 95.65 | 4.579 | 64.3 | 2747047 | 41845 | d__Bacteria;p__Proteobacteria;c__Gammaproteobacteria;o__REEB76;f__REEB76 |
| VL5_bin.1 | 93.09 | 3.466 | 57.1 | 5165796 | 8571 | d__Bacteria;p__Acidobacteriota;c__Blastocatellia;o__UBA7656;f__UBA7656;g__JADGNW01 |
| VL5_bin.10 | 96.29 | 3.2 | 64.1 | 2782785 | 96946 | d__Bacteria;p__Proteobacteria;c__Gammaproteobacteria;o__REEB76;f__REEB76 |
| VL5_bin.14 | 93.88 | 1.818 | 51.3 | 5193654 | 23418 | d__Bacteria;p__Chloroflexota;c__Anaerolineae;o__Anaerolineales;f__EnvOPS12;g__UBA12294 |
| VL5_bin.15 | 93.55 | 2.847 | 69.3 | 3746565 | 6927 | d__Bacteria;p__Gemmatimonadota;c__Gemmatimonadetes;o__Gemmatimonadales;f__GWC2-71-9;g__40CM-2-70-7 |
| VL5_bin.17 | 98.64 | 4.487 | 55.2 | 7968439 | 79842 | d__Bacteria;p__Verrucomicrobiota;c__Verrucomicrobiae;o__Pedosphaerales;f__UBA8199;g__PSRM01 |
| VL5_bin.18 | 95.27 | 1.053 | 54.6 | 3285988 | 30182 | d__Bacteria;p__Verrucomicrobiota;c__Verrucomicrobiae;o__Chthoniobacterales;f__UBA10450;g__Udaeobacter |
| VL5_bin.19 | 97.45 | 1.97 | 44.2 | 4913445 | 44793 | d__Bacteria;p__Bacteroidota;c__Bacteroidia;o__Chitinophagales;f__Chitinophagaceae;g__UTBCD1 |
| VL5_bin.28 | 95.97 | 2.984 | 64.9 | 2797302 | 64696 | d__Bacteria;p__Proteobacteria;c__Gammaproteobacteria;o__REEB76;f__REEB76 |
| VL5_bin.30 | 93.68 | 2.564 | 60.9 | 3907871 | 14973 | d__Bacteria;p__Acidobacteriota;c__Acidobacteriae;o__Acidobacteriales;f__CAINCZ01 |
| VL5_bin.38 | 100 | 0.106 | 50.0 | 3178404 | 137597 | d__Bacteria;p__Proteobacteria;c__Gammaproteobacteria;o__Burkholderiales;f__Methylophilaceae;g__Methylophilus |
| VL6_bin.3 | 90.35 | 3.296 | 67.5 | 3251766 | 9392 | d__Bacteria;p__Gemmatimonadota;c__Gemmatimonadetes;o__Gemmatimonadales;f__GWC2-71-9;g__AG41 |
| VL6_bin.4 | 96.52 | 3.418 | 49.1 | 4381755 | 31679 | d__Bacteria;p__Acidobacteriota;c__HRBIN11 |
| VL6_bin.17 | 91.87 | 2.932 | 68.3 | 2563214 | 16493 | d__Bacteria;p__Chloroflexota;c__Limnocylindria;o__P2-11E;f__P2-11E;g__3-1-20CM-2-70-9 |
| VH1_bin.6 | 90.42 | 2.142 | 63.8 | 3008935 | 11576 | d__Bacteria;p__Proteobacteria;c__Alphaproteobacteria;o__Rhizobiales;f__Xanthobacteraceae;g__Pseudolabrys |
| VH1_bin.9 | 91.79 | 2.847 | 68.3 | 3033788 | 5413 | d__Bacteria;p__Gemmatimonadota;c__Gemmatimonadetes;o__Gemmatimonadales;f__GWC2-71-9 |
| VH1_bin.11 | 92.72 | 4.09 | 59.1 | 4551570 | 17463 | d__Bacteria;p__Chloroflexota;c__Anaerolineae;o__Anaerolineales;f__EnvOPS12;g__UBA5195 |
| VH2_bin.8 | 95.72 | 3.418 | 69.6 | 3008170 | 12656 | d__Bacteria;p__Actinobacteriota;c__UBA4738;o__UBA4738;f__HRBIN12;g__DSRY01 |
| VH2_bin.11 | 97.4 | 3.796 | 65.7 | 5554993 | 16874 | d__Bacteria;p__Gemmatimonadota;c__Gemmatimonadetes;o__Gemmatimonadales;f__Gemmatimonadaceae;g__AG2 |
| VH3_bin.6 | 90.74 | 4.415 | 69.8 | 2497611 | 8039 | d__Bacteria;p__Actinobacteriota;c__UBA4738;o__UBA4738;f__HRBIN12;g__DSRY01 |
| VH3_bin.7 | 93.39 | 4.605 | 43.6 | 5971244 | 6550 | d__Bacteria;p__Acidobacteriota |
| VH3_bin.10 | 97.25 | 2.197 | 65.6 | 6251608 | 16108 | d__Bacteria;p__Gemmatimonadota;c__Gemmatimonadetes;o__Gemmatimonadales;f__Gemmatimonadaceae;g__AG2 |
| VH4_bin.1 | 92.89 | 2.454 | 60.6 | 3836482 | 31698 | d__Bacteria;p__Proteobacteria;c__Alphaproteobacteria;o__Rhizobiales;f__Xanthobacteraceae;g__Pseudolabrys |
| VH4_bin.4 | 92.86 | 4.354 | 70.9 | 5496416 | 13430 | d__Bacteria;p__Myxococcota;c__UBA9160;o__UBA9160;f__SMWR01 |
| VH4_bin.12 | 91.88 | 3.653 | 63.7 | 2698942 | 24433 | d__Bacteria;p__Proteobacteria;c__Alphaproteobacteria;o__Rhizobiales;f__Methyloligellaceae;g__Methyloceanibacter |
| VH4_bin.13 | 96.7 | 4.795 | 68.4 | 4848220 | 8875 | d__Bacteria;p__Gemmatimonadota;c__Gemmatimonadetes;o__Gemmatimonadales;f__GWC2-71-9 |
| VH4_bin.15 | 93.72 | 2.97 | 63.4 | 4702835 | 12544 | d__Bacteria;p__Chloroflexota;c__Ktedonobacteria;o__Ktedonobacterales;f__JACDGC01 |
| VH4_bin.17 | 92.98 | 0.493 | 37.6 | 2910991 | 18062 | d__Bacteria;p__Firmicutes;c__Bacilli;o__Bacillales_A;f__Planococcaceae;g__Paenisporosarcina |
| VH4_bin.24 | 95.88 | 3.751 | 43.8 | 6236366 | 49844 | d__Bacteria;p__Acidobacteriota;c__HRBIN11 |
| VH4_bin.29 | 96.7 | 3.846 | 65.8 | 6333494 | 14864 | d__Bacteria;p__Gemmatimonadota;c__Gemmatimonadetes;o__Gemmatimonadales;f__Gemmatimonadaceae;g__AG2 |
| VH4_bin.31 | 92.07 | 3.387 | 73.5 | 5225372 | 13541 | d__Bacteria;p__Myxococcota;c__Myxococcia;o__Myxococcales;f__Anaeromyxobacteraceae;g__Anaeromyxobacter |
| VH5_bin.11 | 93.02 | 2.349 | 67.9 | 4679825 | 32570 | d__Bacteria;p__Proteobacteria;c__Gammaproteobacteria;o__Burkholderiales;f__SG8-39;g__SCGC-AG-212-J23 |
| VH5_bin.18 | 94.74 | 1.025 | 58.5 | 5201986 | 25712 | d__Bacteria;p__Acidobacteriota;c__Acidobacteriae;o__Acidobacteriales;f__SbA1 |
| VH5_bin.19 | 95.24 | 2.896 | 43.6 | 5829346 | 26704 | d__Bacteria;p__Acidobacteriota;c__HRBIN11 |
