# Supplementary material for: Antimony efflux underpins phosphorus cycling and resistance of phosphate-solubilizing bacteria in mining soils
Source: ISME J. 2023 Jun 3;17(8):1278–89. doi: 10.1038/s41396-023-01445-6 (PMC10356851; doi:10.1038/s41396-023-01445-6)
Supplement: Supplementary file 4 — Supplementary table S3 [file 41396_2023_1445_MOESM4_ESM.docx]

**Table S3** Overall functional traits of three representative MAGs.

| Category | Function | Gene abbreviation | Gene name | Hmm file | Corresponding KO | Reaction | Substrate | Product | Hmm detecting threshold | HH2_bin.11 | | HH3_bin.6 | | VL4_bin.10 | |
| --- | --- | --- | --- | --- | --- | --- | --- | --- | --- | --- | --- | --- | --- | --- | --- |
| Thermophilic specific | Thermophilic specific | rgy | reverse gyrase | TIGR01054.hmm | K03170 | Thermophilic specific | N/A | N/A | 469.70\|full | Absent | 0 | Absent | 0 | Absent | 0 |
| Amino acid utilization | 4-aminobutyrate aminotransferase and related aminotransferases | 4-aminobutyrate aminotransferase and related aminotransferases | 4-aminobutyrate aminotransferase and related aminotransferases | K00823.hmm, K07250.hmm, K13524.hmm, K14268.hmm, K03918.hmm | K00823, K07250, K13524, K14268, K03918 | 4-aminobutanoate + 2-oxoglutarate = succinate semialdehyde + L-glutamate [RN:R01648] | 4-aminobutanoate; 2-oxoglutarate | succinate semialdehyde; L-glutamate | 609.33\|domain, 639.10\|domain, 436.80\|full, 825.23\|full, 369.10\|full | Present | 3 | Absent | 0 | Absent | 0 |
| Amino acid utilization | Aminotransferase class I and II | aminotransferase class I and II | aminotransferase class I and II | K05825.hmm | K05825 | L-2-aminoadipate + 2-oxoglutarate = 2-oxoadipate + L-glutamate [RN:R01939] | L-2-aminoadipate; 2-oxoglutarate | 2-oxoadipate; L-glutamate | 392.87\|domain | Present | 1 | Present | 1 | Absent | 0 |
| Amino acid utilization | Phosphoserine aminotransferase | phosphoserine aminotransferase | phosphoserine aminotransferase | K00831.hmm | K00831 | O-phospho-L-serine + 2-oxoglutarate = 3-phosphooxypyruvate + L-glutamate [RN:R04173]; 4-phosphooxy-L-threonine + 2-oxoglutarate = (3R)-3-hydroxy-2-oxo-4-phosphooxybutanoate + L-glutamate [RN:R05085] | O-phospho-L-serine; 2-oxoglutarate; 4-phosphooxy-L-threonine | 3-phosphooxypyruvate; L-glutamate; (3R)-3-hydroxy-2-oxo-4-phosphooxybutanoate | 137.97\|full | Present | 1 | Absent | 0 | Present | 1 |
| Amino acid utilization | Ornithine/acetylornithine aminotransferase | ornithine/acetylornithine aminotransferase | ornithine/acetylornithine aminotransferase | K00819.hmm, K00821.hmm, K05830.hmm, K00840.hmm | K00819, K00821, K05830, K00840 | L-ornithine + a 2-oxo carboxylate = L-glutamate 5-semialdehyde + an L-amino acid [RN:R01343] | L-ornithine; 2-oxo carboxylate | L-glutamate 5-semialdehyde; L-amino acid | 490.03\|full, 483.83\|domain, 505.00\|domain, 704.00\|domain | Present | 1 | Absent | 0 | Present | 2 |
| Amino acid utilization | Branched-chain amino acid aminotransferase/4-amino-4-deoxychorismate lyase | branched-chain amino acid aminotransferase/4-amino-4-deoxychorismate lyase | branched-chain amino acid aminotransferase/4-amino-4-deoxychorismate lyase | K00826.hmm, K02619.hmm, K03342.hmm | K00826, K02619, K03342 | L-leucine + 2-oxoglutarate = 4-methyl-2-oxopentanoate + L-glutamate [RN:R01090] | L-leucine; 2-oxoglutarate | 4-methyl-2-oxopentanoate; L-glutamate | 188.77\|full, 187.00\|full, 522.93\|full | Present | 2 | Present | 1 | Present | 2 |
| Amino acid utilization | Aspartate/tyrosine/aromatic aminotransferase | aspartate/tyrosine/aromatic aminotransferase | aspartate/tyrosine/aromatic aminotransferase | K00812.hmm, K00813.hmm, K11358.hmm, K00832.hmm | K00812, K00813, K11358, K00832 | L-aspartate + 2-oxoglutarate = oxaloacetate + L-glutamate [RN:R00355] | L-aspartate; 2-oxoglutarate | oxaloacetate; L-glutamate | 464.33\|full, 530.40\|full, 342.60\|full, 553.10\|full | Present | 1 | Absent | 0 | Present | 1 |
| Amino acid utilization | Histidinol-phosphate/aromatic aminotransferase | histidinol-phosphate/aromatic aminotransferase | histidinol-phosphate/aromatic aminotransferase | K00817.hmm | K00817 | L-histidinol phosphate + 2-oxoglutarate = 3-(imidazol-4-yl)-2-oxopropyl phosphate + L-glutamate [RN:R03243] | L-histidinol phosphate; 2-oxoglutarate | 3-(imidazol-4-yl)-2-oxopropyl phosphate; L-glutamate | 289.63\|full | Present | 3 | Present | 1 | Present | 1 |
| Amino acid utilization | Serine-pyruvate aminotransferase/archaeal aspartate aminotransferase | serine-pyruvate aminotransferase/archaeal aspartate aminotransferase | serine-pyruvate aminotransferase/archaeal aspartate aminotransferase | K00830.hmm | K00830 | L-alanine + glyoxylate = pyruvate + glycine [RN:R00369] | L-alanine; glyoxylate | pyruvate; glycine | 418.23\|domain | Present | 1 | Absent | 0 | Present | 1 |
| Ethanol fermentation | Acetate => Acetaldehyde | acetate => acetaldehyde | acetate => acetaldehyde | K00129.hmm, K00138.hmm | K00129, K00138 | an aldehyde + NAD(P)+ + H2O = a carboxylate + NAD(P)H + H+ [RN:R00538 R00634] | aldehyde; NAD+; NADP+; H2O | carboxylate; NADH; NADPH; H+ | 764.63\|full, 807.77\|domain | Absent | 0 | Absent | 0 | Absent | 0 |
| Ethanol fermentation | Acetaldehyde => Ethanol | acetaldehyde => ethanol | acetaldehyde => ethanol | K13954.hmm, K00001.hmm, K04072.hmm, K00114.hmm, K00002.hmm, K04022.hmm, K22473.hmm | K13954, K00001, K04072, K00114, K00002, K04022, K22473 | a primary alcohol + NAD+ = an aldehyde + NADH + H+ [RN:R00623]; a secondary alcohol + NAD+ = a ketone + NADH + H+ [RN:R00624] | primary alcohol; NAD+; secondary alcohol | aldehyde; NADH; H+; ketone | 504.00\|domain, 336.20\|domain, 644.33\|full, 652.23\|domain, 406.57\|full, 471.53\|full, 1290.63\|full | Present | 1 | Absent | 0 | Present | 2 |
| Fatty acid degradation | Fatty acid degradation | acyl-CoA dehydrogenase | acyl-CoA dehydrogenase | K00249.hmm, K00255.hmm, K06445.hmm, K06446.hmm, K09456.hmm, K09478.hmm, K11410.hmm, K15980.hmm, K18244.hmm | K00249, K00255, K06445, K06446, K09456, K09478, K11410, K15980, K18244 | a acyl-CoA + electron-transfer flavoprotein = a trans-2,3-dehydroacyl-CoA + reduced electron-transfer flavoprotein [RN:R00392] | acyl-CoA; electron-transfer flavoprotein | trans-2,3-dehydroacyl-CoA; reduced electron-transfer flavoprotein | 318.80\|domain, 575.20\|full, 427.00\|full, 490.30\|domain, 461.93\|full, 500.07\|full, 510.67\|domain, 782.47\|full, 576.53\|domain | Present | 3 | Present | 6 | Present | 6 |
| Aromatics degradation | Protocatechuate/Catechol degradation | catA | catechol 1,2-dioxygenase | K03381.hmm | K03381 | catechol + O2 = cis,cis-muconate [RN:R00817] | catechol; O2 | cis,cis-muconate | 343.50\|domain | Absent | 0 | Absent | 0 | Absent | 0 |
| Aromatics degradation | Phenol => Benzoyl-CoA | ubiX | flavin prenyltransferase | K03186.hmm | K03186 | dimethylallyl phosphate + FMNH2 = prenylated FMNH2 + phosphate [RN:R11225] | dimethylallyl phosphate; FMNH2 | prenylated FMNH2; phosphate | 125.70\|full | Present | 1 | Present | 1 | Present | 1 |
| Aromatics degradation | Phenol => Benzoyl-CoA | bsdC | vanillate/4-hydroxybenzoate decarboxylase subunit C | K01612.hmm | K01612 | 4-hydroxybenzoate = phenol + CO2 [RN:R01238] | 4-hydroxybenzoate | phenol; CO2 | 574.80\|full | Absent | 0 | Absent | 0 | Absent | 0 |
| Aromatics degradation | Benzoyl-CoA reduction | bcrC | benzoyl-CoA reductase subunit C | K04112.hmm | K04112 | cyclohexa-1,5-diene-1-carbonyl-CoA + oxidized ferredoxin + 2 ADP + 2 phosphate = benzoyl-CoA + reduced ferredoxin + 2 ATP + 2 H2O [RN:R02451] | cyclohexa-1,5-diene-1-carbonyl-CoA; oxidized ferredoxin; ADP; phosphate | benzoyl-CoA; reduced ferredoxin; ATP; H2O | 370.00\|full | Absent | 0 | Absent | 0 | Absent | 0 |
| Aromatics degradation | Benzoyl-CoA reduction | bcrB | benzoyl-CoA reductase subunit B | K04113.hmm | K04113 | cyclohexa-1,5-diene-1-carbonyl-CoA + oxidized ferredoxin + 2 ADP + 2 phosphate = benzoyl-CoA + reduced ferredoxin + 2 ATP + 2 H2O [RN:R02451] | cyclohexa-1,5-diene-1-carbonyl-CoA; oxidized ferredoxin; ADP; phosphate | benzoyl-CoA; reduced ferredoxin; ATP; H2O | 317.33\|full | Absent | 0 | Absent | 0 | Absent | 0 |
| Aromatics degradation | Benzoyl-CoA reduction | bcrA | benzoyl-CoA reductase subunit A | K04114.hmm | K04114 | cyclohexa-1,5-diene-1-carbonyl-CoA + oxidized ferredoxin + 2 ADP + 2 phosphate = benzoyl-CoA + reduced ferredoxin + 2 ATP + 2 H2O [RN:R02451] | cyclohexa-1,5-diene-1-carbonyl-CoA; oxidized ferredoxin; ADP; phosphate | benzoyl-CoA; reduced ferredoxin; ATP; H2O | 396.30\|full | Absent | 0 | Absent | 0 | Absent | 0 |
| Aromatics degradation | Benzoyl-CoA reduction | bcrD | benzoyl-CoA reductase subunit D | K04115.hmm | K04115 | cyclohexa-1,5-diene-1-carbonyl-CoA + oxidized ferredoxin + 2 ADP + 2 phosphate = benzoyl-CoA + reduced ferredoxin + 2 ATP + 2 H2O [RN:R02451] | cyclohexa-1,5-diene-1-carbonyl-CoA; oxidized ferredoxin; ADP; phosphate | benzoyl-CoA; reduced ferredoxin; ATP; H2O | 296.30\|full | Absent | 0 | Absent | 0 | Absent | 0 |
| Complex carbon degradation | Cellulose degrading | cellobiosidase | cellobiosidase | K19668.hmm | K19668 | Hydrolysis of (1->4)-beta-D-glucosidic linkages in cellulose and cellotetraose, releasing cellobiose from the non-reducing ends of the chains | N/A | N/A | 225.47\|domain | Absent | 0 | Absent | 0 | Absent | 0 |
| Complex carbon degradation | Cellulose degrading | cellulase | cellulase | K01779.hmm | K01779 | L-aspartate = D-aspartate [RN:R00491] | L-aspartate | D-aspartate | 90.43\|full | Absent | 0 | Absent | 0 | Absent | 0 |
| Complex carbon degradation | Cellulose degrading | beta-glucosidase | beta-glucosidase | K01188.hmm, K05349.hmm, K05350.hmm | K01188, K05349, K05350 | Hydrolysis of terminal, non-reducing beta-D-glucosyl residues with release of beta-D-glucose | N/A | N/A | 695.13\|domain, 314.90\|full, 584.90\|domain | Present | 2 | Absent | 0 | Present | 1 |
| Complex carbon degradation | Hemicullulose debranching | arabinosidase | arabinosidase | K01209.hmm, K15921.hmm | K01209, K15921 | Hydrolysis of terminal non-reducing alpha-L-arabinofuranoside residues in alpha-L-arabinosides. | N/A | N/A | 96.47\|full, 225.70\|domain | Absent | 0 | Absent | 0 | Absent | 0 |
| Complex carbon degradation | Hemicullulose debranching | beta-glucuronidase | beta-glucuronidase | K01195.hmm | K01195 | a beta-D-glucuronoside + H2O = D-glucuronate + an alcohol [RN:R01478] | beta-D-glucuronoside; H2O | D-glucuronate; alcohol | 403.53\|full | Absent | 0 | Absent | 0 | Absent | 0 |
| Complex carbon degradation | Hemicullulose debranching | alpha-L-rhamnosidase | alpha-L-rhamnosidase | K05989.hmm | K05989 | Hydrolysis of terminal non-reducing alpha-L-rhamnose residues in alpha-L-rhamnosides | N/A | N/A | 173.10\|domain | Absent | 0 | Absent | 0 | Absent | 0 |
| Complex carbon degradation | Endohemicellulases | mannan endo-1,4-beta-mannosidase | mannan endo-1,4-beta-mannosidase | K01218.hmm, K19355.hmm | K01218, K19355 | Random hydrolysis of (1->4)-beta-D-mannosidic linkages in mannans, galactomannans and glucomannans [RN:R08612] | N/A | N/A | 78.93\|domain, 100.53\|full | Absent | 0 | Absent | 0 | Absent | 0 |
| Complex carbon degradation | Endohemicellulases | alpha-D-xyloside xylohydrolase | alpha-D-xyloside xylohydrolase | K01811.hmm | K01811 | Hydrolysis of terminal, non-reducing alpha-D-xylose residues with release of alpha-D-xylose. | N/A | N/A | 621.53\|domain | Absent | 0 | Absent | 0 | Absent | 0 |
| Complex carbon degradation | Other oligosaccharide degrading | beta-xylosidase | beta-xylosidase | K01198.hmm | K01198 | Hydrolysis of (1->4)-beta-D-xylans, to remove successive D-xylose residues from the non-reducing termini | N/A | N/A | 184.60\|domain | Absent | 0 | Absent | 0 | Absent | 0 |
| Complex carbon degradation | Other oligosaccharide degrading | beta-mannosidase | beta-mannosidase | K01192.hmm | K01192 | Hydrolysis of terminal, non-reducing beta-D-mannose residues in beta-D-mannosides [RN:R08613] | N/A | N/A | 406.10\|full | Present | 1 | Absent | 0 | Absent | 0 |
| Complex carbon degradation | Other oligosaccharide degrading | beta-galactosidase | beta-galactosidase | K01190.hmm, K12111.hmm, K12308.hmm | K01190, K12111, K12308 | Hydrolysis of terminal non-reducing beta-D-galactose residues in beta-D-galactosides | N/A | N/A | 382.13\|full, 1274.03\|domain, 225.67\|domain | Absent | 0 | Absent | 0 | Absent | 0 |
| Complex carbon degradation | Amylolytic enzymes | alpha-amylase | alpha-amylase | K01176.hmm, K05343.hmm, K07405.hmm | K01176, K05343, K07405 | Endohydrolysis of (1->4)-alpha-D-glucosidic linkages in polysaccharides containing three or more (1->4)-alpha-linked D-glucose units | N/A | N/A | 252.60\|domain, 495.17\|domain, 344.03\|full | Present | 1 | Present | 1 | Present | 1 |
| Complex carbon degradation | Amylolytic enzymes | glucoamylase | glucoamylase | K01178.hmm | K01178 | Hydrolysis of terminal (1->4)-linked alpha-D-glucose residues successively from non-reducing ends of the chains with release of beta-D-glucose | N/A | N/A | 152.77\|full | Present | 1 | Absent | 0 | Absent | 0 |
| Complex carbon degradation | Amylolytic enzymes | pullulanase | pullulanase | K01200.hmm | K01200 | Hydrolysis of (1->6)-alpha-D-glucosidic linkages in pullulan, amylopectin and glycogen, and in the alpha- and beta-limit dextrins of amylopectin and glycogen | N/A | N/A | 390.57\|domain | Absent | 0 | Absent | 0 | Absent | 0 |
| Complex carbon degradation | Amylolytic enzymes | isoamylase | isoamylase | K01214.hmm | K01214 | Hydrolysis of (1->6)-alpha-D-glucosidic branch linkages in glycogen, amylopectin and their beta-limit dextrins | N/A | N/A | 962.67\|full | Absent | 0 | Present | 1 | Absent | 0 |
| Complex carbon degradation | Chitin degrading | chitiniase | chitiniase | K01183.hmm, K13381.hmm | K01183, K13381 | Random endo-hydrolysis of N-acetyl-beta-D-glucosaminide (1->4)-beta-linkages in chitin and chitodextrins | N/A | N/A | 121.47\|full, 339.03\|domain | Absent | 0 | Absent | 0 | Absent | 0 |
| Complex carbon degradation | Chitin degrading | hexosaminidase | hexosaminidase | K01207.hmm, K12373.hmm, K14459.hmm | K01207, K12373, K14459 | Hydrolysis of terminal non-reducing N-acetyl-D-hexosamine residues in N-acetyl-beta-D-hexosaminides | N/A | N/A | 253.67\|domain, 86.50\|full, 200.80\|full | Present | 2 | Present | 1 | Present | 1 |
| Fermentation | Pyruvate oxidation | porA | pyruvate ferredoxin oxidoreductase alpha subunit | K00169.hmm | K00169 | pyruvate + CoA + 2 oxidized ferredoxin = acetyl-CoA + CO2 + 2 reduced ferredoxin + 2 H+ [RN:R01196] | pyruvate; CoA; oxidized ferredoxin | acetyl-CoA; CO2; reduced ferredoxin; H+ | 299.20\|full | Present | 1 | Absent | 0 | Absent | 0 |
| Fermentation | Alcohol utilization | adh | alcohol dehydrogenase | K00001.hmm | K00001 | a primary alcohol + NAD+ = an aldehyde + NADH + H+ [RN:R00623]; a secondary alcohol + NAD+ = a ketone + NADH + H+ [RN:R00624] | primary alcohol; NAD+; secondary alcohol | aldehyde; NADH; H+; ketone | 336.20\|domain | Absent | 0 | Absent | 0 | Absent | 0 |
| Fermentation | Lactate utilization | ldh | L-lactate dehydrogenase | K00016.hmm | K00016 | (S)-lactate + NAD+ = pyruvate + NADH + H+ [RN:R00703] | (S)-lactate; NAD+ | pyruvate; NADH; H+ | 317.10\|full | Absent | 0 | Absent | 0 | Absent | 0 |
| Fermentation | Acetogenesis | acdA | acetyl coenzyme A synthetase (ADP forming), alpha domain | TIGR02717.hmm | K01905 | ATP + acetate + CoA = ADP + phosphate + acetyl-CoA [RN:R00229] | ATP; acetate; CoA | ADP; phosphate; acetyl-CoA | 463.00\|full | Absent | 0 | Present | 1 | Present | 1 |
| Fermentation | Acetogenesis | ack | acetate kinase | TIGR00016.hmm | K00925 | ATP + acetate = ADP + acetyl phosphate [RN:R00315] | ATP; acetate | ADP; acetyl phosphate | 146.10\|full | Absent | 0 | Absent | 0 | Absent | 0 |
| Fermentation | Acetogenesis | pta | phosphate acetyltransferase | TIGR00651.hmm | K00625 | acetyl-CoA + phosphate = CoA + acetyl phosphate [RN:R00230] | acetyl-CoA; phosphate | CoA; acetyl phosphate | 205.40\|full | Absent | 0 | Absent | 0 | Absent | 0 |
| Fermentation | Acetate to acetyl-CoA | acs | acetyl-CoA synthetase | TIGR02188.hmm | K01895 | ATP + acetate + CoA = AMP + diphosphate + acetyl-CoA [RN:R00235] | ATP; acetate; CoA | AMP; diphosphate; acetyl-CoA | 684.65\|full | Present | 2 | Present | 1 | Present | 1 |
| Fermentation | Pyruvate <=> acetyl-CoA + formate | pflD | formate C-acetyltransferase | K00656.hmm | K00656 | acetyl-CoA + formate = CoA + pyruvate [RN:R00212] | acetyl-CoA; formate | CoA; pyruvate | 336.47\|full | Absent | 0 | Absent | 0 | Absent | 0 |
| C1 metabolism | Methanol oxidation | mxaF | methanol dehydrogenase (cytochrome c) subunit 1 | methanol_dehydrogenase_pqq_xoxF_mxaF.hmm | K14028 | a primary alcohol + 2 ferricytochrome cL = an aldehyde + 2 ferrocytochrome cL + 2 H+ [RN:R10713] | primary alcohol; ferricytochrome cL | aldehyde; ferrocytochrome cL; H+ | 400\|full | Present | 1 | Absent | 0 | Present | 4 |
| C1 metabolism | Methanol oxidation | mdh | methanol dehydrogenase | TIGR04266.hmm | K00093 | methanol + NAD+ = formaldehyde + NADH + H+ [RN:R00605] | methanol; NAD+ | formaldehyde; NADH; H+ | 400.00\|full | Absent | 0 | Absent | 0 | Absent | 0 |
| C1 metabolism | Methyl amine -> formaldehyde | mauA | methylamine dehydrogenase light chain | TIGR02659.hmm | K15228 | methylamine + H2O + 2 amicyanin = formaldehyde + NH3 + 2 reduced amicyanin [RN:R00606] | methylamine; H2O; amicyanin | formaldehyde; NH3; reduced amicyanin | 85.80\|full | Absent | 0 | Absent | 0 | Absent | 0 |
| C1 metabolism | Methyl amine -> formaldehyde | mauB | methylamine dehydrogenase heavy chain | TIGR02658.hmm | K15229 | methylamine + H2O + 2 amicyanin = formaldehyde + NH3 + 2 reduced amicyanin [RN:R00606] | methylamine; H2O; amicyanin | formaldehyde; NH3; reduced amicyanin | 90.50\|full | Absent | 0 | Absent | 0 | Absent | 0 |
| C1 metabolism | Formaldehyde oxidation | fdhA | glutathione-independent formaldehyde dehydrogenase | TIGR02819.hmm | K00148 | formaldehyde + NAD+ + H2O = formate + NADH + 2 H+ [RN:R00604] | formaldehyde; NAD+; H2O | formate; NADH; H+ | 425.15\|full | Absent | 0 | Absent | 0 | Absent | 0 |
| C1 metabolism | Formaldehyde oxidation | fghA | S-formylglutathione hydrolase | TIGR02821.hmm | K01070 | S-formylglutathione + H2O = glutathione + formate [RN:R00527] | S-formylglutathione; H2O | glutathione; formate | 131.95\|full | Absent | 0 | Absent | 0 | Present | 1 |
| C1 metabolism | Formaldehyde oxidation | frmA | S-(hydroxymethyl)glutathione dehydrogenase / alcohol dehydrogenase | TIGR02818.hmm | K00121 | S-(hydroxymethyl)glutathione + NAD(P)+ = S-formylglutathione + NAD(P)H + H+ [RN:R06983 R07140] | S-(hydroxymethyl)glutathione; NAD+; NADP+ | S-formylglutathione; NADH; NADPH; H+ | 509.55\|full | Absent | 0 | Absent | 0 | Present | 1 |
| C1 metabolism | Formaldehyde oxidation | mycoS_dep_FDH | S-(hydroxymethyl)mycothiol dehydrogenase | TIGR03451.hmm | K00153 | S-(hydroxymethyl)mycothiol + NAD+ = S-formylmycothiol + NADH + H+ [RN:R09129] | S-(hydroxymethyl)mycothiol; NAD+ | S-formylmycothiol; NADH; H+ | 512.15\|full | Absent | 0 | Absent | 0 | Absent | 0 |
| C1 metabolism | Formaldehyde oxidation | fae | 5,6,7,8-tetrahydromethanopterin hydro-lyase | TIGR03126.hmm | K10713 | 5,6,7,8-tetrahydromethanopterin + formaldehyde = 5,10-methylenetetrahydromethanopterin + H2O [RN:R08058] | 5,6,7,8-tetrahydromethanopterin; formaldehyde | 5,10-methylenetetrahydromethanopterin; H2O | 31.15\|full | Absent | 0 | Absent | 0 | Absent | 0 |
| C1 metabolism | Formate oxidation | fdoG | formate dehydrogenase major subunit | K00123.hmm | K00123 | formate + NAD+ = CO2 + NADH [RN:R00519] | formate; NAD+ [CPD:C00003] | CO2; NADH | 841.13\|full | Absent | 0 | Absent | 0 | Present | 2 |
| C1 metabolism | Formate oxidation | fdwB | formate dehydrogenase beta subunit | K22515.hmm | K22515 | formate + NAD+ = CO2 + NADH [RN:R00519] | formate; NAD+ [CPD:C00003] | CO2; NADH | 555.20\|domain | Present | 1 | Absent | 0 | Present | 1 |
| C1 metabolism | Formate oxidation | fdoH | formate dehydrogenase iron-sulfur subunit | K00124.hmm | K00124 | formate + NAD+ = CO2 + NADH [RN:R00519] | formate; NAD+ [CPD:C00003] | CO2; NADH | 169.00\|domain | Present | 3 | Absent | 0 | Absent | 0 |
| C1 metabolism | Formate oxidation | fdhA | formate dehydrogenase (coenzyme F420) alpha subunit | K22516.hmm | K22516 | formate + oxidized coenzyme F420 = CO2 + reduced coenzyme F420 [RN:R10797] | formate; oxidized coenzyme F420 | CO2; reduced coenzyme F420 | 937.40\|full | Absent | 0 | Absent | 0 | Absent | 0 |
| C1 metabolism | Formate oxidation | fdhB | formate dehydrogenase (coenzyme F420) beta subunit | K00125.hmm | K00125 | formate + oxidized coenzyme F420 = CO2 + reduced coenzyme F420 [RN:R10797] | formate; oxidized coenzyme F420 | CO2; reduced coenzyme F420 | 209.97\|full | Absent | 0 | Absent | 0 | Absent | 0 |
| C1 metabolism | Aerobic CO oxidation | coxS | aerobic carbon-monoxide dehydrogenase small subunit | K03518.hmm | K03518 | CO + a quinone + H2O = CO2 + a quinol [RN:R11168] | CO; quinone; H2O | CO2; quinol | 242.00\|domain | Absent | 0 | Present | 1 | Present | 5 |
| C1 metabolism | Aerobic CO oxidation | coxM | aerobic carbon-monoxide dehydrogenase medium subunit | K03519.hmm | K03519 | CO + a quinone + H2O = CO2 + a quinol [RN:R11168] | CO; quinone; H2O | CO2; quinol | 272.50\|domain | Absent | 0 | Absent | 0 | Present | 4 |
| C1 metabolism | Aerobic CO oxidation | coxL | aerobic carbon-monoxide dehydrogenase large subunit | K03520.hmm | K03520 | CO + a quinone + H2O = CO2 + a quinol [RN:R11168] | CO; quinone; H2O | CO2; quinol | 846.90\|full | Absent | 0 | Absent | 0 | Present | 8 |
| Methane metabolism | Methane oxidation - Partculate methane monooxygenase | pmoA | methane/ammonia monooxygenase subunit A | pmoA.hmm | K10944 | methane + quinol + O2 = methanol + quinone + H2O [RN:R09518] | methane; quinol; O2 | methanol; quinone; H2O | 160.90\|full | Absent | 0 | Absent | 0 | Absent | 0 |
| Methane metabolism | Methane oxidation - Partculate methane monooxygenase | pmoB | methane/ammonia monooxygenase subunit B | pmoB.hmm | K10945 | methane + quinol + O2 = methanol + quinone + H2O [RN:R09518] | methane; quinol; O2 | methanol; quinone; H2O | 156.70\|full | Absent | 0 | Absent | 0 | Absent | 0 |
| Methane metabolism | Methane oxidation - Partculate methane monooxygenase | pmoC | methane/ammonia monooxygenase subunit C | pmoC.hmm | K10946 | methane + quinol + O2 = methanol + quinone + H2O [RN:R09518] | methane; quinol; O2 | methanol; quinone; H2O | 136.27\|full | Absent | 0 | Absent | 0 | Absent | 0 |
| Methane metabolism | Methane oxidation - Soluble methane monoxygenase | mmoB | methane monooxygenase regulatory protein B | PF02406.hmm | K16160 | methane + NAD(P)H + H+ + O2 = methanol + NAD(P)+ + H2O [RN:R01142 R01143] | methane; NADH; NADPH; H+; O2 | methanol; NAD+; NADP+; H2O | 10\|full | Absent | 0 | Absent | 0 | Absent | 0 |
| Methane metabolism | Methane oxidation - Soluble methane monoxygenase | mmoD | methane monooxygenase component D | TIGR04550.hmm | K16162 | methane + NAD(P)H + H+ + O2 = methanol + NAD(P)+ + H2O [RN:R01142 R01143] | methane; NADH; NADPH; H+; O2 | methanol; NAD+; NADP+; H2O | 28.00\|full | Absent | 0 | Absent | 0 | Absent | 0 |
| Methane metabolism | Methane production | mcrA | methyl-coenzyme M reductase alpha subunit | TIGR03256.hmm | K00399 | methyl-CoM + CoB = CoM-S-S-CoB + methane [RN:R04541] | methyl-CoM; CoB | CoM-S-S-CoB; methane | 314.45\|full | Absent | 0 | Absent | 0 | Absent | 0 |
| Methane metabolism | Methane production | mcrB | methyl-coenzyme M reductase beta subunit | TIGR03257.hmm | K00401 | methyl-CoM + CoB = CoM-S-S-CoB + methane [RN:R04541] | methyl-CoM; CoB | CoM-S-S-CoB; methane | 173.35\|full | Absent | 0 | Absent | 0 | Absent | 0 |
| Methane metabolism | Methane production | mcrC | methyl-coenzyme M reductase subunit C | TIGR03259.hmm | K03421 | methyl-CoM + CoB = CoM-S-S-CoB + methane [RN:R04541] | methyl-CoM; CoB | CoM-S-S-CoB; methane | 171.60\|full | Absent | 0 | Absent | 0 | Absent | 0 |
| Carbon fixation | CBB cycle - Rubisco | Form I | Form I RuBisCO large subunit | rubisco_form_I.hmm | K01601 | 2 3-phospho-D-glycerate + 2 H+ = D-ribulose 1,5-bisphosphate + CO2 + H2O [RN:R00024] | 3-phospho-D-glycerate; H+ | D-ribulose 1,5-bisphosphate; CO2; H2O | 500\|full | Absent | 0 | Absent | 0 | Absent | 0 |
| Carbon fixation | CBB cycle - Rubisco | Form II | Form II RuBisCO large subunit | rubisco_form_II.hmm | K01601 | 2 3-phospho-D-glycerate + 2 H+ = D-ribulose 1,5-bisphosphate + CO2 + H2O [RN:R00024] | 3-phospho-D-glycerate; H+ | D-ribulose 1,5-bisphosphate; CO2; H2O | 500\|full | Absent | 0 | Absent | 0 | Absent | 0 |
| Carbon fixation | 3 Hydroxypropionate cycle | K14469 | propionyl-CoA synthase | K14469.hmm | K14469 | 3-Hydroxypropionyl-CoA = Propenoyl-CoA + H2O [RN:R03045] | 3-Hydroxypropionyl-CoA | Propenoyl-CoA | 2774.67\|full | Absent | 0 | Absent | 0 | Absent | 0 |
| Carbon fixation | 3 Hydroxypropionate cycle | mcr | malonyl-CoA reductase / 3-hydroxypropionate dehydrogenase (NADP+) | K14468.hmm | K14468 | 3-hydroxypropanoate + NADP+ = malonate semialdehyde + NADPH + H+ [RN:R09289] | 3-hydroxypropanoate; NADP+ | malonate semialdehyde; NADPH; H+ | 1652.50\|full | Absent | 0 | Absent | 0 | Absent | 0 |
| Carbon fixation | 3HP/4HB | K14466 | 4-hydroxybutyrate---CoA ligase (ADP-forming) | K14466.hmm | K14466 | ATP + 4-hydroxybutanoate + CoA = AMP + diphosphate + 4-hydroxybutanoyl-CoA [RN:R09279] | ATP; 4-hydroxybutanoate; CoA | AMP; diphosphate; 4-hydroxybutanoyl-CoA | 914.07\|full | Absent | 0 | Absent | 0 | Absent | 0 |
| Carbon fixation | 3HP/4HB or DC/4-HB | K18861 | 4-hydroxybutyrate---CoA ligase (ADP-forming) | K18861.hmm | K18861 | ATP + 4-hydroxybutanoate + CoA = AMP + diphosphate + 4-hydroxybutanoyl-CoA [RN:R09279] | ATP; 4-hydroxybutanoate; CoA | AMP; diphosphate; 4-hydroxybutanoyl-CoA | 946.67\|full | Absent | 0 | Absent | 0 | Absent | 0 |
| Carbon fixation | DC/4-HB | 4hbl | 4-hydroxybutyrate---CoA ligase (ADP-forming) | K14467.hmm | K14467 | ATP + 4-hydroxybutanoate + CoA = AMP + diphosphate + 4-hydroxybutanoyl-CoA [RN:R09279] | ATP; 4-hydroxybutanoate; CoA | AMP; diphosphate; 4-hydroxybutanoyl-CoA | 1019.33\|domain | Absent | 0 | Absent | 0 | Absent | 0 |
| Carbon fixation | Wood Ljungdahl pathway | cdhD | acetyl-CoA decarbonylase/synthase, CODH/ACS complex subunit delta | K00194.hmm | K00194 | a [methyl-Co(III) corrinoid Fe-S protein] + tetrahydrosarcinapterin = a [Co(I) corrinoid Fe-S protein] + 5-methyltetrahydrosarcinapterin [RN:R10385] | [methyl-Co(III) corrinoid Fe-S protein]; tetrahydrosarcinapterin | [Co(I) corrinoid Fe-S protein]; 5-methyltetrahydrosarcinapterin | 344.50\|full | Absent | 0 | Absent | 0 | Absent | 0 |
| Carbon fixation | Wood Ljungdahl pathway | cdhE | acetyl-CoA decarbonylase/synthase, CODH/ACS complex subunit gamma | K00197.hmm | K00197 | a [methyl-Co(III) corrinoid Fe-S protein] + tetrahydrosarcinapterin = a [Co(I) corrinoid Fe-S protein] + 5-methyltetrahydrosarcinapterin [RN:R10385] | [methyl-Co(III) corrinoid Fe-S protein]; tetrahydrosarcinapterin | [Co(I) corrinoid Fe-S protein]; 5-methyltetrahydrosarcinapterin | 361.33\|full | Absent | 0 | Absent | 0 | Absent | 0 |
| Carbon fixation | Wood Ljungdahl pathway | cooS | anaerobic carbon-monoxide dehydrogenase catalytic subunit | K00198.hmm | K00198 | a [methyl-Co(III) corrinoid Fe-S protein] + tetrahydrosarcinapterin = a [Co(I) corrinoid Fe-S protein] + 5-methyltetrahydrosarcinapterin [RN:R10385] | [methyl-Co(III) corrinoid Fe-S protein]; tetrahydrosarcinapterin | [Co(I) corrinoid Fe-S protein]; 5-methyltetrahydrosarcinapterin | 544.77\|full | Absent | 0 | Absent | 0 | Absent | 0 |
| Carbon fixation | Reverse TCA cycle | aclA | ATP-citrate lyase alpha-subunit | acetate_citrate_lyase_aclA.hmm | K15230 | ADP + phosphate + acetyl-CoA + oxaloacetate = ATP + citrate + CoA [RN:R00352] | ADP; phosphate; acetyl-CoA; oxaloacetate | ATP; citrate; CoA | 215\|full | Absent | 0 | Absent | 0 | Absent | 0 |
| Carbon fixation | Reverse TCA cycle | aclB | ATP-citrate lyase beta-subunit | acetate_citrate_lyase_aclB.hmm | K15231 | ADP + phosphate + acetyl-CoA + oxaloacetate = ATP + citrate + CoA [RN:R00352] | ADP; phosphate; acetyl-CoA; oxaloacetate | ATP; citrate; CoA | 177\|full | Absent | 0 | Absent | 0 | Absent | 0 |
| Nitrogen cycling | Ammonia oxidation | amoA | methane/ammonia monooxygenase subunit A | amoA.hmm | K10944 | NH3 + a reduced acceptor + O2 = NH2OH + an acceptor + H2O [RN:R09519] | NH3; reduced acceptor; O2 | NH2OH; acceptor; H2O | 160.90\|full | Absent | 0 | Absent | 0 | Absent | 0 |
| Nitrogen cycling | Ammonia oxidation | amoB | methane/ammonia monooxygenase subunit B | amoB.hmm | K10945 | NH3 + a reduced acceptor + O2 = NH2OH + an acceptor + H2O [RN:R09519] | NH3; reduced acceptor; O2 | NH2OH; acceptor; H2O | 156.70\|full | Absent | 0 | Absent | 0 | Absent | 0 |
| Nitrogen cycling | Ammonia oxidation | amoC | methane/ammonia monooxygenase subunit C | amoC.hmm | K10946 | NH3 + a reduced acceptor + O2 = NH2OH + an acceptor + H2O [RN:R09519] | NH3; reduced acceptor; O2 | NH2OH; acceptor; H2O | 136.27\|full | Absent | 0 | Absent | 0 | Absent | 0 |
| Nitrogen cycling | N2 fixation | anfD | nitrogenase iron-iron protein, alpha subunit | TIGR01861.hmm | N/A | 8 reduced ferredoxin + 8 H+ + N2 + 16 ATP + 16 H2O = 8 oxidized ferredoxin + H2 + 2 NH3 + 16 ADP + 16 phosphate [RN:R05185] | reduced ferredoxin; H+; N2; ATP; H2O | oxidized ferredoxin; H2; NH3; ADP; phosphate | 873.55\|full | Absent | 0 | Absent | 0 | Absent | 0 |
| Nitrogen cycling | N2 fixation | anfK | nitrogenase iron-iron protein, beta subunit | TIGR02931.hmm | N/A | 8 reduced ferredoxin + 8 H+ + N2 + 16 ATP + 16 H2O = 8 oxidized ferredoxin + H2 + 2 NH3 + 16 ADP + 16 phosphate [RN:R05185] | reduced ferredoxin; H+; N2; ATP; H2O | oxidized ferredoxin; H2; NH3; ADP; phosphate | 853.75\|full | Absent | 0 | Absent | 0 | Absent | 0 |
| Nitrogen cycling | N2 fixation | anfG | nitrogenase iron-iron protein, delta subunit | TIGR02929.hmm | K00531 | 8 reduced ferredoxin + 8 H+ + N2 + 16 ATP + 16 H2O = 8 oxidized ferredoxin + H2 + 2 NH3 + 16 ADP + 16 phosphate [RN:R05185] | reduced ferredoxin; H+; N2; ATP; H2O | oxidized ferredoxin; H2; NH3; ADP; phosphate | 124.50\|full | Absent | 0 | Absent | 0 | Absent | 0 |
| Nitrogen cycling | N2 fixation | nifD | nitrogenase molybdenum-iron protein alpha chain | TIGR01282.hmm | K02586 | 8 reduced ferredoxin + 8 H+ + N2 + 16 ATP + 16 H2O = 8 oxidized ferredoxin + H2 + 2 NH3 + 16 ADP + 16 phosphate [RN:R05185] | reduced ferredoxin; H+; N2; ATP; H2O | oxidized ferredoxin; H2; NH3; ADP; phosphate | 503.05\|full | Absent | 0 | Absent | 0 | Absent | 0 |
| Nitrogen cycling | N2 fixation | nifK | nitrogenase molybdenum-iron protein beta chain | TIGR01286.hmm | K02591 | 8 reduced ferredoxin + 8 H+ + N2 + 16 ATP + 16 H2O = 8 oxidized ferredoxin + H2 + 2 NH3 + 16 ADP + 16 phosphate [RN:R05185] | reduced ferredoxin; H+; N2; ATP; H2O | oxidized ferredoxin; H2; NH3; ADP; phosphate | 414.50\|full | Absent | 0 | Absent | 0 | Absent | 0 |
| Nitrogen cycling | N2 fixation | vnfD | nitrogenase vanadium-iron protein, alpha chain | TIGR01860.hmm | K22896 | 12 reduced ferredoxin + 12 H+ + N2 + 40 ATP + 40 H2O = 12 oxidized ferredoxin + 3 H2 + 2 NH3 + 40 ADP + 40 phosphate [RN:R12084] | reduced ferredoxin; H+; N2; ATP; H2O | oxidized ferredoxin; H2; NH3; ADP; phosphate | 822.85\|full | Absent | 0 | Absent | 0 | Absent | 0 |
| Nitrogen cycling | N2 fixation | vnfK | nitrogenase vanadium-iron protein, beta chain | TIGR02932.hmm | K22897 | 12 reduced ferredoxin + 12 H+ + N2 + 40 ATP + 40 H2O = 12 oxidized ferredoxin + 3 H2 + 2 NH3 + 40 ADP + 40 phosphate [RN:R12084] | reduced ferredoxin; H+; N2; ATP; H2O | oxidized ferredoxin; H2; NH3; ADP; phosphate | 821.85\|full | Absent | 0 | Absent | 0 | Absent | 0 |
| Nitrogen cycling | N2 fixation | vnfG | nitrogenase vanadium-iron protein, delta chain | TIGR02930.hmm | K22898 | 12 reduced ferredoxin + 12 H+ + N2 + 40 ATP + 40 H2O = 12 oxidized ferredoxin + 3 H2 + 2 NH3 + 40 ADP + 40 phosphate [RN:R12084] | reduced ferredoxin; H+; N2; ATP; H2O | oxidized ferredoxin; H2; NH3; ADP; phosphate | 122.35\|full | Absent | 0 | Absent | 0 | Absent | 0 |
| Nitrogen cycling | N2 fixation | nifH | nitrogenase iron protein | TIGR01287.hmm | K02588 | 8 reduced ferredoxin + 8 H+ + N2 + 16 ATP + 16 H2O = 8 oxidized ferredoxin + H2 + 2 NH3 + 16 ADP + 16 phosphate [RN:R05185] | reduced ferredoxin; H+; N2; ATP; H2O | oxidized ferredoxin; H2; NH3; ADP; phosphate | 261.05\|full | Absent | 0 | Absent | 0 | Absent | 0 |
| Nitrogen cycling | Nitrite oxidation | nxrA | nitrate reductase / nitrite oxidoreductase, alpha subunit | nitrite_oxidoreductase_nxrA.hmm | K00370 | nitrate + a quinol = nitrite + a quinone + H2O [RN:R09497] | nitrate; quinol | nitrite; quinone; H2O | 370\|full | Absent | 0 | Absent | 0 | Absent | 0 |
| Nitrogen cycling | Nitrite oxidation | nxrB | nitrate reductase / nitrite oxidoreductase, beta subunit | nitrite_oxidoreductase_nxrB.hmm | K00371 | nitrate + a quinol = nitrite + a quinone + H2O [RN:R09497] | nitrate; quinol | nitrite; quinone; H2O | 252\|full | Absent | 0 | Absent | 0 | Absent | 0 |
| Nitrogen cycling | Nitrate reduction | napA | periplasmic nitrate reductase NapA | TIGR01706.hmm | K02567 | Nitrite + Acceptor + H2O <=> Nitrate + Reduced acceptor | Nitrite; Acceptor; H2O | Nitrate; Reduced acceptor | 472.50\|full | Absent | 0 | Absent | 0 | Absent | 0 |
| Nitrogen cycling | Nitrate reduction | napB | cytochrome c-type protein NapB | PF03892.hmm | K02568 | Nitrite + Acceptor + H2O <=> Nitrate + Reduced acceptor | Nitrite; Acceptor; H2O | Nitrate; Reduced acceptor | 23.9\|full | Present | 1 | Absent | 0 | Absent | 0 |
| Nitrogen cycling | Nitrate reduction | narG | nitrate reductase / nitrite oxidoreductase, alpha subunit | TIGR01580.hmm | K00370 | nitrate + a quinol = nitrite + a quinone + H2O [RN:R09497] | nitrate; quinol | nitrite; quinone; H2O | 600.95\|full | Absent | 0 | Absent | 0 | Present | 1 |
| Nitrogen cycling | Nitrate reduction | narH | nitrate reductase / nitrite oxidoreductase, beta subunit | TIGR01660.hmm | K00371 | nitrate + a quinol = nitrite + a quinone + H2O [RN:R09497] | nitrate; quinol | nitrite; quinone; H2O | 348.00\|full | Absent | 0 | Absent | 0 | Present | 1 |
| Nitrogen cycling | Nitrite reduction to ammonia | nrfH | cytochrome c nitrite reductase small subunit | TIGR03153.hmm | K15876 | NH3 + 2 H2O + 6 ferricytochrome c = nitrite + 6 ferrocytochrome c + 7 H+ [RN:R05712] | NH3; H2O; ferricytochrome c | nitrite; ferrocytochrome c; H+ | 75.30\|full | Absent | 0 | Absent | 0 | Absent | 0 |
| Nitrogen cycling | Nitrite reduction to ammonia | nrfA | nitrite reductase (cytochrome c-552) | PF02335.hmm | K03385 | NH3 + 2 H2O + 6 ferricytochrome c = nitrite + 6 ferrocytochrome c + 7 H+ [RN:R05712] | NH3; H2O; ferricytochrome c | nitrite; ferrocytochrome c; H+ | 57\|full | Absent | 0 | Absent | 0 | Absent | 0 |
| Nitrogen cycling | Nitrite reduction to ammonia | nrfD | cytochrome c nitrite reductase, NrfD subunit | TIGR03148.hmm | K04015 | NH3 + 2 H2O + 6 ferricytochrome c = nitrite + 6 ferrocytochrome c + 7 H+ [RN:R05712] | NH3; H2O; ferricytochrome c | nitrite; ferrocytochrome c; H+ | 300.15\|full | Absent | 0 | Absent | 0 | Absent | 0 |
| Nitrogen cycling | Nitrite reduction to ammonia | nirB | nitrite reductase (NADH) large subunit | TIGR02374.hmm | K00362 | NH3 + 3 NAD+ + 2 H2O = nitrite + 3 NADH + 5 H+ [RN:R00787] | NH3; NAD+; H2O | nitrite; NADH; H+ | 442.75\|full | Absent | 0 | Absent | 0 | Absent | 0 |
| Nitrogen cycling | Nitrite reduction to ammonia | nirD | nitrite reductase (NADH) small subunit | TIGR02378.hmm | K00363 | NH3 + 3 NAD+ + 2 H2O = nitrite + 3 NADH + 5 H+ [RN:R00787] | NH3; NAD+; H2O | nitrite; NADH; H+ | 69.85\|full | Absent | 0 | Absent | 0 | Absent | 0 |
| Nitrogen cycling | Nitrite reduction | nirK | nitrite reductase (NO-forming) | TIGR02376.hmm | K00368 | nitric oxide + H2O + ferricytochrome c = nitrite + ferrocytochrome c + 2 H+ [RN:R00783] | nitric oxide; H2O; ferricytochrome c | nitrite; ferrocytochrome c; H+ | 169.10\|full | Absent | 0 | Absent | 0 | Present | 1 |
| Nitrogen cycling | Nitrite reduction | nirS | nitrite reductase (NO-forming) / hydroxylamine reductase | nitrite_reductase_nirS.hmm | K15864 | nitric oxide + H2O + ferricytochrome c = nitrite + ferrocytochrome c + 2 H+ [RN:R00783] | nitric oxide; H2O; ferricytochrome c | nitrite; ferrocytochrome c; H+ | 200\|full | Absent | 0 | Absent | 0 | Absent | 0 |
| Nitrogen cycling | Nitrite reduction | octR | nitrite reductase (Octaheme c-type cytochrome Shewanella-type) | TIGR04315.hmm | N/A | Nitrite reduction | N/A | N/A | 325.00\|full | Absent | 0 | Absent | 0 | Absent | 0 |
| Nitrogen cycling | Nitric oxide reduction | norB | nitric oxide reductase subunit B | nitric_oxide_reductase_norB.hmm | K04561 | nitrous oxide + 2 ferricytochrome c + H2O = 2 nitric oxide + 2 ferrocytochrome c + 2 H+ [RN:R00294] | nitrous oxide; ferricytochrome c; H2O | nitric oxide; ferrocytochrome c; H+ | 79\|full | Absent | 0 | Absent | 0 | Absent | 0 |
| Nitrogen cycling | Nitric oxide reduction | norC | nitric oxide reductase subunit C | nitric_oxide_reductase_norC.hmm | K02305 | nitrous oxide + 2 ferricytochrome c + H2O = 2 nitric oxide + 2 ferrocytochrome c + 2 H+ [RN:R00294] | nitrous oxide; ferricytochrome c; H2O | nitric oxide; ferrocytochrome c; H+ | 50\|full | Present | 2 | Absent | 0 | Absent | 0 |
| Nitrogen cycling | Nitrous oxide reduction | nosD | nitrous oxidase accessory protein | PF05048.hmm | N/A | Nitrous oxide reduction | N/A | N/A | 290\|full | Absent | 0 | Absent | 0 | Absent | 0 |
| Nitrogen cycling | Nitrous oxide reduction | nosZ | nitrous-oxide reductase | TIGR04246.hmm | N/A | Nitrous oxide reduction | N/A | N/A | 550.00\|full | Absent | 0 | Absent | 0 | Absent | 0 |
| Nitrogen cycling | Anammox | hzoA | hydroxylamine dehydrogenase, alpha subunit | hydrazine_oxidase_hzoA.hmm | K20935 | Hydrazine + 4 Ferricytochrome c <=> Nitrogen + 4 Ferrocytochrome c [RN:R07174] | Hydrazine; ferricytochrome c | Nitrogen | 325\|full | Absent | 0 | Absent | 0 | Absent | 0 |
| Nitrogen cycling | Anammox | hzsA | hydrazine synthase subunit, alpha subunit | hydrazine_synthase_hzsA.hmm | K20932 | nitric oxide + ammonium + 3 ferrocytochrome c = hydrazine + H2O + 3 ferricytochrome c [RN:R09799] | nitric oxide; ammonium; ferrocytochrome c | hydrazine; H2O; ferricytochrome c | 466\|full | Absent | 0 | Absent | 0 | Absent | 0 |
| Sulfur cycling | Sulfide oxidation | fccB | flavocytochrome c sulphide dehydrogenase, flavin-binding | PF09242.hmm | K17229 | hydrogen sulfide + 2 ferricytochrome c = sulfur + 2 ferrocytochrome c + 2 H+ [RN:R09499] | hydrogen sulfide; ferricytochrome c | sulfur; ferrocytochrome c; H+ | 266\|full | Absent | 0 | Absent | 0 | Absent | 0 |
| Sulfur cycling | Sulfide oxidation | sqr | sulfide:quinone oxidoreductase | sulfide_quinone_oxidoreductase_sqr.hmm | K17218 | n HS- + n quinone = polysulfide + n quinol [RN:R10152] | HS-; quinone | polysulfide; quinol | 300\|full | Absent | 0 | Absent | 0 | Present | 1 |
| Sulfur cycling | Sulfite reduction | dsrD | dissimilatory sulfite reductase delta subunit | dsrD.hmm | N/A | Not clear | N/A | N/A | 32.80\|full | Absent | 0 | Absent | 0 | Absent | 0 |
| Sulfur cycling | Sulfur oxidation | dsrA | dissimilatory sulfite reductase alpha subunit | dsrA.hmm | K11180 | hydrogen sulfide + a [DsrC protein]-disulfide + 2 acceptor + 3 H2O = sulfite + a [DsrC protein]-dithiol + 2 reduced acceptor + 2 H+ [RN:R00861] | hydrogen sulfide; [DsrC protein]-disulfide; acceptor; H2O | sulfite; [DsrC protein]-dithiol; reduced acceptor; H+ | 200.00\|full | Absent | 0 | Absent | 0 | Absent | 0 |
| Sulfur cycling | Sulfur oxidation | dsrB | dissimilatory sulfite reductase beta subunit | dsrB.hmm | K11181 | hydrogen sulfide + a [DsrC protein]-disulfide + 2 acceptor + 3 H2O = sulfite + a [DsrC protein]-dithiol + 2 reduced acceptor + 2 H+ [RN:R00861] | hydrogen sulfide; [DsrC protein]-disulfide; acceptor; H2O | sulfite; [DsrC protein]-dithiol; reduced acceptor; H+ | 204.00\|full | Absent | 0 | Absent | 0 | Absent | 0 |
| Sulfur cycling | Sulfur oxidation | sdo | sulfur dioxygenase | sulfur_dioxygenase_sdo.hmm | K17725 | S-sulfanylglutathione + O2 + H2O = glutathione + sulfite + 2 H+ [RN:R08678] | S-sulfanylglutathione; O2; H2O | glutathione; sulfite; H+ | 300\|full | Present | 2 | Absent | 0 | Present | 2 |
| Sulfur cycling | Sulfur oxidation/reduction | sor | sulfur oxygenase/reductase | PF07682.hmm | K16952 | 4 sulfur + 4 H2O + O2 = 2 hydrogen sulfide + 2 sulfite [RN:R07365] | sulfur; H2O; O2 | hydrogen sulfide; sulfite | 120\|full | Absent | 0 | Absent | 0 | Absent | 0 |
| Sulfur cycling | Sulfur reduction | sreA | sulfur reductase molybdopterin subunit | K17219.hmm | K17219 | Sulfur + Reduced acceptor = Hydrogen sulfide + Acceptor | Sulfur, Reduced acceptor | Hydrogen sulfide, Acceptor | 1889.97\|full | Absent | 0 | Absent | 0 | Absent | 0 |
| Sulfur cycling | Sulfur reduction | sreB | sulfur reductase FeS subunit | K17220.hmm | K17220 | Sulfur + Reduced acceptor = Hydrogen sulfide + Acceptor | Sulfur, Reduced acceptor | Hydrogen sulfide, Acceptor | 486.50\|full | Absent | 0 | Absent | 0 | Absent | 0 |
| Sulfur cycling | Sulfur reduction | sreC | sulfur reductase membrane anchor | K17221.hmm | K17221 | Sulfur + Reduced acceptor = Hydrogen sulfide + Acceptor | Sulfur, Reduced acceptor | Hydrogen sulfide, Acceptor | 721.30\|full | Absent | 0 | Absent | 0 | Absent | 0 |
| Sulfur cycling | Thiosulfate oxidation | soxB | S-sulfosulfanyl-L-cysteine sulfohydrolase | soxB.hmm | K17224 | [SoxY protein]-S-sulfosulfanyl-L-cysteine + H2O = [SoxY protein]-S-sulfanyl-L-cysteine + sulfate [RN:R12096]; [SoxY protein]-S-(2-sulfodisulfanyl)-L-cysteine + H2O = [SoxY protein]-S-disulfanyl-L-cysteine + sulfate [RN:R12097] | [SoxY protein]-S-sulfosulfanyl-L-cysteine; H2O; [SoxY protein]-S-(2-sulfodisulfanyl)-L-cysteine | [SoxY protein]-S-sulfanyl-L-cysteine; sulfate; [SoxY protein]-S-disulfanyl-L-cysteine | 550.00\|full | Absent | 0 | Absent | 0 | Absent | 0 |
| Sulfur cycling | Thiosulfate oxidation | soxY | sulfur-oxidizing protein SoxY | soxY.hmm | K17226 | Thiosulfate + 5 H2O + 8 Ferricytochrome c = 2 Sulfate + 8 Ferrocytochrome c + 10 H+ | Thiosulfate; H2O; Ferricytochrome c | Sulfate; Ferrocytochrome c; H+ | 125.00\|full | Absent | 0 | Absent | 0 | Present | 1 |
| Sulfur cycling | Thiosulfate oxidation | soxC | sulfane dehydrogenase subunit SoxC | soxC.hmm | K17225 | Thiosulfate + 5 H2O + 8 Ferricytochrome c = 2 Sulfate + 8 Ferrocytochrome c + 10 H+ | Thiosulfate; H2O; Ferricytochrome c | Sulfate; Ferrocytochrome c; H+ | 320.00\|full | Absent | 0 | Absent | 0 | Absent | 0 |
| Sulfur cycling | Sulfite reduction | asrA | anaerobic sulfite reductase subunit A | asrA.hmm | K16950 | Sulfite + 3 NADH + 3 H+ <=> Hydrogen sulfide + 3 NAD+ + 3 H2O | Sulfite; NADH; H+ | Hydrogen sulfide; NAD+; H2O | 323.15\|full | Absent | 0 | Absent | 0 | Absent | 0 |
| Sulfur cycling | Sulfite reduction | asrB | anaerobic sulfite reductase subunit B | asrB.hmm | K16951 | Sulfite + 3 NADH + 3 H+ <=> Hydrogen sulfide + 3 NAD+ + 3 H2O | Sulfite; NADH; H+ | Hydrogen sulfide; NAD+; H2O | 288.60\|full | Absent | 0 | Absent | 0 | Absent | 0 |
| Sulfur cycling | Sulfite reduction | asrC | anaerobic sulfite reductase subunit C | asrC.hmm | K00385 | Sulfite + 3 NADH + 3 H+ <=> Hydrogen sulfide + 3 NAD+ + 3 H2O | Sulfite; NADH; H+ | Hydrogen sulfide; NAD+; H2O | 324.10\|full | Absent | 0 | Absent | 0 | Absent | 0 |
| Sulfur cycling | Sulfate reduction | aprA | adenylylsulfate reductase, subunit A | aprA.hmm | K00394 | AMP + sulfite + acceptor = adenylyl sulfate + reduced acceptor [RN:R00860] | AMP; sulfite; acceptor | adenylyl sulfate; reduced acceptor | 641.75\|full | Absent | 0 | Absent | 0 | Absent | 0 |
| Sulfur cycling | Sulfate reduction | sat | sulfate adenylyltransferase | TIGR00339.hmm | K00958 | ATP + sulfate = diphosphate + adenylyl sulfate [RN:R00529] | ATP; sulfate | diphosphate; adenylyl sulfate | 181.80\|full | Absent | 0 | Absent | 0 | Absent | 0 |
| Sulfur cycling | Thiosulfate disproportionation | phsA | thiosulfate reductase / polysulfide reductase chain A | thiosulfate_reductase_phsA.hmm | K08352 | thiosulfate + a quinol = sulfite + hydrogen sulfide + a quinone [RN:R11488] | thiosulfate; quinol | sulfite; hydrogen sulfide; quinone | 323\|full | Absent | 0 | Absent | 0 | Absent | 0 |
| Hydrogenases | FeFe hydrogenase | fefe-group-a13 | FeFe hydrogenase, H2-evolution/Electron-bifurcation | fefe-group-a13.hmm | N/A | H2-evolution/Electron-bifurcation | N/A | N/A | 733.8\|full | Absent | 0 | Absent | 0 | Absent | 0 |
| Hydrogenases | FeFe hydrogenase | fefe-group-a2 | FeFe hydrogenase, H2-uptake | fefe-group-a2.hmm | N/A | H2-uptake | N/A | N/A | 815.2\|full | Absent | 0 | Absent | 0 | Absent | 0 |
| Hydrogenases | FeFe hydrogenase | fefe-group-a4 | FeFe hydrogenase, H2-uptake/Electron-bifurcation | fefe-group-a4.hmm | N/A | H2-uptake/Electron-bifurcation | N/A | N/A | 599.05\|full | Absent | 0 | Absent | 0 | Absent | 0 |
| Hydrogenases | FeFe hydrogenase | fefe-group-b | FeFe hydrogenase, H2-evolution | fefe-group-b.hmm | N/A | H2-evolution | N/A | N/A | 440.3\|full | Absent | 0 | Absent | 0 | Absent | 0 |
| Hydrogenases | FeFe hydrogenase | fefe-group-c1 | FeFe hydrogenase, H2-sensing | fefe-group-c1.hmm | N/A | H2-sensing | N/A | N/A | 398.25\|full | Absent | 0 | Absent | 0 | Absent | 0 |
| Hydrogenases | FeFe hydrogenase | fefe-group-c2 | FeFe hydrogenase, H2-sensing | fefe-group-c2.hmm | N/A | H2-sensing | N/A | N/A | 483.75\|full | Absent | 0 | Absent | 0 | Absent | 0 |
| Hydrogenases | FeFe hydrogenase | fefe-group-c3 | FeFe hydrogenase, H2-sensing | fefe-group-c3.hmm | N/A | H2-sensing | N/A | N/A | 427.05\|full | Absent | 0 | Absent | 0 | Absent | 0 |
| Hydrogenases | Fe hydrogenase | fe | Fe hydrogenase, Bidirectional | fe.hmm | N/A | Bidirectional | N/A | N/A | 621\|full | Absent | 0 | Absent | 0 | Absent | 0 |
| Hydrogenases | Ni-Fe Hydrogenase | nife-group-1 | Ni-Fe Hydrogenase, H2-uptake | nife-group-1.hmm | N/A | H2-uptake | N/A | N/A | 447.25\|full | Absent | 0 | Absent | 0 | Absent | 0 |
| Hydrogenases | Ni-Fe Hydrogenase | nife-group-2ade | Ni-Fe Hydrogenase, H2-uptake | nife-group-2ade.hmm | N/A | H2-uptake | N/A | N/A | 611.8\|full | Absent | 0 | Absent | 0 | Absent | 0 |
| Hydrogenases | Ni-Fe Hydrogenase | nife-group-2bc | Ni-Fe Hydrogenase, H2-uptake | nife-group-2bc.hmm | N/A | H2-uptake | N/A | N/A | 600.3\|full | Absent | 0 | Absent | 0 | Absent | 0 |
| Hydrogenases | Ni-Fe Hydrogenase | nife-group-3abd | Ni-Fe Hydrogenase, Bidirectional | nife-group-3abd.hmm | N/A | Bidirectional | N/A | N/A | 520.25\|full | Present | 1 | Absent | 0 | Absent | 0 |
| Hydrogenases | Ni-Fe Hydrogenase | nife-group-3c | Ni-Fe Hydrogenase, Electron-bifurcation | nife-group-3c.hmm | N/A | Electron-bifurcation | N/A | N/A | 620.65\|full | Absent | 0 | Absent | 0 | Absent | 0 |
| Hydrogenases | Ni-Fe Hydrogenase | nife-group-4a-g | Ni-Fe Hydrogenase, H2-evolving | nife-group-4a-g.hmm | N/A | H2-evolving | N/A | N/A | 458\|full | Absent | 0 | Absent | 0 | Absent | 0 |
| Hydrogenases | Ni-Fe Hydrogenase | nife-group-4hi | Ni-Fe Hydrogenase, H2-uptake | nife-group-4hi.hmm | N/A | H2-uptake | N/A | N/A | 447.8\|full | Absent | 0 | Absent | 0 | Absent | 0 |
| Oxidative phosphorylation | Complex I (NADH-quinone oxidoreductase) | nuoA | NADH-quinone oxidoreductase subunit A | K00330.hmm | K00330 | NADH + ubiquinone + 6 H+[side 1] = NAD+ + ubiquinol + 7 H+[side 2] [RN:R11945] | NADH; ubiquinone; H+ | NAD+; ubiquinol; H+ | 120.27\|full | Present | 2 | Present | 1 | Present | 1 |
| Oxidative phosphorylation | Complex I (NADH-quinone oxidoreductase) | nuoB | NADH-quinone oxidoreductase subunit B | K00331.hmm | K00331 | NADH + ubiquinone + 6 H+[side 1] = NAD+ + ubiquinol + 7 H+[side 2] [RN:R11945] | NADH; ubiquinone; H+ | NAD+; ubiquinol; H+ | 263.03\|domain | Present | 2 | Present | 1 | Present | 1 |
| Oxidative phosphorylation | Complex I (NADH-quinone oxidoreductase) | nuoC | NADH-quinone oxidoreductase subunit C | K00332.hmm | K00332 | NADH + ubiquinone + 6 H+[side 1] = NAD+ + ubiquinol + 7 H+[side 2] [RN:R11945] | NADH; ubiquinone; H+ | NAD+; ubiquinol; H+ | 202.20\|domain | Absent | 0 | Absent | 0 | Present | 1 |
| Oxidative phosphorylation | Complex I (NAD(P)H-quinone oxidoreductase) | NdhA | NAD(P)H-quinone oxidoreductase subunit 1 | K05572.hmm | K05572 | NADH + ubiquinone + 6 H+[side 1] = NAD+ + ubiquinol + 7 H+[side 2] [RN:R11945] | NADH; ubiquinone; H+ | NAD+; ubiquinol; H+ | 518.33\|full | Absent | 0 | Absent | 0 | Absent | 0 |
| Oxidative phosphorylation | Complex I (NAD(P)H-quinone oxidoreductase) | NdhB | NAD(P)H-quinone oxidoreductase subunit 2 | K05573.hmm | K05573 | NADH + ubiquinone + 6 H+[side 1] = NAD+ + ubiquinol + 7 H+[side 2] [RN:R11945] | NADH; ubiquinone; H+ | NAD+; ubiquinol; H+ | 552.07\|full | Absent | 0 | Absent | 0 | Absent | 0 |
| Oxidative phosphorylation | Complex I (NAD(P)H-quinone oxidoreductase) | NdhC | NAD(P)H-quinone oxidoreductase subunit 3 | K05574.hmm | K05574 | NADH + ubiquinone + 6 H+[side 1] = NAD+ + ubiquinol + 7 H+[side 2] [RN:R11945] | NADH; ubiquinone; H+ | NAD+; ubiquinol; H+ | 173.80\|full | Absent | 0 | Absent | 0 | Absent | 0 |
| Oxidative phosphorylation | Complex II (Succinate dehydrogenase/Fumarate reductase) | sdhC | succinate dehydrogenase / fumarate reductase, cytochrome b subunit | K00241.hmm | K00241 | succinate + a quinone = fumarate + a quinol [RN:R02164] | succinate; quinone | fumarate; quinol | 43.60\|domain | Present | 1 | Absent | 0 | Present | 1 |
| Oxidative phosphorylation | Complex II (Succinate dehydrogenase/Fumarate reductase) | sdhD | succinate dehydrogenase / fumarate reductase, membrane anchor subunit | K00242.hmm | K00242 | succinate + a quinone = fumarate + a quinol [RN:R02164] | succinate; quinone | fumarate; quinol | 64.07\|domain | Absent | 0 | Absent | 0 | Present | 1 |
| Oxidative phosphorylation | Complex III (Cytochrome c reductase) | petA | ubiquinol-cytochrome c reductase iron-sulfur subunit | K00411.hmm | K00411 | quinol + 2 ferricytochrome c = quinone + 2 ferrocytochrome c + 2 H+[side 2] | quinol; ferricytochrome c | quinone; ferrocytochrome c; H+ | 84.30\|full | Absent | 0 | Absent | 0 | Present | 1 |
| Oxidative phosphorylation | Complex III (Cytochrome c reductase) | petB | ubiquinol-cytochrome c reductase cytochrome b/c1 subunit | K00412.hmm | K00412 | quinol + 2 ferricytochrome c = quinone + 2 ferrocytochrome c + 2 H+[side 2] | quinol; ferricytochrome c | quinone; ferrocytochrome c; H+ | 297.67\|domain | Present | 1 | Absent | 0 | Present | 1 |
| Oxidative phosphorylation | Complex III (Cytochrome c reductase) | fbcH | ubiquinol-cytochrome c reductase cytochrome b/c1 subunit | K00410.hmm | K00410 | quinol + 2 ferricytochrome c = quinone + 2 ferrocytochrome c + 2 H+[side 2] | quinol; ferricytochrome c | quinone; ferrocytochrome c; H+ | 718.00\|domain | Absent | 0 | Absent | 0 | Present | 1 |
| Oxidative phosphorylation | Complex V (ATP synthase: V/A-type H+/Na+-transporting ATPase) | atpA (V/A-type) | V/A-type H+/Na+-transporting ATPase subunit A | K02117.hmm | K02117 | ATP + H2O + 4 H+[side 1] = ADP + phosphate + 4 H+[side 2] | ATP; H2O; H+ | ADP; phosphate; H+ | 814.23\|full | Absent | 0 | Absent | 0 | Absent | 0 |
| Oxidative phosphorylation | Complex V (ATP synthase: V/A-type H+/Na+-transporting ATPase) | atpB (V/A-type) | V/A-type H+/Na+-transporting ATPase subunit B | K02118.hmm | K02118 | ATP + H2O + 4 H+[side 1] = ADP + phosphate + 4 H+[side 2] | ATP; H2O; H+ | ADP; phosphate; H+ | 701.83\|full | Absent | 0 | Absent | 0 | Absent | 0 |
| Oxidative phosphorylation | Complex V (F-type H+-transporting ATPase) | atpA (F-type) | F-type H+/Na+-transporting ATPase subunit alpha | K02111.hmm | K02111 | ATP + H2O + 4 H+[side 1] = ADP + phosphate + 4 H+[side 2] | ATP; H2O; H+ | ADP; phosphate; H+ | 431.80\|domain | Present | 1 | Present | 1 | Present | 1 |
| Oxidative phosphorylation | Complex V (F-type H+-transporting ATPase) | atpD (F-type) | F-type H+/Na+-transporting ATPase subunit beta | K02112.hmm | K02112 | ATP + H2O + 4 H+[side 1] = ADP + phosphate + 4 H+[side 2] | ATP; H2O; H+ | ADP; phosphate; H+ | 482.47\|domain | Present | 1 | Present | 1 | Present | 1 |
| Oxygen metabolism (Oxidative phosphorylation Complex IV) | Oxygen metabolism - cytochrome c oxidase, caa3-type | coxA | cytochrome c oxidase subunit I | TIGR02891.hmm | K02274 | 4 ferrocytochrome c + O2 + 4 H+ = 4 ferricytochrome c + 2 H2O [RN:R00081] | ferrocytochrome c; O2; H+ | ferricytochrome c; H2O | 617.40\|full | Present | 2 | Present | 2 | Present | 1 |
| Oxygen metabolism (Oxidative phosphorylation Complex IV) | Oxygen metabolism - cytochrome c oxidase, caa3-type | coxB | cytochrome c oxidase subunit II | TIGR02866.hmm | K02275 | 4 ferrocytochrome c + O2 + 4 H+ = 4 ferricytochrome c + 2 H2O [RN:R00081] | ferrocytochrome c; O2; H+ | ferricytochrome c; H2O | 144.30\|full | Present | 2 | Present | 2 | Present | 1 |
| Oxygen metabolism (Oxidative phosphorylation Complex IV) | Oxygen metabolism - cytochrome c oxidase, cbb3-type | ccoN | cytochrome c oxidase cbb3-type subunit I | TIGR00780.hmm | K00404 | 4 ferrocytochrome c + O2 + 4 H+ = 4 ferricytochrome c + 2 H2O [RN:R00081] | ferrocytochrome c; O2; H+ | ferricytochrome c; H2O | 168.15\|full | Absent | 0 | Absent | 0 | Present | 1 |
| Oxygen metabolism (Oxidative phosphorylation Complex IV) | Oxygen metabolism - cytochrome c oxidase, cbb3-type | ccoO | cytochrome c oxidase cbb3-type subunit II | TIGR00781.hmm | K00405 | 4 ferrocytochrome c + O2 + 4 H+ = 4 ferricytochrome c + 2 H2O [RN:R00081] | ferrocytochrome c; O2; H+ | ferricytochrome c; H2O | 62.85\|full | Present | 1 | Absent | 0 | Present | 1 |
| Oxygen metabolism (Oxidative phosphorylation Complex IV) | Oxygen metabolism - cytochrome c oxidase, cbb3-type | ccoP | cytochrome c oxidase cbb3-type subunit III | TIGR00782.hmm | K00406 | 4 ferrocytochrome c + O2 + 4 H+ = 4 ferricytochrome c + 2 H2O [RN:R00081] | ferrocytochrome c; O2; H+ | ferricytochrome c; H2O | 54.05\|full | Present | 1 | Present | 2 | Present | 1 |
| Oxygen metabolism (Oxidative phosphorylation Complex IV) | Oxygen metabolism - cytochrome (quinone) oxidase, bo type | cyoA | cytochrome o ubiquinol oxidase subunit II | TIGR01433.hmm | K02297 | 2 ubiquinol + O2 + n H+[side 1] = 2 ubiquinone + 2 H2O + n H+[side 2] [RN:R11335] | ubiquinol; O2; H+ | ubiquinone; H2O; H+ | 218.75\|full | Absent | 0 | Absent | 0 | Present | 1 |
| Oxygen metabolism (Oxidative phosphorylation Complex IV) | Oxygen metabolism - cytochrome (quinone) oxidase, bo type | cyoD | cytochrome o ubiquinol oxidase subunit IV | TIGR02847.hmm | K02300 | 2 ubiquinol + O2 + n H+[side 1] = 2 ubiquinone + 2 H2O + n H+[side 2] [RN:R11335] | ubiquinol; O2; H+ | ubiquinone; H2O; H+ | 64.95\|full | Absent | 0 | Absent | 0 | Present | 1 |
| Oxygen metabolism (Oxidative phosphorylation Complex IV) | Oxygen metabolism - cytochrome (quinone) oxidase, bo type | cyoB | cytochrome o ubiquinol oxidase subunit I | K02298.hmm | K02298 | 2 ubiquinol + O2 + n H+[side 1] = 2 ubiquinone + 2 H2O + n H+[side 2] [RN:R11335] | ubiquinol; O2; H+ | ubiquinone; H2O; H+ | 967.93\|full | Absent | 0 | Absent | 0 | Present | 1 |
| Oxygen metabolism (Oxidative phosphorylation Complex IV) | Oxygen metabolism - cytochrome (quinone) oxidase, bo type | cyoC | cytochrome o ubiquinol oxidase subunit III | K02299.hmm | K02299 | 2 ubiquinol + O2 + n H+[side 1] = 2 ubiquinone + 2 H2O + n H+[side 2] [RN:R11335] | ubiquinol; O2; H+ | ubiquinone; H2O; H+ | 262.30\|full | Absent | 0 | Absent | 0 | Present | 1 |
| Oxygen metabolism (Oxidative phosphorylation Complex IV) | Oxygen metabolism - cytochrome (quinone) oxidase, bd type | cydA | cytochrome bd ubiquinol oxidase subunit I | PF01654.hmm | K00425 | 2 ubiquinol + O2[side 2] + 4 H+[side 2] = 2 ubiquinone + 2 H2O[side 2] + 4 H+[side 1] [RN:R11325] | ubiquinol; O2; H+; e- | ubiquinone; H2O; H+; e- | 118\|full | Present | 1 | Present | 1 | Present | 1 |
| Oxygen metabolism (Oxidative phosphorylation Complex IV) | Oxygen metabolism - cytochrome (quinone) oxidase, bd type | cydB | cytochrome bd ubiquinol oxidase subunit II | TIGR00203.hmm | K00426 | 2 ubiquinol + O2[side 2] + 4 H+[side 2] = 2 ubiquinone + 2 H2O[side 2] + 4 H+[side 1] [RN:R11325] | ubiquinol; O2; H+; e- | ubiquinone; H2O; H+; e- | 88.55\|full | Present | 1 | Absent | 0 | Present | 1 |
| Oxygen metabolism (Oxidative phosphorylation Complex IV) | Oxygen metabolism - cytochrome (quinone) oxidase, aa3 type, QoxABCD | qoxA | cytochrome aa3-600 menaquinol oxidase subunit II | TIGR01432.hmm | K02826 | 2 menaquinol + O2 + n H+[side 1] = 2 menaquinone + 2 H2O + n H+[side 2] [RN:R09492] | menaquinol; O2; H+ | menaquinone; H2O; H+ | 204.55\|full | Absent | 0 | Absent | 0 | Absent | 0 |
| Oxygen metabolism (Oxidative phosphorylation Complex IV) | Oxygen metabolism - cytochrome (quinone) oxidase, aa3 type, QoxABCD | qoxB | cytochrome aa3-600 menaquinol oxidase subunit I | TIGR02882.hmm | K02827 | 2 menaquinol + O2 + n H+[side 1] = 2 menaquinone + 2 H2O + n H+[side 2] [RN:R09492] | menaquinol; O2; H+ | menaquinone; H2O; H+ | 954.70\|full | Absent | 0 | Absent | 0 | Absent | 0 |
| Urea utilization | Urease | ureC | urease subunit alpha | TIGR01792.hmm | K01428 | urea + H2O = CO2 + 2 NH3 [RN:R00131] | urea; H2O | CO2; NH3 | 212.95\|full | Absent | 0 | Absent | 0 | Present | 1 |
| Urea utilization | Urease | ureB | urease subunit beta | TIGR00192.hmm | K01429 | urea + H2O = CO2 + 2 NH3 [RN:R00131] | urea; H2O | CO2; NH3 | 40.65\|full | Absent | 0 | Absent | 0 | Present | 1 |
| Urea utilization | Urease | ureA | urease subunit gamma | TIGR00193.hmm | K01430 | urea + H2O = CO2 + 2 NH3 [RN:R00131] | urea; H2O | CO2; NH3 | 31.45\|full | Absent | 0 | Absent | 0 | Present | 1 |
| Halogenated compound utilization | Halogenated compounds breakdown | E3.8.1.2 | 2-haloacid dehalogenase | K01560.hmm | K01560 | (S)-2-haloacid + H2O = (R)-2-hydroxyacid + halide [RN:R03830] | (S)-2-haloacid; H2O | (R)-2-hydroxyacid; halide | 141.17\|domain | Present | 1 | Present | 1 | Present | 3 |
| Halogenated compound utilization | Halogenated compounds breakdown | pcpC | tetrachlorohydroquinone reductive dehalogenase | K15241.hmm | K15241 | 2,6-dichlorohydroquinone + Cl- + glutathione disulfide = 2,3,6-trichlorohydroquinone + 2 glutathione [RN:R05403]; 2,3,6-trichlorohydroquinone + Cl- + glutathione disulfide = 2,3,5,6-tetrachlorohydroquinone + 2 glutathione [RN:R05402] | 2,6-dichlorohydroquinone; Cl-; glutathione disulfide; 2,3,6-trichlorohydroquinone | 2,3,6-trichlorohydroquinone; glutathione; 2,3,5,6-tetrachlorohydroquinone | 276.67\|full | Absent | 0 | Absent | 0 | Absent | 0 |
| Halogenated compound utilization | Halogenated compounds breakdown | cprA | 3-chloro-4-hydroxyphenylacetate reductive dehalogenase | K21566.hmm | K21566 | 1-haloalkane + H2O = a primary alcohol + halide [RN:R02337] | 1-haloalkane; H2O | primary alcohol; halide | 850.93\|full | Absent | 0 | Absent | 0 | Absent | 0 |
| Halogenated compound utilization | Halogenated compounds breakdown | pceA | tetrachloroethene reductive dehalogenase | K21647.hmm | K21647 | trichloroethene + chloride + acceptor = tetrachloroethene + reduced acceptor [RN:R05753] | trichloroethene; chloride; acceptor | tetrachloroethene; reduced acceptor | 1150.07\|full | Absent | 0 | Absent | 0 | Absent | 0 |
| Perchlorate reduction | Perchlorate reduction | pcrA | perchlorate reductase, subunit alpha | TIGR03479.hmm | N/A | reduced acceptor + perchlorate = acceptor + H2O + chlorite | perchlorate; reduced acceptor | chlorite; acceptor; H2O | 559.05\|full | Absent | 0 | Absent | 0 | Absent | 0 |
| Perchlorate reduction | Perchlorate reduction | pcrB | perchlorate reductase, subunit beta | TIGR03478.hmm | N/A | reduced acceptor + perchlorate = acceptor + H2O + chlorite | perchlorate; reduced acceptor | chlorite; acceptor; H2O | 319.40\|full | Absent | 0 | Absent | 0 | Absent | 0 |
| Chlorite reduction | Chlorite reduction | cld | chlorite dismutase | PF06778.hmm | K09162 | chloride + O2 = chlorite [RN:R05721] | chloride; O2 | chlorite | 65\|full | Absent | 0 | Present | 1 | Absent | 0 |
| As cycling | Arsenite oxidation | aoxA | arsenite oxidase small subunit | TIGR02694.hmm | K08355 | arsenite + H2O + 2 oxidized cytochrome c = arsenate + 2 reduced cytochrome c + 2 H+ [RN:R09787] | arsenite; H2O; oxidized cytochrome c | arsenate; reduced cytochrome c; H+ | 61.70\|full | Absent | 0 | Absent | 0 | Absent | 0 |
| As cycling | Arsenite oxidation | aoxB | arsenite oxidase large subunit | TIGR02693.hmm | K08356 | arsenite + H2O + 2 oxidized cytochrome c = arsenate + 2 reduced cytochrome c + 2 H+ [RN:R09787] | arsenite; H2O; oxidized cytochrome c | arsenate; reduced cytochrome c; H+ | 357.05\|full | Absent | 0 | Absent | 0 | Absent | 0 |
| As cycling | Arsenate reduction | arsC1 | arsenate reductase (glutaredoxin) | K00537.hmm | K00537 | arsenate + glutaredoxin = arsenite + glutaredoxin disulfide + H2O [RN:R05747] | arsenate; glutaredoxin | arsenite; glutaredoxin disulfide; H2O | 83.77\|domain | Absent | 0 | Absent | 0 | Present | 2 |
| As cycling | Arsenate reduction | arsC2 | arsenate reductase (thioredoxin) | K03741.hmm | K03741 | arsenate + glutaredoxin = arsenite + glutaredoxin disulfide + H2O [RN:R05747] | arsenate; glutaredoxin | arsenite; glutaredoxin disulfide; H2O | 123.07\|domain | Present | 1 | Present | 1 | Present | 1 |
| Selenate reduction | Selenate reduction | ygfM | putative selenate reductase FAD-binding subunit | TIGR03312.hmm | K12529 | selenite + H2O + acceptor = selenate + reduced acceptor [RN:R07229] | selenite; H2O; acceptor | selenate; reduced acceptor | 87.85\|full | Absent | 0 | Absent | 0 | Absent | 0 |
| Selenate reduction | Selenate reduction | xdhD | putative selenate reductase molybdopterin-binding subunit | TIGR03313.hmm | K12528 | selenite + H2O + acceptor = selenate + reduced acceptor [RN:R07229] | selenite; H2O; acceptor | selenate; reduced acceptor | 526.50\|full | Absent | 0 | Absent | 0 | Absent | 0 |
| Selenate reduction | Selenate reduction | YgfK | putative selenate reductase | TIGR03315.hmm | K12527 | selenite + H2O + acceptor = selenate + reduced acceptor [RN:R07229] | selenite; H2O; acceptor | selenate; reduced acceptor | 354.45\|full | Absent | 0 | Absent | 0 | Absent | 0 |
| Nitrile hydration | Nitrile hydratase | nthA | nitrile hydratase subunit alpha | TIGR01323.hmm | K01721 | an aliphatic amide = a nitrile + H2O [RN:R02826] | aliphatic amide | nitrile; H2O | 239.05\|full | Absent | 0 | Present | 1 | Present | 1 |
| Nitrile hydration | Nitrile hydratase | nthB | nitrile hydratase subunit beta | TIGR03888.hmm | K20807 | an aliphatic amide = a nitrile + H2O [RN:R02826] | aliphatic amide | nitrile; H2O | 136.35\|full | Absent | 0 | Absent | 0 | Present | 1 |
| Metal reduction | Metal (Iron/Manganese) reduction | mtrC | decaheme c-type cytochrome, OmcA/MtrC family | TIGR03507.hmm | N/A | Iron/Manganese reduction | N/A | N/A | 60.05\|full | Absent | 0 | Absent | 0 | Present | 1 |
| Metal reduction | Metal (Iron/Manganese) reduction | mtrB | decaheme-associated outer membrane protein, MtrB/PioB family | TIGR03509.hmm | N/A | Iron/Manganese reduction | N/A | N/A | 165.60\|full | Present | 1 | Absent | 0 | Absent | 0 |
| Sulfur cycling enzymes (detailed) | Dissimilatory sulfur metabolism \| Sulfide oxidation | fccB | flavocytochrome c sulphide dehydrogenase, flavin-binding | PF09242.hmm | K17229 | hydrogen sulfide + 2 ferricytochrome c = sulfur + 2 ferrocytochrome c + 2 H+ [RN:R09499] | hydrogen sulfide; ferricytochrome c | sulfur; ferrocytochrome c; H+ | 266\|full | Absent | 0 | Absent | 0 | Absent | 0 |
| Sulfur cycling enzymes (detailed) | Dissimilatory sulfur metabolism \| Sulfide oxidation | sqr | sulfide:quinone oxidoreductase | sulfide_quinone_oxidoreductase_sqr.hmm | K17218 | n HS- + n quinone = polysulfide + n quinol [RN:R10152] | HS-; quinone | polysulfide; quinol | 300\|full | Absent | 0 | Absent | 0 | Present | 1 |
| Sulfur cycling enzymes (detailed) | Dissimilatory sulfur metabolism \| Sulfur oxidation/Sulfite reduction | dsrA | dissimilatory sulfite reductase alpha subunit | dsrA.hmm | N/A | hydrogen sulfide + a [DsrC protein]-disulfide + 2 acceptor + 3 H2O = sulfite + a [DsrC protein]-dithiol + 2 reduced acceptor + 2 H+ [RN:R00861] | hydrogen sulfide; [DsrC protein]-disulfide; acceptor; H2O | sulfite; [DsrC protein]-dithiol; reduced acceptor; H+ | 200.00\|full | Absent | 0 | Absent | 0 | Absent | 0 |
| Sulfur cycling enzymes (detailed) | Dissimilatory sulfur metabolism \| Sulfur oxidation/Sulfite reduction | dsrB | dissimilatory sulfite reductase beta subunit | dsrB.hmm | N/A | hydrogen sulfide + a [DsrC protein]-disulfide + 2 acceptor + 3 H2O = sulfite + a [DsrC protein]-dithiol + 2 reduced acceptor + 2 H+ [RN:R00861] | hydrogen sulfide; [DsrC protein]-disulfide; acceptor; H2O | sulfite; [DsrC protein]-dithiol; reduced acceptor; H+ | 204.00\|full | Absent | 0 | Absent | 0 | Absent | 0 |
| Sulfur cycling enzymes (detailed) | Dissimilatory sulfur metabolism \| Sulfur oxidation/Sulfite reduction | dsrC | dissimilatory sulfite reductase, subunit C | dsrC.hmm | N/A | Substrate delivery | N/A | N/A | 52.85\|full | Absent | 0 | Absent | 0 | Absent | 0 |
| Sulfur cycling enzymes (detailed) | Dissimilatory sulfur metabolism \| Sulfur oxidation/Sulfite reduction | dsrD | dissimilatory sulfite reductase delta subunit | dsrD.hmm | N/A | Not clear | N/A | N/A | 32.80\|full | Absent | 0 | Absent | 0 | Absent | 0 |
| Sulfur cycling enzymes (detailed) | Dissimilatory sulfur metabolism \| Sulfur oxidation/Sulfite reduction | dsrE | dissimilatory sulfite reductase, subunit E | dsrE.hmm | N/A | Sulfur carrier | N/A | N/A | 160.00\|full | Absent | 0 | Absent | 0 | Absent | 0 |
| Sulfur cycling enzymes (detailed) | Dissimilatory sulfur metabolism \| Sulfur oxidation/Sulfite reduction | dsrF | dissimilatory sulfite reductase, subunit F | dsrF.hmm | N/A | Sulfur carrier | N/A | N/A | 155.00\|full | Absent | 0 | Absent | 0 | Absent | 0 |
| Sulfur cycling enzymes (detailed) | Dissimilatory sulfur metabolism \| Sulfur oxidation/Sulfite reduction | dsrH | dissimilatory sulfite reductase, subunit H | dsrH.hmm | N/A | Sulfur carrier | N/A | N/A | 97.00\|full | Absent | 0 | Absent | 0 | Absent | 0 |
| Sulfur cycling enzymes (detailed) | Dissimilatory sulfur metabolism \| Sulfur oxidation/Sulfite reduction | dsrJ | dissimilatory sulfite reductase, subunit J | dsrJ.hmm | N/A | Transmembrane complex involved in electron transport | N/A | N/A | 95.00\|full | Absent | 0 | Absent | 0 | Absent | 0 |
| Sulfur cycling enzymes (detailed) | Dissimilatory sulfur metabolism \| Sulfur oxidation/Sulfite reduction | dsrK | dissimilatory sulfite reductase, subunit K | dsrK.hmm | N/A | Transmembrane complex involved in electron transport | N/A | N/A | 460.00\|full | Absent | 0 | Absent | 0 | Absent | 0 |
| Sulfur cycling enzymes (detailed) | Dissimilatory sulfur metabolism \| Sulfur oxidation/Sulfite reduction | dsrM | dissimilatory sulfite reductase, subunit M | dsrM.hmm | N/A | Transmembrane complex involved in electron transport | N/A | N/A | 172.0\|full | Absent | 0 | Absent | 0 | Absent | 0 |
| Sulfur cycling enzymes (detailed) | Dissimilatory sulfur metabolism \| Sulfur oxidation/Sulfite reduction | dsrO | dissimilatory sulfite reductase, subunit O | dsrO.hmm | N/A | Transmembrane complex involved in electron transport | N/A | N/A | 250.00\|full | Absent | 0 | Absent | 0 | Absent | 0 |
| Sulfur cycling enzymes (detailed) | Dissimilatory sulfur metabolism \| Sulfur oxidation/Sulfite reduction | dsrP | dissimilatory sulfite reductase, subunit P | dsrP.hmm | N/A | Transmembrane complex involved in electron transport | N/A | N/A | 412.80\|full | Absent | 0 | Absent | 0 | Absent | 0 |
| Sulfur cycling enzymes (detailed) | Dissimilatory sulfur metabolism \| Sulfur oxidation/Sulfite reduction | dsrR | dissimilatory sulfite reductase, subunit R | dsrR.hmm | N/A | Not clear | N/A | N/A | 80.00\|full | Absent | 0 | Absent | 0 | Absent | 0 |
| Sulfur cycling enzymes (detailed) | Dissimilatory sulfur metabolism \| Sulfur oxidation/Sulfite reduction | dsrS | dissimilatory sulfite reductase, subunit S | dsrS.hmm | N/A | Not clear | N/A | N/A | 115.70\|full | Absent | 0 | Absent | 0 | Absent | 0 |
| Sulfur cycling enzymes (detailed) | Dissimilatory sulfur metabolism \| Sulfur oxidation/Sulfite reduction | dsrT | dissimilatory sulfite reductase, subunit T | dsrT.hmm | N/A | Not clear | N/A | N/A | 90.00\|full | Absent | 0 | Absent | 0 | Absent | 0 |
| Sulfur cycling enzymes (detailed) | Dissimilatory sulfur metabolism \| Sulfite reduction | asrA | anaerobic sulfite reductase subunit A | asrA.hmm | K16950 | Sulfite + 3 NADH + 3 H+ <=> hydrogen sulfide + 3 NAD+ + 3 H2O | sulfite; NADH; H+ | hydrogen sulfide; NAD+; H2O | 323.15\|full | Absent | 0 | Absent | 0 | Absent | 0 |
| Sulfur cycling enzymes (detailed) | Dissimilatory sulfur metabolism \| Sulfite reduction | asrB | anaerobic sulfite reductase subunit B | asrB.hmm | K16951 | Sulfite + 3 NADH + 3 H+ <=> hydrogen sulfide + 3 NAD+ + 3 H2O | sulfite; NADH; H+ | hydrogen sulfide; NAD+; H2O | 288.60\|full | Absent | 0 | Absent | 0 | Absent | 0 |
| Sulfur cycling enzymes (detailed) | Dissimilatory sulfur metabolism \| Sulfite reduction | asrC | anaerobic sulfite reductase subunit C | asrC.hmm | K00385 | Sulfite + 3 NADH + 3 H+ <=> hydrogen sulfide + 3 NAD+ + 3 H2O | sulfite; NADH; H+ | hydrogen sulfide; NAD+; H2O | 324.10\|full | Absent | 0 | Absent | 0 | Absent | 0 |
| Sulfur cycling enzymes (detailed) | Dissimilatory sulfur metabolism \| Sulfur oxidation | sdo | sulfur dioxygenase | sulfur_dioxygenase_sdo.hmm | K17725 | S-sulfanylglutathione + O2 + H2O = glutathione + sulfite + 2 H+ [RN:R08678] | S-sulfanylglutathione; O2; H2O | glutathione; sulfite; H+ | 120\|full | Present | 2 | Absent | 0 | Present | 2 |
| Sulfur cycling enzymes (detailed) | Dissimilatory sulfur metabolism \| Sulfur oxidation/reduction | sor | sulfur oxygenase/reductase | PF07682.hmm | K16952 | 4 sulfur + 4 H2O + O2 = 2 hydrogen sulfide + 2 sulfite [RN:R07365] | sulfur; H2O; O2 | hydrogen sulfide; sulfite | 300\|full | Absent | 0 | Absent | 0 | Absent | 0 |
| Sulfur cycling enzymes (detailed) | Dissimilatory sulfur metabolism \| Sulfur reduction | sreA | sulfur reductase molybdopterin subunit | K17219.hmm | K17219 | sulfur + reduced acceptor = hydrogen sulfide + acceptor | sulfur, reduced acceptor | hydrogen sulfide, acceptor | 1889.97\|full | Absent | 0 | Absent | 0 | Absent | 0 |
| Sulfur cycling enzymes (detailed) | Dissimilatory sulfur metabolism \| Sulfur reduction | sreB | sulfur reductase FeS subunit | K17220.hmm | K17220 | sulfur + reduced acceptor = hydrogen sulfide + acceptor | sulfur, reduced acceptor | hydrogen sulfide, acceptor | 486.50\|full | Absent | 0 | Absent | 0 | Absent | 0 |
| Sulfur cycling enzymes (detailed) | Dissimilatory sulfur metabolism \| Sulfur reduction | sreC | sulfur reductase membrane anchor | K17221.hmm | K17221 | sulfur + reduced acceptor = hydrogen sulfide + acceptor | sulfur, reduced acceptor | hydrogen sulfide, acceptor | 721.30\|full | Absent | 0 | Absent | 0 | Absent | 0 |
| Sulfur cycling enzymes (detailed) | Dissimilatory sulfur metabolism \| Thiosulfate oxidation | soxA | L-cysteine S-thiosulfotransferase | soxA.hmm | K17222 | [SoxY protein]-L-cysteine + thiosulfate + 2 ferricytochrome c = [SoxY protein]-S-sulfosulfanyl-L-cysteine + 2 ferrocytochrome c + 2 H+ [RN:R12163]; [SoxY protein]-S-sulfanyl-L-cysteine + thiosulfate + 2 ferricytochrome c = [SoxY protein]-S-(2-sulfodisulfanyl)-L-cysteine + 2 ferrocytochrome c + 2 H+ [RN:R12164] | [SoxY protein]-L-cysteine; thiosulfate [CPD:C00320]; ferricytochrome c [CPD:C00125]; [SoxY protein]-S-sulfanyl-L-cysteine | [SoxY protein]-S-sulfosulfanyl-L-cysteine; ferrocytochrome c; H+; [SoxY protein]-S-(2-sulfodisulfanyl)-L-cysteine | 110.00\|full | Absent | 0 | Absent | 0 | Present | 2 |
| Sulfur cycling enzymes (detailed) | Dissimilatory sulfur metabolism \| Thiosulfate oxidation | soxB | S-sulfosulfanyl-L-cysteine sulfohydrolase | soxB.hmm | K17224 | [SoxY protein]-S-sulfosulfanyl-L-cysteine + H2O = [SoxY protein]-S-sulfanyl-L-cysteine + sulfate [RN:R12096]; [SoxY protein]-S-(2-sulfodisulfanyl)-L-cysteine + H2O = [SoxY protein]-S-disulfanyl-L-cysteine + sulfate [RN:R12097] | [SoxY protein]-S-sulfosulfanyl-L-cysteine; H2O; [SoxY protein]-S-(2-sulfodisulfanyl)-L-cysteine | [SoxY protein]-S-sulfanyl-L-cysteine; sulfate; [SoxY protein]-S-disulfanyl-L-cysteine | 550.00\|full | Absent | 0 | Absent | 0 | Absent | 0 |
| Sulfur cycling enzymes (detailed) | Dissimilatory sulfur metabolism \| Thiosulfate oxidation | soxC | sulfane dehydrogenase subunit SoxC | soxC.hmm | K17225 | thiosulfate + 5 H2O + 8 ferricytochrome c = 2 sulfate + 8 ferrocytochrome c + 10 H+ | thiosulfate; H2O; ferricytochrome c | sulfate; ferrocytochrome c; H+ | 320.00\|full | Absent | 0 | Absent | 0 | Absent | 0 |
| Sulfur cycling enzymes (detailed) | Dissimilatory sulfur metabolism \| Thiosulfate oxidation | soxD | S-disulfanyl-L-cysteine oxidoreductase SoxD | soxD.hmm | K22622 | [SoxY protein]-S-disulfanyl-L-cysteine + 6 ferricytochrome c + 3 H2O = [SoxY protein]-S-sulfosulfanyl-L-cysteine + 6 ferrocytochrome c + 6 H+ [RN:R11971] | [SoxY protein]-S-disulfanyl-L-cysteine; ferricytochrome c; H2O | [SoxY protein]-S-sulfosulfanyl-L-cysteine; ferrocytochrome c [CPD:C00126]; H+ | 90.00\|full | Absent | 0 | Absent | 0 | Present | 2 |
| Sulfur cycling enzymes (detailed) | Dissimilatory sulfur metabolism \| Thiosulfate oxidation | soxX | L-cysteine S-thiosulfotransferase | soxX.hmm | K17223 | [SoxY protein]-L-cysteine + thiosulfate + 2 ferricytochrome c = [SoxY protein]-S-sulfosulfanyl-L-cysteine + 2 ferrocytochrome c + 2 H+ [RN:R12163]; [SoxY protein]-S-sulfanyl-L-cysteine + thiosulfate + 2 ferricytochrome c = [SoxY protein]-S-(2-sulfodisulfanyl)-L-cysteine + 2 ferrocytochrome c + 2 H+ [RN:R12164] | [SoxY protein]-L-cysteine; thiosulfate; ferricytochrome c; [SoxY protein]-S-sulfanyl-L-cysteine | [SoxY protein]-S-sulfosulfanyl-L-cysteine; ferrocytochrome c; H+; [SoxY protein]-S-(2-sulfodisulfanyl)-L-cysteine | 61.00\|full | Present | 2 | Absent | 0 | Present | 2 |
| Sulfur cycling enzymes (detailed) | Dissimilatory sulfur metabolism \| Thiosulfate oxidation | soxY | sulfur-oxidizing protein SoxY | soxY.hmm | K17226 | thiosulfate + 5 H2O + 8 ferricytochrome c = 2 sulfate + 8 ferrocytochrome c + 10 H+ | thiosulfate; H2O; ferricytochrome c | sulfate; ferrocytochrome c; H+ | 125.00\|full | Absent | 0 | Absent | 0 | Present | 1 |
| Sulfur cycling enzymes (detailed) | Dissimilatory sulfur metabolism \| Thiosulfate oxidation | soxYZ | soxYZ_like_carrier | soxYZ_like_carrier.hmm | N/A | quinoprotein dehydrogenase-associated SoxYZ-like carrier | N/A | N/A | 120.00\|full | Absent | 0 | Absent | 0 | Present | 1 |
| Sulfur cycling enzymes (detailed) | Dissimilatory sulfur metabolism \| Thiosulfate oxidation | soxZ | sulfur-oxidizing protein SoxZ | soxZ.hmm | K17227 | thiosulfate + 5 H2O + 8 ferricytochrome c = 2 sulfate + 8 ferrocytochrome c + 10 H+ | thiosulfate; H2O; ferricytochrome c | sulfate; ferrocytochrome c; H+ | 76.00\|full | Absent | 0 | Absent | 0 | Present | 2 |
| Sulfur cycling enzymes (detailed) | Dissimilatory sulfur metabolism \| Sulfate reduction | aprA | adenylylsulfate reductase, subunit A | aprA.hmm | K00394 | AMP + sulfite + acceptor = adenylyl sulfate + reduced acceptor [RN:R00860] | AMP; sulfite; acceptor | adenylyl sulfate; reduced acceptor | 641.75\|full | Absent | 0 | Absent | 0 | Absent | 0 |
| Sulfur cycling enzymes (detailed) | Dissimilatory sulfur metabolism \| Sulfate reduction | aprB | adenylylsulfate reductase, subunit B | aprB.hmm | K00395 | AMP + sulfite + acceptor = adenylyl sulfate + reduced acceptor [RN:R00860] | AMP; sulfite; acceptor | adenylyl sulfate; reduced acceptor | 135.65\|full | Absent | 0 | Absent | 0 | Absent | 0 |
| Sulfur cycling enzymes (detailed) | Dissimilatory sulfur metabolism \| Sulfate reduction | sat | sulfate adenylyltransferase | TIGR00339.hmm | K00958 | ATP + sulfate = diphosphate + adenylyl sulfate [RN:R00529] | ATP; sulfate | diphosphate; adenylyl sulfate | 181.80\|full | Absent | 0 | Absent | 0 | Absent | 0 |
| Sulfur cycling enzymes (detailed) | Dissimilatory sulfur metabolism \| Thiosulfate disproportionation | phsA | thiosulfate reductase / polysulfide reductase chain A | thiosulfate_reductase_phsA.hmm | K08352 | thiosulfate + a quinol = sulfite + hydrogen sulfide + a quinone [RN:R11488] | thiosulfate; quinol | sulfite; hydrogen sulfide; quinone | 323\|full | Absent | 0 | Absent | 0 | Absent | 0 |
| Sulfur cycling enzymes (detailed) | Assimilatory sulfur metabolism\| Sulfate <-> APS (Adenosine 5'-phosphosulfate) | cysD | sulfate adenylyltransferase subunit 2 | K00957.hmm | K00957 | ATP + sulfate = diphosphate + adenylyl sulfate [RN:R00529] | ATP; sulfate | diphosphate; adenylyl sulfate | 190.13\|domain | Absent | 0 | Present | 1 | Present | 1 |
| Sulfur cycling enzymes (detailed) | Assimilatory sulfur metabolism\| Sulfate <-> APS (Adenosine 5'-phosphosulfate) | cysN | sulfate adenylyltransferase subunit 1 | K00956.hmm | K00956 | ATP + sulfate = diphosphate + adenylyl sulfate [RN:R00529] | ATP; sulfate | diphosphate; adenylyl sulfate | 537.13\|domain | Absent | 0 | Present | 1 | Absent | 0 |
| Sulfur cycling enzymes (detailed) | Assimilatory sulfur metabolism\| Sulfate <-> APS (Adenosine 5'-phosphosulfate) | cysNC | bifunctional enzyme CysN/CysC | K00955.hmm | K00955 | ATP + sulfate = diphosphate + adenylyl sulfate [RN:R00529] | ATP; sulfate | diphosphate; adenylyl sulfate | 736.20\|full | Absent | 0 | Absent | 0 | Absent | 0 |
| Sulfur cycling enzymes (detailed) | Assimilatory sulfur metabolism\| APS (Adenosine 5'-phosphosulfate) <-> PAPS (3'-Phosphoadenosine 5'-phosphosulfate) | cysC | adenylylsulfate kinase | K00860.hmm | K00860 | ATP + adenylyl sulfate = ADP + 3'-phosphoadenylyl sulfate [RN:R00509] | ATP; adenylyl sulfate | ADP; 3'-phosphoadenylyl sulfate | 330.53\|domain | Absent | 0 | Absent | 0 | Absent | 0 |
| Sulfur cycling enzymes (detailed) | Assimilatory sulfur metabolism\| APS (Adenosine 5'-phosphosulfate) <-> PAPS (3'-Phosphoadenosine 5'-phosphosulfate) | cysNC | bifunctional enzyme CysN/CysC | K00955.hmm | K00955 | ATP + adenylyl sulfate = ADP + 3'-phosphoadenylyl sulfate [RN:R00509] | ATP; adenylyl sulfate | ADP; 3'-phosphoadenylyl sulfate | 736.20\|full | Absent | 0 | Absent | 0 | Absent | 0 |
| Sulfur cycling enzymes (detailed) | Assimilatory sulfur metabolism\| PAPS (3'-Phosphoadenosine 5'-phosphosulfate) <-> Sulfite | cysH | phosphoadenosine phosphosulfate reductase | K00390.hmm | K00390 | 3'-phosphoadenosine 5'-phosphosulfate + sulfite + thioredoxin disulfide = 3'-phosphoadenylyl sulfate + thioredoxin [RN:R02021] | 3'-Phosphoadenosine 5'-phosphosulfate; sulfite; thioredoxin disulfide | 3'-phosphoadenylyl sulfate; thioredoxin | 163.33\|full | Absent | 0 | Present | 1 | Present | 1 |
| Sulfur cycling enzymes (detailed) | Assimilatory sulfur metabolism\| Sulfite -> Sulfide | cysJ | sulfite reductase (NADPH) flavoprotein alpha-component | K00380.hmm | K00380 | hydrogen sulfide + 3 NADP+ + 3 H2O = sulfite + 3 NADPH + 3 H+ [RN:R00858] | hydrogen sulfide; NADP+ | sulfite; 3 NADPH | 520.13\|full | Absent | 0 | Absent | 0 | Absent | 0 |
| Sulfur cycling enzymes (detailed) | Assimilatory sulfur metabolism\| Sulfide -> Cysteine | cysK | cysteine synthase | K01738.hmm | K01738 | O-acetyl-L-serine + hydrogen sulfide = L-cysteine + acetate [RN:R00897] | O-acetyl-L-serine; hydrogen sulfide | L-cysteine; acetate | 424.83\|domain | Present | 1 | Absent | 0 | Present | 2 |
| Sulfur cycling enzymes (detailed) | Assimilatory sulfur metabolism\| Sulfide -> Cysteine | cysM | S-sulfo-L-cysteine synthase (O-acetyl-L-serine-dependent) | K12339.hmm | K12339 | O-acetyl-L-serine + hydrogen sulfide = L-cysteine + acetate [RN:R00897] | O-acetyl-L-serine; hydrogen sulfide | L-cysteine; acetate | 410.00\|domain | Absent | 0 | Absent | 0 | Absent | 0 |
| Sulfur cycling enzymes (detailed) | DMSO metabolism \| DMSO (Dimethyl sulfoxide) -> Methanesulfonate | dsoA | DMS oxygenase alpha subunit | PF06099.hmm | N/A | dimethyl sulfoxide + NADH + O2 + H+ = dimethyl sulfone + NAD+ + H2O | dimethyl sulfoxide; NADH; O2 | dimethyl sulfone; NAD+ | 50.4\|full | Absent | 0 | Absent | 0 | Absent | 0 |
| Sulfur cycling enzymes (detailed) | DMSO metabolism \| DMSO (Dimethyl sulfoxide) -> Methanesulfonate | dsoB | DMS oxygenase beta subunit | PF02332.hmm | N/A | dimethyl sulfoxide + NADH + O2 + H+ = dimethyl sulfone + NAD+ + H2O | dimethyl sulfoxide; NADH; O2 | dimethyl sulfone; NAD+ | 160.4\|full | Absent | 0 | Absent | 0 | Absent | 0 |
| Sulfur cycling enzymes (detailed) | DMSO metabolism \| DMSO (Dimethyl sulfoxide) -> Methanesulfonate | sfnG | dimethylsulfone monooxygenase | K17228.hmm | K17228 | dimethyl sulfone + FMNH2 + O2 = methanesulfinate + formaldehyde + FMN + H2O [RN:R11457] | dimethyl sulfone; FMNH2; O2 | methanesulfinate; formaldehyde; FMN | 348.03\|domain | Present | 1 | Absent | 0 | Absent | 0 |
| Sulfur cycling enzymes (detailed) | DMSO metabolism \| DMSO (Dimethyl sulfoxide) -> Methanesulfonate | msuD | methanesulfonate monooxygenase | PF00296.hmm | N/A | methanesulfonate + FMNH2 + O2 = formaldehyde + sulfite + FMN + H2O + 2 H+ | methanesulfonate; FMNH2; O2 | formaldehyde; sulfite; FMN | 264.3\|full | Absent | 0 | Present | 1 | Absent | 0 |
| Sulfur cycling enzymes (detailed) | DMSO metabolism \| DMSO (Dimethyl sulfoxide) <-> DMS (Dimethyl sulfide) | dsoA | DMS oxygenase alpha subunit | PF06099.hmm | N/A | dimethyl sulfoxide + NADH + O2 + H+ = dimethyl sulfone + NAD+ + H2O | dimethyl sulfoxide; NADH; O2 | dimethyl sulfone; NAD+ | 50.4\|full | Absent | 0 | Absent | 0 | Absent | 0 |
| Sulfur cycling enzymes (detailed) | DMSO metabolism \| DMSO (Dimethyl sulfoxide) <-> DMS (Dimethyl sulfide) | dsoB | DMS oxygenase beta subunit | PF02332.hmm | N/A | dimethyl sulfoxide + NADH + O2 + H+ = dimethyl sulfone + NAD+ + H2O | dimethyl sulfoxide; NADH; O2 | dimethyl sulfone; NAD+ | 160.4\|full | Absent | 0 | Absent | 0 | Absent | 0 |
| Sulfur cycling enzymes (detailed) | DMSO metabolism \| DMSO (Dimethyl sulfoxide) <-> DMS (Dimethyl sulfide) | ddhA | dimethylsulfide dehydrogenase subunit alpha | K16964.hmm | K16964 | dimethyl sulfide + 2 ferricytochrome c2 + H2O = dimethyl sulfoxide + 2 ferrocytochrome c2 + 2 H+ [RN:R09500] | dimethyl sulfide; 2 ferricytochrome c2 | dimethyl sulfoxide; 2 ferrocytochrome c2 | 50\|full | Absent | 0 | Absent | 0 | Present | 2 |
| Sulfur cycling enzymes (detailed) | DMSO metabolism \| DMSO (Dimethyl sulfoxide) <-> DMS (Dimethyl sulfide) | dmsA | anaerobic dimethyl sulfoxide reductase subunit A | K07306.hmm | K07306 | dimethyl sulfide + menaquinone + H2O = dimethyl sulfoxide + menaquinol [RN:R09501] | dimethyl sulfide; menaquinone | dimethyl sulfoxide; menaquinol | 628.77\|domain | Absent | 0 | Absent | 0 | Absent | 0 |
| Sulfur cycling enzymes (detailed) | DMSO metabolism \| DMS (Dimethyl sulfide) <-> Methanethiol | dmoA | dimethyl-sulfide monooxygenase subunit A | K16967.hmm | K16967 | dimethyl sulfide + O2 + NADH + H+ = methanethiol + formaldehyde + NAD+ + H2O [RN:R09786] | dimethyl sulfide; O2; NADH | methanethiol; formaldehyde; NAD+ | 50\|full | Absent | 0 | Absent | 0 | Absent | 0 |
| Sulfur cycling enzymes (detailed) | DMSO metabolism \| DMS (Dimethyl sulfide) <-> Methanethiol | dmoB | dimethyl-sulfide monooxygenase subunit B | PF01613.hmm | N/A | dimethyl sulfide + O2 + NADH + H+ = methanethiol + formaldehyde + NAD+ + H2O [RN:R09786] | dimethyl sulfide; O2; NADH | methanethiol; formaldehyde; NAD+ | 50.1\|full | Absent | 0 | Present | 1 | Present | 5 |
| Sulfur cycling enzymes (detailed) | DMSO metabolism \| DMS (Dimethyl sulfide) <-> Methanethiol | mddA | methanethiol S-methyltransferase | K21310.hmm | K21310 | S-adenosyl-L-methionine + methanethiol = S-adenosyl-L-homocysteine + dimethyl sulfide [RN:R11546] | S-adenosyl-L-methionine; methanethiol | S-adenosyl-L-homocysteine; dimethyl sulfide | 86.03\|full | Present | 1 | Absent | 0 | Absent | 0 |
| Sulfur cycling enzymes (detailed) | Sulfite production from organic sulfur \| Taurine -> Sulfite | tauD | taurine dioxygenase | K03119.hmm | K03119 | taurine + 2-oxoglutarate + O2 = sulfite + aminoacetaldehyde + succinate + CO2 [RN:R05320] | taurine; 2-oxoglutarate; O2 | sulfite; aminoacetaldehyde; succinate; CO2 | 328.90\|domain | Absent | 0 | Absent | 0 | Absent | 0 |
| Sulfur cycling enzymes (detailed) | Sulfite production from organic sulfur \| Alkylsulfonate/Isethionate -> Sulfite | ssuD | alkanesulfonate monooxygenase | K04091.hmm | K04091 | alkanesulfonate + FMNH2 + O2 = aldehyde + FMN + sulfite + H2O [RN:R07210] | alkanesulfonate; FMNH2; O2 | aldehyde; FMN; sulfite | 339.20\|full | Absent | 0 | Absent | 0 | Absent | 0 |
| Sulfur cycling enzymes (detailed) | Sulfite production from organic sulfur \| Methanesulfonate -> Sulfite | msmA | methanesulfonate monooxygenase | K16968.hmm | K16968 | methanesulfonate + NADH + H+ + O2 = formaldehyde + NAD+ + sulfite + H2O [RN:R09513] | methanesulfonate; NADH; O2 | formaldehyde; NAD+; sulfite | 482.50\|full | Absent | 0 | Absent | 0 | Absent | 0 |
| Sulfur cycling enzymes (detailed) | Metabolism of organic sulfur \| Cysteine <-> Cystathionine | metB | cystathionine gamma-synthase | K01739.hmm | K01739 | O4-succinyl-L-homoserine + L-cysteine = L-cystathionine + succinate [RN:R03260] | O4-succinyl-L-homoserine; L-cysteine | L-cystathionine; succinate | 457.87\|domain | Present | 1 | Present | 1 | Absent | 0 |
| Sulfur cycling enzymes (detailed) | Metabolism of organic sulfur \| Cysteine <-> 3-mercaptopyruvate | aspB | aspartate aminotransferase | K00812.hmm | K00812 | L-aspartate + 2-oxoglutarate = oxaloacetate + L-glutamate [RN:R00355] | L-aspartate; 2-oxoglutarate | oxaloacetate; L-glutamate | 464.33\|full | Present | 1 | Absent | 0 | Present | 1 |
| Sulfur cycling enzymes (detailed) | Metabolism of organic sulfur \| OAHS (O-acetylhomoserine) + Sulfide -> Homocysteine | metY | O-acetylhomoserine (thiol)-lyase | K01740.hmm | K01740 | O-acetyl-L-homoserine + methanethiol = L-methionine + acetate [RN:R00651] | O-acetyl-L-homoserine; methanethiol | L-methionine; acetate | 398.37\|domain | Absent | 0 | Present | 1 | Present | 4 |
| Sulfur cycling enzymes (detailed) | Metabolism of organic sulfur \| Cystathionine -> Homocysteine | malY | cysteine-S-conjugate beta-lyase | K14155.hmm | K14155 | L-cystathionine + H2O = L-homocysteine + pyruvate + ammonium | L-cystathionine | L-homocysteine; pyruvate; ammonium | 285.60\|full | Absent | 0 | Absent | 0 | Absent | 0 |
| Sulfur cycling enzymes (detailed) | Metabolism of organic sulfur \| Cystathionine -> Homocysteine | metC | cysteine-S-conjugate beta-lyase | K01760.hmm | K01760 | L-cystathionine + H2O = L-homocysteine + pyruvate + ammonium | L-cystathionine | L-homocysteine; pyruvate; ammonium | 490.93\|domain | Present | 1 | Present | 1 | Absent | 0 |
| Sulfur cycling enzymes (detailed) | Metabolism of organic sulfur \| MHO (L-methionine (R)-S-oxide) -> Methionine) | msrC | L-methionine (R)-S-oxide reductase | K08968.hmm | K08968 | L-methionine-(R)-S-oxide + reduced thioredoxin = L-methionine + oxidized thioredoxin + H2O | L-methionine-(R)-S-oxide; reduced thioredoxin | L-methionine; oxidized thioredoxin | 90.67\|domain | Present | 3 | Absent | 0 | Absent | 0 |
| Sulfur cycling enzymes (detailed) | Metabolism of organic sulfur \| Homocysteine -> Methionine | metH | 5-methyltetrahydrofolate--homocysteine methyltransferase | K00548.hmm | K00548 | 5-methyltetrahydrofolate + L-homocysteine = tetrahydrofolate + L-methionine [RN:R00946] | 5-methyltetrahydrofolate; L-homocysteine | tetrahydrofolate; L-methionine | 272.87\|domain | Present | 1 | Present | 1 | Present | 1 |
| Sulfur cycling enzymes (detailed) | Metabolism of organic sulfur \| Homocysteine <-> Methionine | metE | 5-methyltetrahydropteroyltriglutamate--homocysteine methyltransferase | K00549.hmm | K00549 | 5-methyltetrahydropteroyltri-L-glutamate + L-homocysteine = tetrahydropteroyltri-L-glutamate + L-methionine [RN:R04405] | 5-methyltetrahydropteroyltri-L-glutamate; L-homocysteine | tetrahydropteroyltri-L-glutamate; L-methionine | 36.93\|full | Absent | 0 | Absent | 0 | Present | 5 |
| Sulfur cycling enzymes (detailed) | Metabolism of organic sulfur \| SAH (S-adenosyl-L-homocysteine) -> Homocysteine | ahcY | adenosylhomocysteinase | K01251.hmm | K01251 | S-adenosyl-L-homocysteine + H2O = L-homocysteine + adenosine [RN:R00192] | S-adenosyl-L-homocysteine | L-homocysteine; adenosine | 73.23\|full | Present | 1 | Present | 1 | Present | 1 |
| Sulfur cycling enzymes (detailed) | Metabolism of organic sulfur \| SRH (S-ribosylhomocysteine) -> Homocysteine | luxS | S-ribosylhomocysteine lyase | K07173.hmm | K07173 | S-(5-deoxy-D-ribos-5-yl)-L-homocysteine = L-homocysteine + (4S)-4,5-dihydroxypentan-2,3-dione [RN:R01291] | S-(5-deoxy-D-ribos-5-yl)-L-homocysteine | L-homocysteine; (4S)-4,5-dihydroxypentan-2,3-dione | 109.23\|full | Absent | 0 | Absent | 0 | Absent | 0 |
| Sulfur cycling enzymes (detailed) | Metabolism of organic sulfur \| SAH (S-adenosyl-L-homocysteine) -> SRH (S-ribosylhomocysteine) | mtnN | adenosylhomocysteine nucleosidase | K01243.hmm | K01243 | S-adenosyl-L-homocysteine + H2O = S-ribosyl-L-homocysteine + adenine | S-adenosyl-L-homocysteine | S-ribosyl-L-homocysteine; adenine | 107.13\|domain | Absent | 0 | Absent | 0 | Absent | 0 |
| Sulfur cycling enzymes (detailed) | Sulfur-related amino acid metabolism \| Cysteine -> Sulfide | cysK | cysteine synthase | K01738.hmm | K01738 | O-acetyl-L-serine + hydrogen sulfide = L-cysteine + acetate [RN:R00897] | O-acetyl-L-serine; hydrogen sulfide | L-cysteine; acetate | 424.83\|domain | Present | 1 | Absent | 0 | Present | 2 |
| Sulfur cycling enzymes (detailed) | Sulfur-related amino acid metabolism \| Cysteine -> Sulfide | cysM | S-sulfo-L-cysteine synthase (O-acetyl-L-serine-dependent) | K12339.hmm | K12339 | O-acetyl-L-serine + hydrogen sulfide = L-cysteine + acetate [RN:R00897] | O-acetyl-L-serine; hydrogen sulfide | L-cysteine; acetate | 410.00\|domain | Absent | 0 | Absent | 0 | Absent | 0 |
| Sulfur cycling enzymes (detailed) | Sulfur-related amino acid metabolism \| Cysteine -> Sulfide | malY | cysteine-S-conjugate beta-lyase | K14155.hmm | K14155 | L-cystathionine + H2O = L-homocysteine + pyruvate + ammonium | L-cystathionine | L-homocysteine; pyruvate; ammonium | 285.60\|full | Absent | 0 | Absent | 0 | Absent | 0 |
| Sulfur cycling enzymes (detailed) | Sulfur-related amino acid metabolism \| Cysteine -> Sulfide | dcyD | D-cysteine desulfhydrase | K05396.hmm | K05396 | D-cysteine + H2O = sulfide + NH3 + pyruvate [RN:R01874] | D-cysteine | sulfide; NH3; pyruvate | 359.70\|full | Absent | 0 | Absent | 0 | Absent | 0 |
| Sulfur cycling enzymes (detailed) | Sulfur-related amino acid metabolism \| Cysteine -> Sulfide | metC | cysteine-S-conjugate beta-lyase | K01760.hmm | K01760 | L-cysteine + H2O = pyruvate + hydrogen sulfide + ammonium | L-cysteine | pyruvate; hydrogen sulfide; ammonium | 490.93\|domain | Present | 1 | Present | 1 | Absent | 0 |
| Sulfur cycling enzymes (detailed) | Sulfur-related amino acid metabolism \| Methionine -> Methanethiol | megL | methionine gamma-lyase | PF01053.hmm | N/A | L-methionine + H2O = methanethiol + 2-oxobutanoate + ammonium | L-methionine | methanethiol; 2-oxobutanoate; ammonium | 432.5\|full | Present | 1 | Present | 1 | Present | 3 |
| Sulfur cycling enzymes (detailed) | Sulfur-related amino acid metabolism \| Methionine -> SAMe (S-adenosyl-L-methionine) | metK | S-adenosylmethionine synthetase | K00789.hmm | K00789 | ATP + L-methionine + H2O = phosphate + diphosphate + S-adenosyl-L-methionine [RN:R00177] | ATP; L-methionin | phosphate; diphosphate; S-adenosyl-L-methionine | 18.10\|full | Present | 1 | Present | 1 | Present | 1 |
| Sulfur cycling enzymes (detailed) | Sulfur-related amino acid metabolism \| SAMe (S-adenosyl-L-methionine) -> SAH (S-adenosyl-L-homocysteine) | dcm | DNA (cytosine-5)-methyltransferase 1 | K00558.hmm | K00558 | S-adenosyl-L-methionine + DNA containing cytosine = S-adenosyl-L-homocysteine + DNA containing 5-methylcytosine [RN:R00380] | S-adenosyl-L-methionine; DNA containing cytosine | S-adenosyl-L-homocysteine; DNA containing 5-methylcytosine | 150.10\|full | Present | 1 | Absent | 0 | Absent | 0 |
