# Supplementary material for: Antimony efflux underpins phosphorus cycling and resistance of phosphate-solubilizing bacteria in mining soils
Source: ISME J. 2023 Jun 3;17(8):1278–89. doi: 10.1038/s41396-023-01445-6 (PMC10356851; doi:10.1038/s41396-023-01445-6)
Supplement: Supplementary file 5 — Supplementary table S4 [file 41396_2023_1445_MOESM5_ESM.docx]

**Table S4** Summary of mobile genetic elements detected in the recovered MAGs in this study.

| Genome | Phylum | Contig | MGE class | gene | *acr3*-harboring MAGs |
| --- | --- | --- | --- | --- | --- |
| HL4_bin.15 | Proteobacteria | NODE_21_length_24054 | transposase | *tnpA* | Yes |
| HH3_bin.9 | Proteobacteria | k141_27084_length_4088 | transposase | *tnpA* | Yes |
| HH3_bin.17 | Proteobacteria | NODE_14_length_51193 | istB | *istB* | No |
| HH1_bin.7 | Cyanobacteria | k141_765055_length_1531 | transposase | *tnpA* | Yes |
| HH1_bin.28 | Proteobacteria | k141_102658_length_51945 | transposase | *tnpA* | Yes |
|  |  |  | transposase | *tnpA* |  |
|  |  |  | transposase | *tnpA* |  |
|  |  |  | transposase | *tnpA* |  |
|  |  | k141_2215001_length_2792 | transposase | *tnpA2* |  |
| HH4_bin.23 | Proteobacteria | k141_302747_length_42494 | transposase | *tnpA* | Yes |
|  |  |  | transposase | *tnpA* |  |
|  |  |  | transposase | *tnpA* |  |
| VL3_bin.19 | Proteobacteria | NODE_54_length_18596 | transposase | *tnpA* | Yes |
|  |  |  | transposase | *tnpA* |  |
| VL5_bin.38 | Proteobacteria | k141_1085628_length_51542 | transposase | *tnpA* | Yes |
|  |  |  | transposase | *tnpA* |  |
|  |  |  | transposase | *tnpA* |  |
|  |  |  | transposase | *tnpA* |  |
| VH3_bin.10 | Gemmatimonadota | k141_330128_length_1607 | insertion_element_IS91 | *IS91* | No |
|  |  | k141_377938_length_1554 | insertion_element_IS91 | *IS91* |  |
| VH4_bin.12 | Proteobacteria | NODE_169_length_2053 | insertion_element_IS91 | *IS91* | Yes |
| VH4_bin.29 | Gemmatimonadota | k141_1354809_length_2110 | insertion_element_IS91 | *IS91* | No |
